# Gallium: A Universal Promoter Switching CO_2_ Methanation Catalysts to Produce Methanol

**DOI:** 10.1021/jacsau.4c00893

**Published:** 2024-12-20

**Authors:** Wei Zhou, Colin Hansen, Weicheng Cao, Enzo Brack, Scott R. Docherty, Christian Ehinger, Yuhao Wang, Chunliang Wang, Christophe Copéret

**Affiliations:** † Department of Chemistry and Applied Biosciences, ETH Zürich, CH-8093 Zurich, Switzerland; ‡ Engineering Research Center of Metallurgical Energy Conservation and Emission Reduction, Ministry of Education, Kunming University of Science and Technology, Kunming 650093, China

**Keywords:** CO_2_ hydrogenation, SOMC, gallium promoter, methanation suppression, methanol synthesis

## Abstract

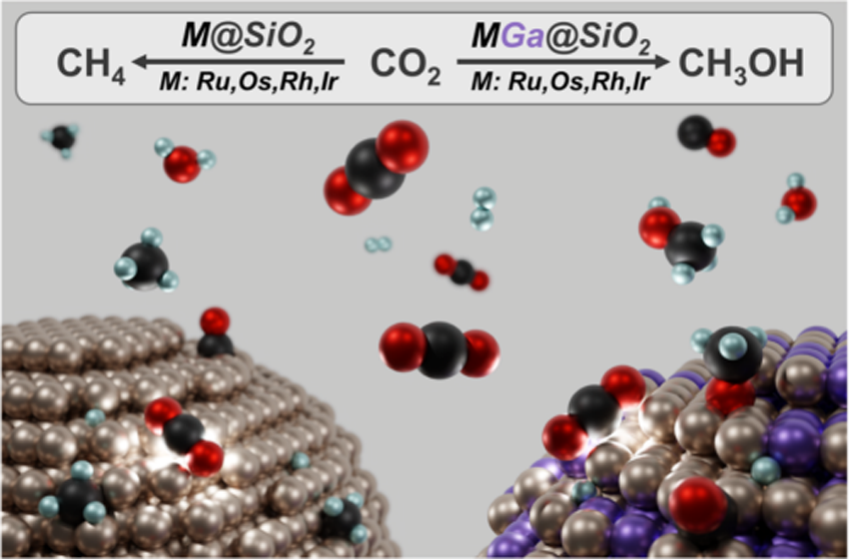

Hydrogenation of CO_2_ to methanol is foreseen as a key step to close the carbon cycle. In this study, we show that introducing Ga into silica-supported nanoparticles based on group 8–9 transition noble metals (M = Ru, Os, Rh, and Ir – *M*Ga@SiO_2_) switches their reactivity from producing mostly methane (sel. > 97%) to producing methanol (>50% CH_3_OH/DME sel.) alongside CO as the only byproduct. These silica-supported catalysts, prepared *via* a surface organometallic chemistry (SOMC) approach, consist of small, alloyed, and narrowly distributed *M*Ga nanoparticles, as evidenced by X-ray absorption spectroscopy (XAS) and CO adsorption studies. Notably, detailed *in situ* XAS and diffuse reflectance Fourier transform infrared spectroscopy (DRIFTS) studies complemented with density functional theory (DFT) calculations indicate that Ga generates stable bulk *M*Ga alloys. The bulk *M*Ga alloys persist during CO_2_ hydrogenation according to XAS, resulting in suppressed methanation. Meanwhile, a small fraction of surface GaO_*x*_ and thereby *M*Ga–GaO_*x*_ interfaces are formed, as evidenced by IR spectroscopy, likely responsible for stabilizing methoxy intermediates and favoring methanol formation.

## Introduction

CO_2_ hydrogenation based on green H_2_ is a key technology, which is foreseen to enable a more sustainable chemical industry. This process can yield various products, ranging from CO, *via* the so-called reverse water gas shift (RWGS) reaction,^1,2^ to methane by the Sabatier reaction.^3^ It can also yield chemical intermediates like methanol or even higher hydrocarbon or alcohol products *via* the Fischer–Tropsch process.^4−7^ However, the hydrogenation of CO_2_ toward value-added chemicals like methanol is notoriously more challenging when compared to the hydrogenation of CO, despite both processes sharing common catalytically active materials, reaction conditions, and similar reaction mechanisms.^8,9^ The most prominent example is the hydrogenation of CO or CO_2_ using Cu-based catalysts, which favor methanol synthesis *via* formate and methoxy intermediates, while hardly producing methane under a wide range of conditions.^10^ Aiming at improving the catalytic performance, large research efforts have been directed at identifying so-called promoters to improve product selectivity. In Cu-based materials, Zn is well-known to greatly enhance methanol selectivity.^10,11^ Other “reducible” elements, in particular Ga, have also been shown to enhance the methanol selectivity of Cu-based catalysts.^12−14^ Notably, Ga is also able to switch the general reaction outcome. For instance, Ni-catalysts, which are very well-known for their selectivity toward methane under CO_2_ hydrogenation conditions, produce methanol at low pressure in the presence of Ga.^15−17^ More recently, Ga was reported to even switch the RWGS activity of noble metals like Pd and Pt to methanol formation with exceptional activity.^18−21^ Detailed investigations for the PdGa and PtGa-systems using surface organometallic chemistry (SOMC) have shown that redox dynamics involving *M*Ga–GaO_*x*_ (M = Pd or Pt) interfaces are responsible for promoting methanol formation with high activity.^20,21^

Considering the well-known methanation activity of group 8–9 transition metals (Ru, Os, Rh, and Ir),^22−25^ we thus reasoned that Ga could be used to direct the reactivity patterns and the structure of these metals to CO_2_ hydrogenation. Toward this goal, we prepare a series of well-defined, silica-supported *M*Ga@SiO_2_ bimetallic catalysts (*M* = Ru, Os, Rh, and Ir) *via* surface organometallic chemistry, a synthetic approach that generates catalyst structures amenable to detailed (*in situ*) spectroscopic characterization. Notably, all of the prepared bimetallic catalysts show good selectivity for methanol/DME in CO_2_ hydrogenation (selectivity >50%), suppressing the methanation activity of the corresponding monometallic cases (*M*@SiO_2_ catalysts, that typically show CH_4_ selectivity >97%). Detailed *in situ* studies indicate that Ga readily forms alloyed *M*Ga nanoparticles for these metals upon H_2_ reduction. Under CO_2_ hydrogenation conditions, the bulk *M*Ga alloy persists, resulting in suppressing methanation, while a small fraction of *M*Ga–GaO_*x*_ interfaces evidenced by IR spectroscopy are formed and are likely responsible for promoting methanol formation. This study highlights the universal propensity of Ga in promoting methanol formation across a broad range of metals, including classical methanation catalysts.

## Results

All materials, *M*Ga@SiO_2_, (M = Ru, Os, Rh, and Ir), are prepared *via* the SOMC approach in order to enable better control of the composition and the interface between the metal and the support.^26,27^ The synthesis involves the grafting of a molecular precursor on Ga-decorated silica, which contains isolated surface −OH groups and Ga^III^ surface sites (Ga^III^@SiO_2_, 0.8 Ga^III^ nm^–2^). This support material is prepared by grafting [Ga(OSi(O^t^Bu)_3_)_3_(THF)] on silica, partially dehydroxylated at 700 °C (SiO_2–700_, 0.9 −OH nm^–2^), and a subsequent thermal treatment under high vacuum (10^–5^ mbar) to remove all organic residues (Figure S1 and S2).^28^

**Figure 1:**
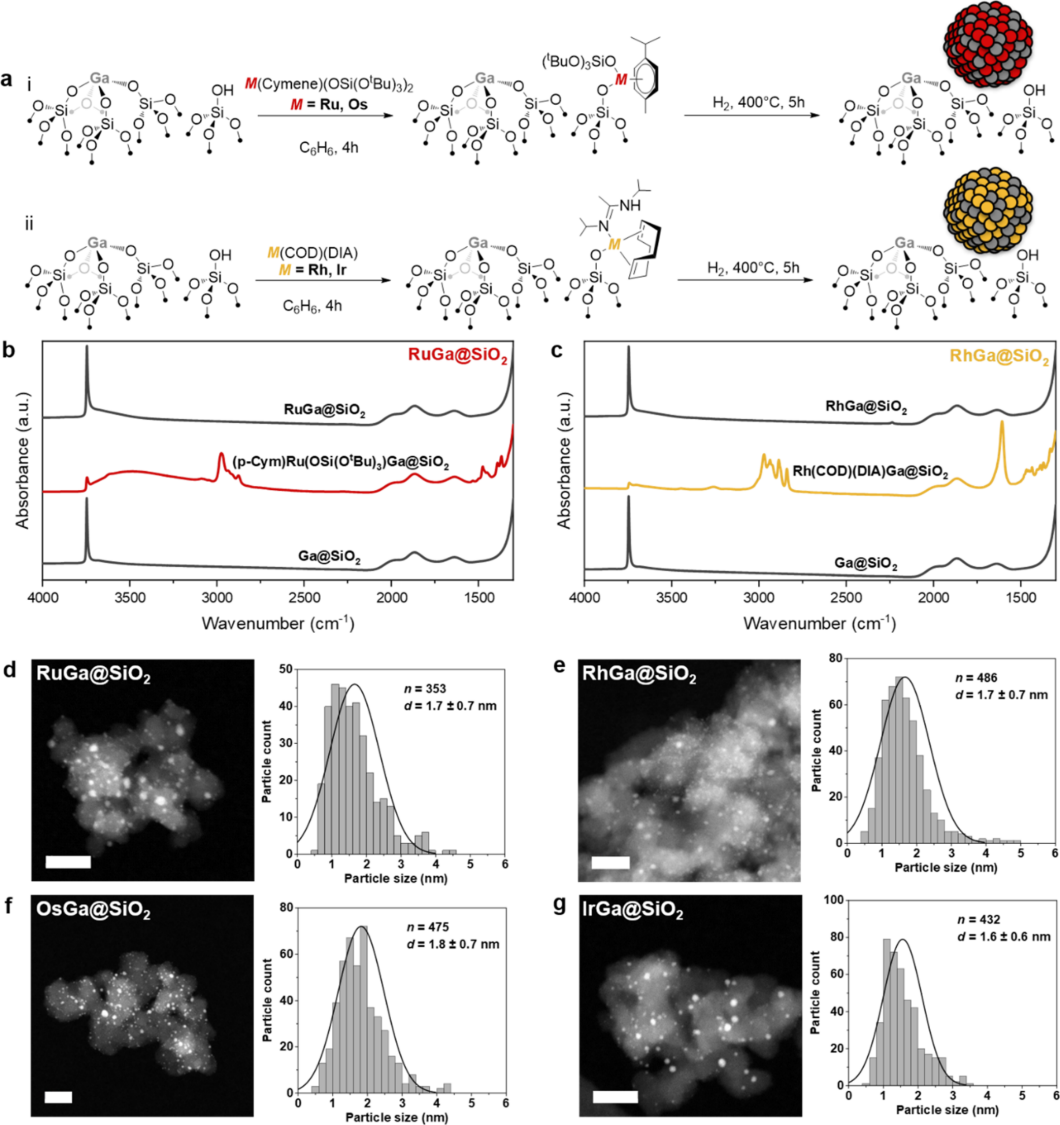
Preparation of *M*Ga@SiO_2_ and *M*@SiO_2_ materials. (a) Schematic procedure for grafting group 8 (Ru, Os) and group 9 (Rh, Ir) molecular precursors on Ga^III^@SiO_2_, followed by reduction under 1 bar of H_2_ at 400 °C. IR spectra throughout the synthesis of RuGa@SiO_2_ (b) and RhGa@SiO_2_ (c) starting from the second grafting. High-angle annular dark-field STEM (HAADF-STEM) images and particle size distribution of RuGa@SiO_2_ (d), RhGa@SiO_2_ (e), OsGa@SiO_2_ (f), and IrGa@SiO_2_ (g). The scale bar is 20 nm.

Then, a metal precursor (Ru, Os, Rh, and Ir) is grafted on Ga^III^@SiO_2_, and H_2_ treatment of the resulting bimetallic material provides metallic silica-supported nanoparticles (Figure 1a, see the Supporting Information (SI) for experimental details). The group 8 (Ru/Os) and group 9 (Rh/Ir) metal precursors used for grafting are based on *M*(*p*-cymene)(OSi(O*^t^*Bu)_3_)_2_ (Figures S3–S5)^29^ and *M*(COD)(DIA) (COD = 1,5-*cis,cis*-cyclooctadiene, DIA = *N*,*N*′-diisopropylacetamidinate) (Figures S6–S12), respectively.^30^ These precursors were chosen because they can readily react with surface OH groups to generate highly dispersed metal sites. In a subsequent treatment under a flow of H_2_ at 400 °C, these materials readily generate supported nanoparticles free of organic ligands as evidenced by IR spectroscopy (Figures 1b,c, S13, and S14). The corresponding monometallic materials *M*@SiO_2_ (*M* = Ru, Os, Rh, or Ir) are also prepared through the same approach using SiO_2–700_ in place of Ga^III^@SiO_2_ (Figures S15–S18).

The metal loadings in all materials, as determined by inductively coupled plasma optical emission spectroscopy (ICP-OES), are comparable among each other (*i.e.*, within a metal density range of 0.50–0.85 *M*/nm^2^) with *M*/Ga ratios close to 1 for *M*Ga@SiO_2_ materials (Table S1). HAADF-STEM micrographs show a narrow particle size distribution centered at 1.6–1.8 nm for all four bimetallic *M*Ga@SiO_2_ materials (Figure 1d–g). Note that the nanoparticles are smaller for *M*Ga@SiO_2_ than for the corresponding *M*@SiO_2_, indicating a strong interaction between the transition metal *M* and the Ga promoter (Figures S21–S23 and Table S1). Energy-dispersive X-ray spectroscopy (EDX) maps show that the Ga and *M* profiles overlap in all cases, indicating the formation of *M*Ga alloys upon H_2_ reduction (Figures S26–S29). In fact, IR spectra of the samples exposed to CO show a significant red shift of the adsorbed CO on *M*Ga@SiO_2_*vsM*@SiO_2_ (Figure S30),^31,32^ providing further evidence for alloying between *M* and Ga.

**Figure 2:**
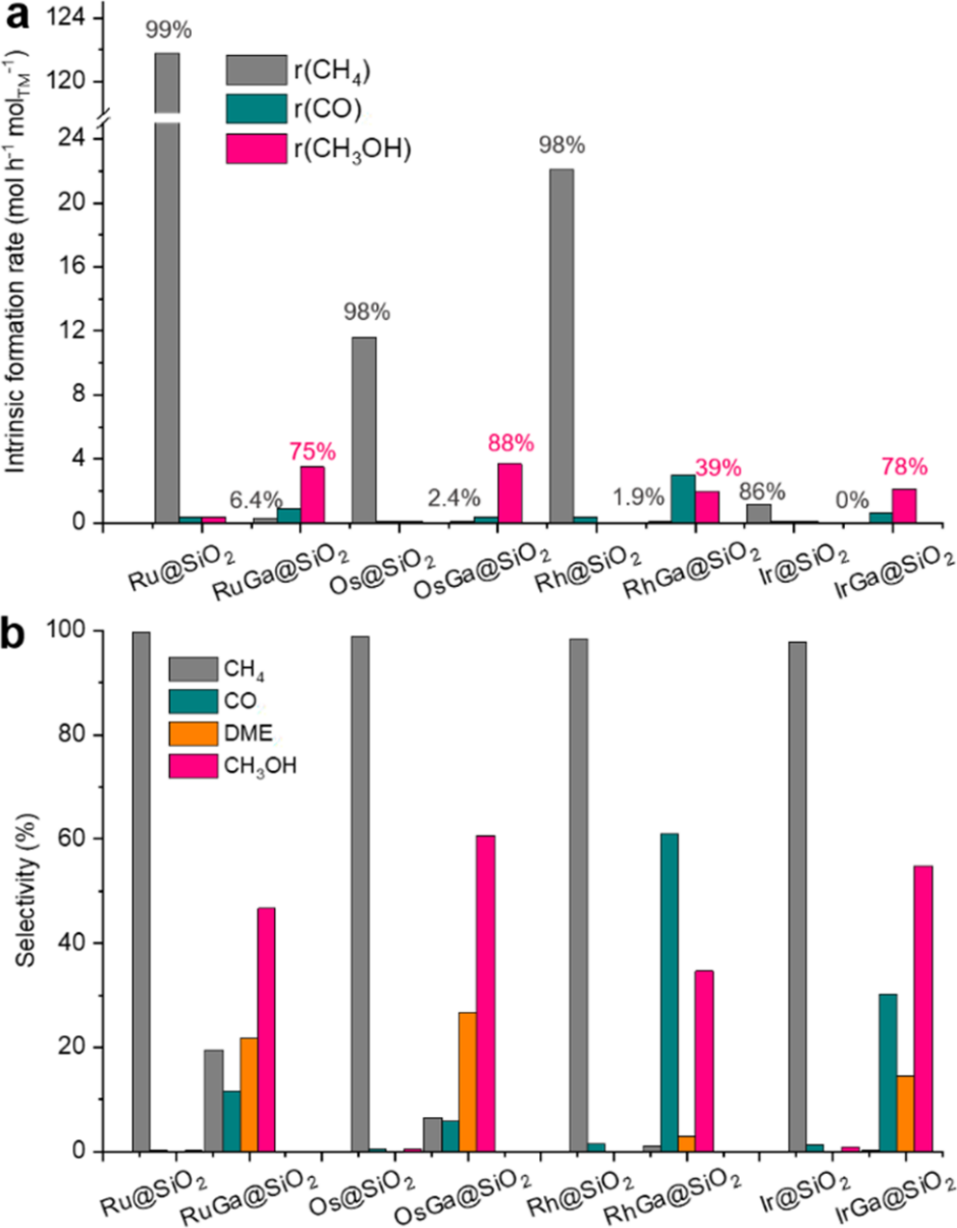
Catalytic performance of CO_2_ hydrogenation over investigated catalysts. (a) Intrinsic formation rates over monometallic *M*@SiO_2_ and bimetallic *M*Ga@SiO_2_ catalysts. (b) Product selectivities of monometallic *M*@SiO_2_ and bimetallic *M*Ga@SiO_2_ catalysts at a CO_2_ conversion of 1%. Reaction conditions: *F* = 6–100 mL/min, *T* = 230 °C, and *P* = 40 bar.

*M*Ga@SiO_2_ and *M*@SiO_2_ materials are next evaluated in CO_2_ hydrogenation at 230 °C and 40 bar (H_2_/CO_2_/Ar = 3:1:1). Kinetic information related to product formation is obtained by altering the gas flow rates throughout the experiments. Under the given reaction conditions, methanol is the main product for all of the bimetallic catalysts, while monometallic systems produce essentially only methane, with Ga@SiO_2_ being inactive (below detection limit) (Figures S31–S38). The intrinsic methanol formation rates over RuGa@SiO_2_, OsGa@SiO_2_, RhGa@SiO_2_, and IrGa@SiO_2_ are 2.0, 3.7, 2.0, and 2.1 mol h^–1^ mol_TM_^–1^, respectively, which compare well with other reported CO_2_ hydrogenation catalysts prepared by SOMC (Table S3). These rates correspond to intrinsic methanol selectivities of 65, 88, 39, and 78%, respectively (Figure 2a and Tables S2 and S3). RhGa@SiO_2_ displays the lowest methanol selectivity of 39%, which can be enhanced to approximately 50% upon tuning the Rh/Ga ratio (Figures S19, S20, S24, S25, S39–S42, and Table S2). In sharp contrast to bimetallic systems, the corresponding monometallic catalysts, Ru@SiO_2_, Os@SiO_2_ Rh@SiO_2_, and Ir@SiO_2_, produce mainly methane with relatively high activity and selectivity, as expected for these metals (Figure 2a and Table S2). It thus becomes evident that Ga significantly suppresses methanation activity while promoting methanol formation.

Comparing the catalysts at 1% CO_2_ conversion shows that the methane selectivity sharply decreases from >97% for monometallic *M*@SiO_2_ catalysts to below 20% for Ga-promoted *M*Ga@SiO_2_ systems (Figure 2b and Table S4). All four *M*Ga@SiO_2_ catalysts also produce CO and a small amount of dimethyl ether (DME). The latter is likely formed by the dehydration of CH_3_OH over Lewis acidic Ga-sites.^12,20^ Overall, the selectivity toward CH_3_OH/DME at 1% CO_2_ conversion is 60, 87, 38, and 70% for RuGa@SiO_2_, OsGa@SiO_2_, RhGa@SiO_2_, and IrGa@SiO_2_, respectively.

**Figure 3:**
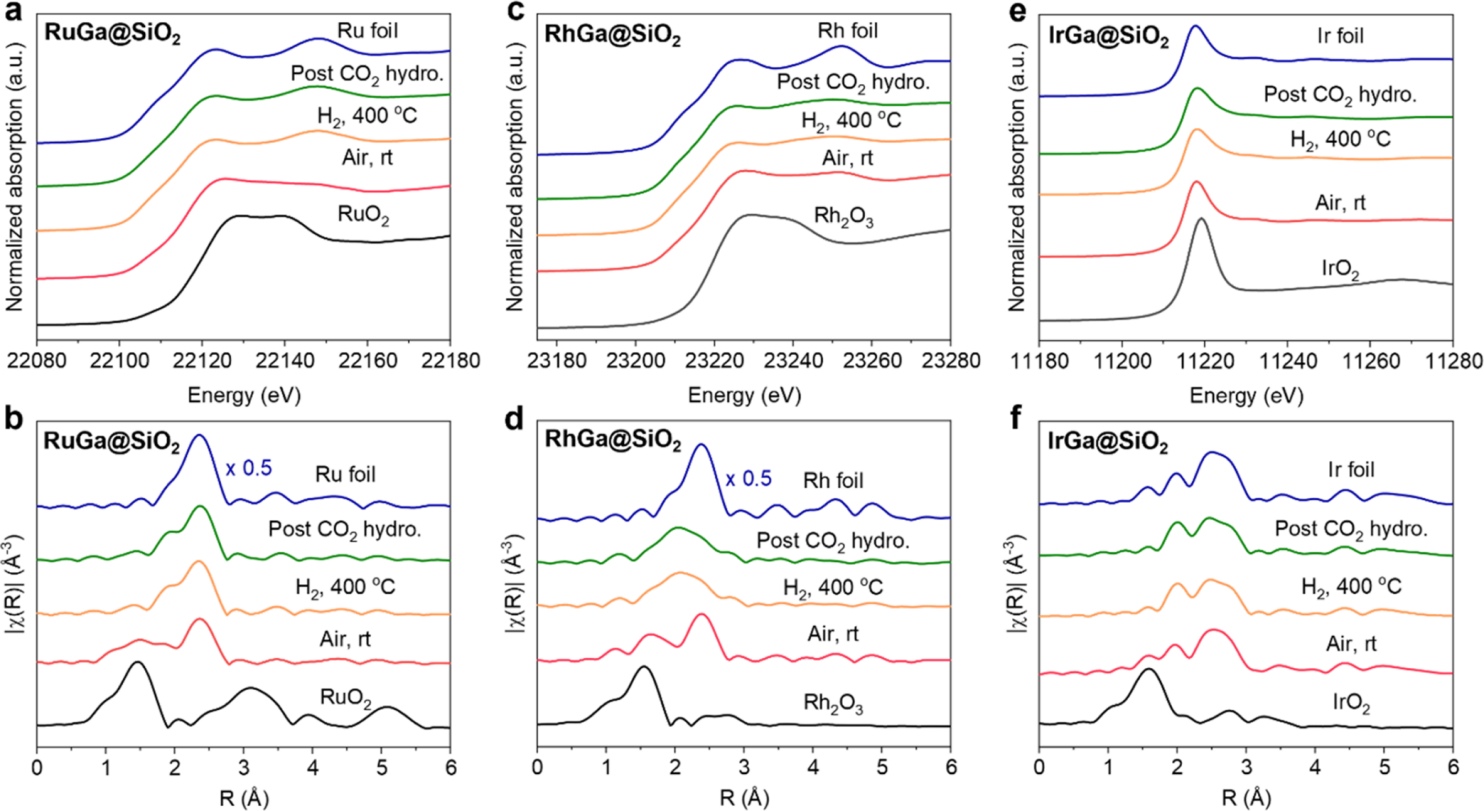
*In situ* XAS studies. XANES spectra and the *k*^2^-weighted Fourier transforms of EXAFS spectra under different conditions for RhGa@SiO_2_ at the Rh *K*-edge (a, b); RuGa@SiO_2_ at the Ru *K*-edge (c, d); IrGa@SiO_2_ at the Ir *L*_3_-edge (e, f), respectively.

To understand the apparent universal promotional effect of Ga, the evolution of the structure and chemical properties of all catalysts, except for the Os-based systems, is investigated by *in situ* X-ray absorption spectroscopy (XAS) experiments. In order to do so, XAS spectra are recorded under different conditions (exposed to air, reduced in H_2_, and post-CO_2_ hydrogenation) (Figure S43). The Ga-promoted bimetallic (*M*Ga@SiO_2_, M = Ru, Rh, and Ir) catalysts are investigated at the metal *K*- or *L*_3_-edge and compared with the monometallic *M*@SiO_2_. The corresponding best fits are shown in Figures S44–S58 and summarized in Tables S5–S7. The X-ray absorption near-energy structure (XANES) spectra of the Ru and Rh K-edge for the air-exposed RuGa@SiO_2_ and RhGa@SiO_2_ catalysts display resemblances to the metal oxide references, while the XANES spectrum at the Ir *L*_3_-edge and the corresponding white line intensity for the air-exposed IrGa@SiO_2_ are comparable to metallic Ir foil. A similar difference is observed for the corresponding air-exposed monometallic *M*@SiO_2_ catalysts: the XANES spectrum of Ru@SiO_2_ is consistent with a slightly oxidized metal (Figures S59 and S60), while Rh@SiO_2_ and Ir@SiO_2_ remain metallic (Figures S61–64). The extended X-ray absorption fine structure (EXAFS) spectra of both air-exposed RuGa@SiO_2_ and RhGa@SiO_2_ catalysts reveal a coordination number (CN) of 3.7 and 5.0 for Ru–O and Ru–Ru and a CN of 2.2 and 4.0 for Rh–O and Rh–Rh, respectively (Figures 3a–d, S47, S50, Tables S5, and S6), indicating a mixture of metallic and oxidized species, consistent with what is observed in XANES. For IrGa@SiO_2_, the EXAFS fitting shows a CN of 10.4 for the Ir–Ir scattering path, while no Ir–O path is observed. This further supports the exclusive presence of metallic Ir in the air-exposed IrGa@SiO_2_ as found in XANES (Figures 3e,f, S56, and Table S7).

Next, XANES was used to monitor the evolution of the air-exposed materials throughout H_2_ temperature-programmed reduction (TPR). With increasing temperatures, the white line intensities at the *K*-edges of both Ru and Rh gradually decrease, and the edge position gradually shifts to lower energy, indicating the reduction of Ru and Rh in both RuGa@SiO_2_ and RhGa@SiO_2_ (Figure S65a,b). In fact, after H_2_ reduction at 400 °C, the peaks associated with metal oxides observed in the Fourier transform of the EXAFS spectra disappear, which is again consistent with reduction. Additionally, new peaks appear in the *R*-space between the ones expected for *M*–O and *M*–*M* (*M* = Ru or Rh) (Figure 3b,3d), and the EXAFS fitting results for both systems show an average CN of 1.6 and 6.2 for Ru–Ga and Ru–Ru and an average CN of 3.6 and 2.3 for Rh–Ga and Rh–Rh, indicating alloying in both cases (Figures S48, S51, Table S5, and S6). For IrGa@SiO_2_, the Ir *L*_3_-edge XANES spectrum indicates a stepwise shift to higher energy along with a decrease in the white line intensity during H_2_ TPR (Figure S65c), which is indicative of a change in structure for Ir due to the presence of Ga. After H_2_ reduction, examination of the EXAFS spectrum shows that the peak at *ca.* 2.0 Å in *R*-space becomes more intense compared to the second peak at *ca.* 2.5 Å, while this change is not observed in monometallic Ir@SiO_2_ (Figures 3f and S64). These observations are consistent with the interaction between Ga and Ir, and EXAFS fitting reveals the presence of a Ir–Ir scattering path (CN_Ir–Ir_ = 9.2) as well as a Ir–Ga path (CN_Ir–Ga_ = 0.9) (Figure S57 and Table S7), thereby confirming IrGa alloy formation upon reduction. Note that after the H_2_ reduction of the monometallic *M*@SiO_2_ materials at 400 °C, the XAS spectra are consistent with the exclusive presence of metallic nanoparticles (Figures S59–S64 and S66). EXAFS fitting results after CO_2_ hydrogenation indicate similar CN for *M*Ga in all three *M*Ga@SiO_2_ systems, revealing that the *M*Ga alloy persists throughout the CO_2_ hydrogenation reaction (Figure 3 and Tables S5–S7). Note that for the monometallic systems, *M* in *M*@SiO_2_ remaining fully metallic is well-known for methanation catalysts (Figures S59–S64 and Tables S5–S7).^25^

In addition, *in situ* Ga *K*-edge XAS spectra were also acquired under the same conditions in order to gain more insights into the interplay between *M* (*M* = Ru, Rh, and Ir) and Ga in the *M*Ga@SiO_2_ systems. In all cases, the XANES spectra at the Ga *K*-edge for the air-exposed *M*Ga@SiO_2_ systems are similar to Ga^III^@SiO_2_ (Figures S67–S69), while the edge position shifts to lower energy, and the white line intensity decreases during H_2_-TPR (Figure S70). These data suggest that Ga^III^ is gradually reduced to Ga^0^ and incorporated into the nanoparticles to form alloyed *M*Ga nanoparticles after H_2_ treatment (vide supra). A linear combination fitting (LCF) analysis of the spectra after H_2_ reduction enables us to evaluate the composition of the *M*Ga nanoparticles (Figure S71 and Table S8): the average ratios of *M*/Ga^0^ are 3.0:1, 1.6:1, and 2.4:1 in RuGa-, RhGa-, and IrGa-alloyed nanoparticles, respectively, indicating that the *M*Ga alloys contain *ca.* 25–40% of Ga^0^. Furthermore, all of the Ga *K*-edge EXAFS spectra can be fitted using a model that includes Ga–O and Ga-*M* paths after H_2_ reduction (Figures S72–S80 and Table S9). Both the *MK*- or *L*_3_-edge and Ga *K*-edge spectra demonstrate the formation of a *M*Ga alloy after H_2_ reduction. Similar to what is found at the corresponding metal edges, no change is observed at the Ga *K*-edge under CO_2_ hydrogenation (Table S9), consistent with the stability of the alloy under these conditions. Overall, the XAS study at both the *M* and Ga edges shows that alloying between the metal and Ga is favored under H_2_ and that the alloys are stable under CO_2_ hydrogenation conditions. This observation parallels the reactivity switch from methanation over monometallic catalysts to methanol formation over *M*Ga alloys.

*In situ* diffuse reflectance IR Fourier transform spectroscopy (DRIFTS) experiments were performed to monitor intermediate species over the Ga-promoted *M*Ga@SiO_2_ catalysts (M = Ru, Os, Rh, and Ir) and monometallic *M*@SiO_2_ during CO_2_ hydrogenation at 20 bar. Notably, when the bimetallic catalysts are exposed to a gas mixture of H_2_/CO_2_/Ar (3:1:1–20 bar), two CO IR bands at 2040–2070 and 1930–2000 cm^–1^ appear, indicating the presence of two families of CO surface sites. A recent report on related silica-supported PtGa catalysts has indicated that these two species are likely associated with CO* absorbed on *M*Ga and *M*Ga–GaO_*x*_ interfaces.^21^ Considering that the formation of *M*Ga–GaO_*x*_ interfaces is not observed by XAS, its amount is small and likely associated with surface sites that promote methanol formation, as previously reported for other Ga-promoted systems.^17,21^ In addition, a band at 2170 cm^–1^ (gaseous CO) and a band at 3015 cm^–1^ (gaseous CH_4_) can be observed for the *M*Ga@SiO_2_ catalysts (Figure S81).^33^ Besides the observed bands for gaseous CO and CH_4_ and adsorbed CO*, two bands at around 2960 and 2858 cm^–1^ are also detected. These two bands can be assigned to methoxy (CH_3_O*) species at the surface of the catalysts, which are key intermediates for methanol formation.^20,34^ These methoxy species are not observed over monometallic Ru@SiO_2_ and Rh@SiO_2_ catalysts. However, a rather high-intensity band at 3015 cm^–1^ and weak rotational bands between 2600 and 3200 cm^–1^ are observed due to a high concentration of gaseous CH_4_ (Figure S82).^35^ This is consistent with the observed high methanation activity in CO_2_ hydrogenation over Ru@SiO_2_ and Rh@SiO_2_. The same trend is also observed over the Os-based and Ir-based catalysts. Note that slight methoxy bands can be detected after 30 min over Ir@SiO_2_, which agrees with the observation that Ir@SiO_2_ shows a non-negligible 7% intrinsic methanol selectivity (Figure 2a and Table S2).

To better understand the affinity of these metals toward alloying with Ga and how alloying suppresses methanation, we further explore simple descriptors for alloying and for the capacity toward C–O bond cleavage on pure metals *vs* alloyed surfaces using density functional theory (DFT) modeling. The affinity of the various metals toward Ga is evaluated by calculating the alloy formation enthalpy (Δ*H*_alloying_). In addition to the metals investigated in this study, we include Ni as a benchmark because it is a well-known methanation catalyst, for which the presence of Ga has been shown to have similar promotional effects, yielding methanol under CO_2_ hydrogenation conditions.^17^ Osmium, however, has been excluded due to the lack of experimental data (no XAS data, *vide supra*) and established work function data sets.

For all of the systems, a *M*/Ga (*M* = Ru, Rh, Ir, and Ni) ratio of 3:1, close to the experimental ratio quantified by LCF analysis, is used for modeling the alloys. The most stable face centered cubic (FCC) phase is used as a reference because it is the most stable metallic state across the selected metals. This phase is chosen to evaluate the alloy formation enthalpy (Δ*H*_alloying_) by the energy difference between the metal and the corresponding alloy with a similar structure. In all four *M*Ga bimetallic systems, the values of Δ*H*_alloying_ are negative (Figures S83–S87 and Table S10), indicating that Ga is readily incorporated into the *M* phase to form stable *M*Ga alloys for these metals, aligning with the *in situ* XAS results.

**Figure 4:**
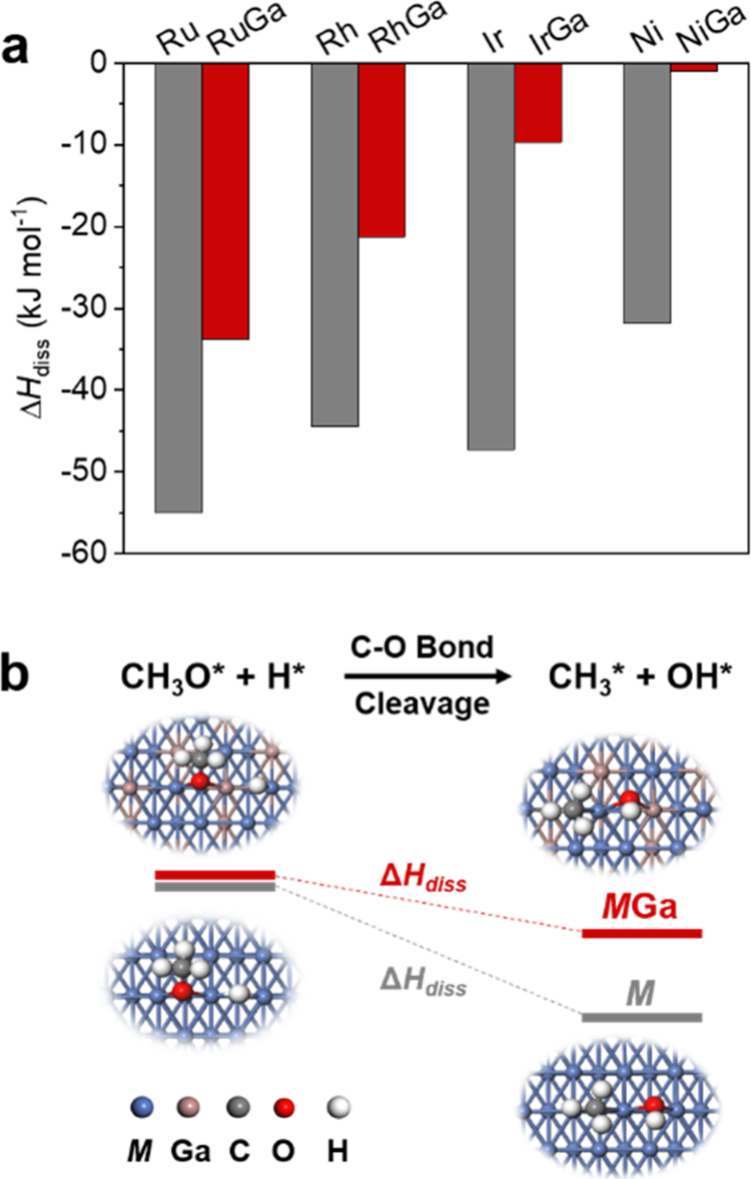
DFT calculation studies. (a) Dissociation enthalpies of C–O in methoxy on the representative facet of different monometallic *M* and bimetallic *M*Ga (*M* = Ru, Rh, Ir, and Ni) systems based on face-centered cubic (FCC) structure. (b) Scheme for cleaving the C–O bond of CH_3_O* in the presence of H* on monometallic *M* and bimetallic *M*Ga.

*In situ* DRIFTS indicates the presence of methoxy species during the reaction over bimetallic *M*Ga@SiO_2_ catalysts while mostly methane is detected for *M*@SiO_2_. We evaluate the propensity of C–O bond cleavage in an adsorbed methoxy species in the presence of H adatoms on various metal surfaces using a representative facet based on an FCC structure as a simple descriptor (Figure S88). Strongly negative dissociation enthalpies (Δ*H*_diss_) hint toward facilitated C–O bond cleavage, while less negative values indicate a higher activation barrier for C–O cleavage and therefore higher probability for methanol formation. As shown in Figures 4, S89–S96, and Table S11, the dissociation energy of C–O on monometallic systems is substantially more negative than that in *M*Ga systems, suggesting that the cleavage of C–O yielding CH_3_* species (which can be readily converted to CH_4_) is more favorable on monometallic systems. In contrast, the presence of Ga in bimetallic *M*Ga alloys significantly weakens the capacity of C–O cleavage, thus stabilizing the methoxy species and promoting methanol formation. Combined with the *in situ* studies, we propose that the retention of the *M*Ga alloy during the CO_2_ hydrogenation is key to suppressing the methanation reaction while stabilizing the methoxy species and consequently promoting the formation of methanol.

## Conclusions

This work shows that Ga provides a unique promotional effect by switching the reactivity of classical methanation catalysts, here group 8 and 9 noble metal −Ru/Os and Rh/Ir–silica-supported nanoparticles, to favor methanol synthesis under classical CO_2_ hydrogenation conditions. The addition of Ga significantly decreases the rate of methanation in favor of the formation of oxygenates, in particular, methanol and DME. *In situ* XAS studies indicate that the nonpromoted monometallic *M* systems remain fully metallic under CO_2_ hydrogenation conditions and therefore present high activity for the methanation reaction. In contrast, the introduction of Ga enables us to generate stable bulk *M*Ga alloys under CO_2_ hydrogenation conditions, suppressing methanation. Yet, *in situ* DRIFTS experiments evidence the formation of *M*Ga–GaO_*x*_ interfaces that are likely critical for stabilizing methoxy species and promoting methanol formation.

Noteworthily, Ga is able to promote methanol formation across a range of metal and catalyst families: (i) the methanation catalysts based on Ru, Rh, Os, and Ir described here but also Ni as previously reported, and (ii) RWGS catalysts reported earlier based on Cu, Pd, and Pt. For the latter family, both methanol selectivity and catalytic activity are increased by the introduction of Ga, while for the former family, methanol selectivity is promoted at the expense of catalytic activity while suppressing methanation. The change of catalytic properties coincides with the formation of *M*Ga alloy upon H_2_ treatment in all cases, but the extent of dealloying during CO_2_ hydrogenation varies across systems. For the RWGS systems, the extent of dealloying varies from full dealloying for CuGa yielding Cu(0)/GaO_*x*_ interfaces to partial and slight dealloying for PdGa and PtGa, respectively; in these cases, alloying during synthesis is likely important to maximize *M*Ga–GaO_*x*_ and/or *M*–GaO_*x*_ interfaces under CO_2_ hydrogenation conditions needed for methanol formation. For methanation catalysts studied here as well as Ni, both the persistence of an alloy and the formation of a small fraction of *M*Ga–GaO_*x*_ interfaces are key to suppressing methanation and driving selective methanol formation. These observations highlight how Ga is a universal promoter that changes the state and reactivity of transition-metal elements and provide general guideline principles to develop methanol synthesis catalysts.

## Supporting Information Available

Details for catalysts synthesis; crystal structures; routine characterization; DFT calculations; and catalytic tests have been included in the Supporting Information (PDF)
