# Supplementary material for: Gallium: A Universal Promoter Switching CO2 Methanation Catalysts to Produce Methanol
Source: JACS Au. 2024 Dec 20;5(1):217–24. doi: 10.1021/jacsau.4c00893 (PMC11775694; doi:10.1021/jacsau.4c00893)
Supplement: Supplementary file 1 — au4c00893_si_001.pdf [file au4c00893_si_001.pdf]

Supporting information

Gallium: A Universal Promoter Switching CO_2 methanation Catalysts to Produce Methanol

Wei Zhou,[a‡] Colin Hansen,[a‡] Weicheng Cao,[a] Enzo Brack,[a] Scott R. Docherty,[a] Christian Ehinger,[a] Yuhao Wang,[b] Chunliang Wang,[b] and Christophe Copéret[a]*

[a]Department of Chemistry and Applied Biosciences, ETH Zürich, CH-8093 Zurich, Switzerland

[b]Engineering Research Center of Metallurgical Energy Conservation and Emission Reduction, Ministry of Education, Kunming University of Science and Technology, Kunming 650093, China

[‡]These authors contributed equally to this work

*Corresponding author: ccoperet@inorg.chem.ethz.ch

Experimental and details

General procedure

Unless otherwise stated, all operations of precursor synthesis and catalysts preparation were performed under an argon atmosphere in an MBraun glove box or using standard Schlenk techniques. After preparation all materials were stored in an argon filled glovebox. *Celite*® and Molecular Sieves (4 Å, Merck) were activated under high vacuum (10^{-5} mbar) overnight at 350 ^{o}C. Deionized water was collected from Merck Millipore Synergy® Water Purification System. Anhydrous tetrahydrofuran (THF) was dried over Na^0/benzophenone and subsequently distilled. Dry deuterated Benzene (C_6D_6) was obtained via vacuum distillation from purple Na^0/benzophenone. Dry deuterated Chloroform ($CDCl_3$) was obtained via vacuum distillation from CaH_2. Dichloromethane (CH_2Cl_2), Pentane, Toluene (C_7H_8) and diethyl ether were dried using an MBraun SPS 800 solvent purification system where columns used for purification were packed with activated copper and alumina. Anhydrous dioxane ($C_4H_8O_2$) was vacuum transferred from purple Na^0/benzophenone. Hexamethyldisiloxane (HMDSO) was vacuum transferred from CaH_2. All solvents were further degassed via three freeze-pump-thaw cycles and stored over 4 Å molecular sieves after being transferred to a glove box. $NaOSi(O^tBu)_3$ was prepared according to literature procedures.[1, 2] Ferrocene was purchased from Sigma Aldrich and sublimed under reduced pressure prior to use. Chloro(1,5-cyclooctadiene)iridium(I) dimer (Sigma-Aldrich, 97%), Dichloro(p-cymene)ruthenium(II) dimer (abcr GmbH, 98%), $OsCl_3{\cdot}3H_2O$ (Sigma-Aldrich, 99.90%), α-Phellandrene (Sigma-Aldrich, ≥ 85%) and anhydrous ethanol (Thermo Scientific-ACR, 99.5%) were used as received. Lithum (N,N'-diisopropylacetamidinate)(Tetrahydrofuran) (Li(DIA)(THF)) and (N,N'-diisopropylacetamidinate)(1,5-cyclooctadiene)rhodium (Rh(COD)(DIA)) were synthesized according to the literature procedure and stored in glovebox (Argon).[3]

Synthesis of Ir(COD)(DIA)

In an Ar filled glovebox, Chloro(1,5-cyclooctadiene)iridium(I) dimer (360 mg, 0.536 mmol, 0.500 equiv) and LiDIA(THF) (239 mg, 1.09 mmol, 1.01 equiv) were transferred to a Schlenk flask equipped with a stir bar. Pentane (20 mL) was added, and the mixture was stirred for 4 days at room temperature. The precipitated LiCl was removed by filtration over celite, and the yellow-orange filtrate was concentrated to dryness yielding the crude product as an orange solid. Further purification was achieved by recrystallization from a concentrated

pentane solution at -30 °C, affording the product as orange crystals. Combined yield from 3 crops: 416 mg (88% yield). **^{1}H NMR** (300 MHz, C_6D_6) δ 4.35-4.2 (m, 4H), 3.39 (hept, J = 6.4 Hz, 2H), 2.35-2.15 (m, 4H), 1.55-1.35 (m, 4H), 1.21 (s, 3H), 0.93 (d, J = 6.4 Hz, 12H). **^{13}C NMR** (126 MHz, C_6D_6) δ 183.2, 59.1, 48.5, 32.6, 24.7, 12.6. **IR** (ATR, cm^{-1}): 2983, 2955, 2921, 2886, 2821, 1467, 1452, 1368, 1354, 1339, 1313, 1225, 1169, 1138, 1119, 1062, 1007, 989, 951. **Elemental Analysis**: calculated for $C_{16}H_{29}N_2Ir$: C 43.51%, H 6.62%, N 6.34%; found: C 43.52%, H 6.78%, N 6.31%.

Synthesis of [(*p*-Cymene)Ru(OSi(O^{t}Bu)$_3$)$_2$]

To a solid mixture of Dichloro(p-cymene)ruthenium(II) dimer ([*p*-Cymene)$RuCl_2$]$_2$) (403.2 mg, 0.66 mmol) and NaOSi(O^{t}Bu)$_3$ (759.4 mg, 2.61 mmol), THF (15 mL) was added, and the resulting mixture was stirred for 72 h at room temperature. All volatile materials were removed under reduced pressure, giving a red/brown solid. Pentane (15 mL) was added and the resulting solution was filtered through celite. The solution was concentrated and cooled to -35°C overnight to yield 787 mg of dark red/brown crystals (79%). **^{1}H-NMR** (300 MHz, C_6D_6): δ 6.07 (d, J = 6.0 Hz, 2H), 5.85 (d, J = 6.0 Hz, 2H), 2.45 (hept., J = 7.0 Hz, 1H), 1.68 (s, 3H), 1.52 (s, 54H), 1.25 (d, J = 7.0 Hz, 6H). **^{13}C-NMR** (75.5 MHz, C_6D_6): δ 88.9, 80.1, 78.7, 78.4, 71.4, 32.1, 31.3, 22.7, 18.6. **IR** (ATR, cm^{-1}): 3078, 2966, 2927, 1471, 1385, 1361, 1236, 1214, 1191, 1036, 1014, 987, 940, 869, 822, 796, 704, 694, 657, 518. **Elemental Analysis**: calculated for $C_{34}H_{68}O_8Si_2Ru$: C 53.6%, H 9.0%, Ru 13.3%; found: C 53.8%, H 9.0%, Ru 13.7%.

Synthesis of [(*p*-Cymene)$OsCl_2$]$_2$

The Synthesis was adapted from literature procedure.[4] To an Ar-filled Schlenk flask was added $OsCl_3 \cdot 3H_2O$ (950 mg, 2.71 mmol, 1.0 equiv.) in 40 mL of dry ethanol. Then 4.7 mL of α-Phellandrene was added dropwise. The reaction mixture was heated and stirred at reflux for 8 h, before cooling to room temperature and stirring for 12 h. During this time a fine yellow precipitate crashed out of solution. The supernatant was filtered off and the precipitate was washed with diethyl ether (3 × 5 mL) before drying the product under reduced pressure. The procedure yielded a yellow powder in a yield of 500 mg (47 %). The complex was used without further purification (as impurities indicated by ^{1}H-NMR were negligible). **^{1}H-NMR** (300 MHz, C_6D_6): δ 6.08 (d, J = 5.6 Hz, 2H), 5.92 (d, J = 5.6 Hz, 2H), 2.68 (hept., J = 6.9 Hz, 1H), 2.11 (s, 3H), 1.19 (d, J = 6.9 Hz, 6H).

Synthesis of [(*p*-Cymene)Os(OSi(O^{t}Bu)$_3$)$_2$]

In an Ar filled glovebox, [(*p*-Cymene)OsCl$_2$]$_2$ (100 mg, 0.126 mmol, 1.0 equiv. Os) and NaOSi(O^{t}Bu)$_3$ (158 mg, 0.552 mmol, 2.2 equiv.) were weighed in a Schlenk flask. THF (10 mL) was added, and the mixture was stirred for 12 hours at room temperature. The precipitated NaCl was removed from the red/brown solution by filtration over celite. The filtrate was concentrated to dryness yielding a crude red/brown powder. The crude product was purified by crystallization from pentane (ca. 10 mL) at -40 °C. After collecting the first crop, the mother liquor was concentrated further and cooled to -40 °C again. Repeating this procedure afforded the title compound as a red/brown crystalline solid. Combined yield of two crops: 151 mg (70%). **^{1}H-NMR** (500 MHz, C_6D_6): δ 7.07 (d, J = 6.0 Hz, 2H), 6.85 (d, J = 6.0 Hz, 2H), 2.45 (hept., J = 6.9 Hz, 1H), 1.90 (s, 3H), 1.50 (s, 54H), 1.35 (d, J = 6.9 Hz, 6H). **^{13}C-NMR** (75.5 MHz, C_6D_6): δ 82.6, 72.3, 71.4, 70.9, 69.0, 33.5, 31.8, 23.1, 21.4. **IR** (ATR, cm^{-1}): 2966, 2926, 2902, 2869, 1469, 1386, 1360, 1239, 1212, 1190, 1037, 1016, 982, 932. **Elemental Analysis**: calculated based on $Os_1Si_2O_8C_{34}H_{68}$: Os 22.35%, C 47.97%, H 8.05%; found: Os 21.6%, C 48.09%, H 8.10%.

Preparation of $SiO_{2\text{-}700}$

Silicon dioxide (Aerosil Degussa 200) partially dehydroxylated at 700 °C ($SiO_{2\text{-}700}$) was prepared according to literature procedure.[5] The preparation of the $SiO_{2\text{-}700}$ support is initiated via a calcination under static air at 500 °C for 12 h. Dehydroxylation under high-vacuum (10^{-5} mbar) at 700 °C for 24 h was then undertaken. The temperature profile is reported below. Quantification of the –OH density of the $SiO_{2\text{-}700}$ was performed through reaction of [Mg(CH$_2$Ph)$_2$(THF)$_2$] with a known amount of the $SiO_{2\text{-}700}$. The amount of toluene liberated was quantified by NMR in C_6D_6 using ferrocene as an internal standard. For this purpose, a recycle delay (D_1) of 58 seconds was used. The Si-OH density is titrated to a value of 0.30 mmol/g (0.9 -OH nm^{-2}) consistent with earlier reports.[5]

Preparation of Ga^{III}@SiO_2

To a suspension of $SiO_{2\text{-}700}$ (1.000 g, 0.300 mmol -OH) in C_6H_6 (10 mL) was added a clear solution of [Ga(OSi(O^{t}Bu)$_3$)$_3$(THF)] (273.8 mg, 0.295 mmol) in C_6H_6 (5 mL).[6] The suspension was stirred for 6 h (100 rpm) before stirring was halted, and the mixture allowed to react for 12 more hours at 25 °C. After the reaction, the material was washed with C_6H_6 (3 x 3 mL) and the washings were retained for quantification purposes

(Using ^{1}H-NMR spectroscopy, release of Isobutene (4.0 eq.), *tert*-Butanol (1.6 eq.) and THF (0.7 eq.) were observed). No starting material was observed in the washings. The material was dried under vacuum to remove all volatiles. The produced material was subjected to thermal treatment (150 °C (2 h), 300 °C (2 h), 400 °C (2 h) and 500 °C (10 h) at 10^{-5} mbar, ramp for all the steps: 5 °C/min) to remove all the ligand to obtain a light-gray $Ga^{III}@SiO_2$. **Elemental Analysis:** Ga 1.83 wt%.

Preparation of *M*@SiO$_{2\text{-}700}$ and *M*Ga@SiO$_2$ (*M* = Ru, Os, Rh and Ir)

The monometallic *M*@SiO$_2$ and Ga-doped *M*Ga@SiO$_2$ were prepared by grafting the corresponding molecular precursors. As an illustrative procedure, 115 mg of (*p*-Cymene)Ru(OSi(O^{t}Bu)$_3$)$_2$ (0.150 mmol) was dissolved in ca. 10 mL benzene and was stepwise added to a slurry of 0.5 g $SiO_{2\text{-}700}$ (0.15 mmol -OH) or $Ga^{III}@SiO_2$ in benzene (5 mL). The suspension was stirred at 100 rpm for 4 h. After the reaction, the material was washed with C_6H_6 (3 x 5 mL) and the washings were kept for quantification purposes (Using ^{1}H-NMR spectroscopy (200 MHz, 25°C, d1 = 58 s) with ferrocene as an internal standard). The material was dried in vacuum to remove all volatiles. The produced material was subjected to reduction conditions (400 °C for 5 h in a dynamic H_2 atmosphere of approx. 1 atm, ramp: 1 °C/min) to remove all organics to obtain a black powder Ru@SiO$_2$ or RuGa@SiO$_2$. The other three metals (Os, Rh and Ir), *M*@SiO$_2$ and Ga-doped *M*Ga@SiO$_2$ materials are prepared by the same procedure using 0.15 mmol of (*p*-Cymene)Os(OSi(O^{t}Bu)$_3$)$_2$, Rh(COD)(DIA) and Ir(COD)(DIA) instead of (*p*-Cymene)Ru(OSi(O^{t}Bu)$_3$)$_2$ to graft on 0.5 g $SiO_{2\text{-}700}$ or $Ga^{III}@SiO_2$. Note that RhGa@SiO$_2$ with different Rh/Ga ratios was controlled by varying the amount of Rh(COD)(DIA), namely using 0.15 mmol, 0.075 mmol and 0.0375 mmol to graft on 0.5 g $Ga^{III}@SiO_2$. **Elemental Analysis** of all the as-synthesized materials are summarized in Table S1.

Catalyst characterization

Fourier-Transform Infrared (FTIR) spectroscopy experiments were performed on self-supporting wafers using a Bruker Alpha FT-IR spectrometer in transmission mode (24 scans, 2 cm^{-1} resolution) under air-free conditions. CO-FTIR measurements were carried out on a Nicolet 6700 FTIR instrument. For this purpose, a self-supporting pellet of metal-based catalysts was exposed to a CO atmosphere at various pressures and evacuated (10^{-5} mbar) for 15 min at room temperature. The spectra were collected from 4000 to 600 cm^{-1} at a resolution of 2 cm^{-1} in transmission mode. Spectra are normalized to the Si-O-Si overtone peak maximum at 1868 cm^{-1} for all the materials.

In situ high-pressure diffuse reflectance infrared Fourier-transform spectroscopy (DRIFTS) measurements were carried out on an FTIR spectrometer (Thermo Fisher iS50) equipped with liquid nitrogen N_2 cooled mercury–cadmium–telluride (MCT) detector. The spectra were recorded from 4000 to 600 cm^{-1}. The catalyst powders were placed in a high-pressure (0–10 MPa) DRIFTS cell (Harrick) equipped with ZnSe windows. Prior to each measurement, the sample was pretreated in pure H_2 (20 mL min^{-1}) at 400 °C for 1 h. Then, the sample was cooled to 230 °C, followed by pressurizing to 20 bar with H_2/Ar (3:2). After recording the background spectrum, a CO_2/H_2/Ar (1:3:1) gas at 20 bar was switched into the *in situ* DRIFTS cell at 230 °C, and the evolution of IR spectra was recorded.

Solution NMR spectra were recorded at room temperature (25 °C) on a Bruker 500 MHz solution state spectrometer equipped with either a broadband probe or a broadband cryoprobe (^{13}C, ^{1}H). ^{1}H and ^{13}C chemical shifts are referenced relative to residual solvent peaks.[7] Chemical shifts are reported in parts per million (ppm). Where appropriate, signal multiplicity has been condensed to a single letter format, i.e.: s=singlet, d=doublet, t=triplet, q=quartet, m=multiplet. Observed J-couplings (J) are reported in Hz. Solvent signals are denoted accordingly. Unless otherwise specified, ^{13}C spectra were recorded using 2048 scans, and ^{1}H spectra were acquired using 64 scans.

Elemental Analysis was provided by the in-house Molecular and Biomolecular Analysis Service (MoBiAS) of ETH Zürich and Analytisches Labor Pascher in Remagen, Germany.

Catalyst morphologies were assessed by transmission electron microscopy (TEM) on a JEOL JEM F200 (200 kV) and JEOL ARM 200F (300 kV) microscope within the facilities of ScopeM at ETH Zurich. The prepared catalysts were dry casted as solid onto UC on Lacey carbon 400 mesh Cu grids (Ted Pella) in an Argon glovebox and mounted on a vacuum transfer holder (Gatan). The particle size distribution was determined by counting greater than 100 individual particles, and the average size and standard deviation are determined by using a normal distribution function.

In-situ X-ray adsorption spectroscopy (XAS) experiments were measured at BM31 of the Swiss-Norwegian Beamlines (SNBL) located at the European Synchrotron Radiation Facility (ESRF) in Grenoble, France. Ru K-edge, Rh K-edge, Ir L_3-edge and Ga K-edge were collected in transmission mode using a double crystal Si (111) monochromator. In each case, the ionization chambers were filled with a gas mixture optimized for the edge energy and path length required. For each edge, a secondary reference for energy calibration was used (Rh-foil, Ir-foil, Ru-foil, and $Ga(acac)_3$). Typical beam dimensions used were 0.4 mm (H) x 4 mm (W), and the beam size was controlled using slits. The sample was packed into quartz capillary (1.5 mm (0.02 mm wall thickness)), in each case, a catalyst bed of ca. 1 cm in length was used, and the powdered sample was secured using quartz wool plugs at each end. The packed capillary was loaded into a cell for *in situ* XAS experiment. For acquisition of EXAFS, spectra were collected at beam energies ranging from 22.0-23.1 keV, 23.1-24.1 keV, 11.12-12.22 keV and 10.27-11.37 keV for the Ru K, Rh K, Ir L_3 and Ga K, respectively. 10 scans for Ru K-edge, 5 scans for Rh K-edge, 5 scans for Ir L_3-edge and 3 scans for Ga K-edge were averaged to obtain a sufficient quality for the structural analysis. The acquisition time for XANES and EXAFS at Ru K-edge, Rh K-edge, Ir L_3-edge, and Ga K-edge were ca. 1 and 2.2 minutes, 0.9 and 2.2 minutes, 1.2 and 3.2 minutes, and 1.2 and 2.2 minutes, respectively. Flow rates (Ar, H_2, CO_2) were controlled using mass-flow controllers (Bronkhorst), and the pressure was set at 20 bar using a back pressure regulator (Bronkhorst, EL-PRESS). Throughout the experiments, a flow of 10 mL min^{-1} was maintained, and the outlet gas was monitored using a mass spectrometer to ensure that similar chemistry proceeds over catalysts as under reaction conditions. After pressurization, the baseline shift, associated with the introduction/removal of a fraction of Ar was used to assess when composition of feed gas evolved at the catalyst bed, using a strategy similar to that described by Lomachenko et al.[8] The temperature for H_2 reduction (400 °C) and CO_2 hydrogenation (230 °C) were maintained using a SiC heating element with a K-type thermocouple mounted inside. In a typical experiment, EXAFS spectra were first collected after the sample was mounted and exposed to air. Subsequently, H_2 pretreatment was carried out under 10 mL min^{-1} of H_2 for 1 h at 400 °C (300 °C h^{-1} ramp). After cooling to room temperature (or < 50 °C) under a flow of H_2, EXAFS spectra were collected for the reduced sample. Then, heating to reaction temperature (230 °C) under a flow of 10 mL min^{-1} of H_2/Ar (3:2) mixture (300 ºC h^{-1} ramp), followed by pressurizing to 20 bar and retaining this condition for 20 min. Subsequently, the gas composition was switched to CO_2/H_2/Ar (1:3:1) mixture for CO_2 hydrogenation and the condition was retained for 2 h. After CO_2 hydrogenation, the gas was switched to Ar and the sample was cooled to room temperature

for the post-CO_2-hydrogenation EXAFS spectra. Demeter software (0.9.24) from the Ifeffit software package (Version 1.2.11) was used for the XAS data analysis.[9]

The Small Molecule Crystallography Center (SMoCC) of ETH Zürich provided the equipment for single crystal structure determination. XRD data was recorded on either a Rigaku XtaLAB Synergy-S single crystal diffractometer or a Bruker APEX-II CCD diffractometer. Suitable crystals were selected and tip-mounted on a MiTeGen Pin covered with Paratone Oil. Using Olex2,[10] the structure was solved with the SHELXT[11] structure solution program and refined with the SHELXL[12] refinement package using CGLS minimization.

Density functional theory (DFT) calculations

All the calculations were performed using the Vienna ab initio simulation package (VASP) and a kinetic energy cutoff of 600 eV.[13] The generalized gradient approximation (GGA) was used with the Perdew-Burke-Ernzerhof (PBE) functional.[14, 15] The optimization thresholds were 10^{-6} eV and 0.01 eV/Å for electronic and ionic relaxations, respectively. All the bulk structures were optimized using a 8 × 8 × 8 Monkhorst–Pack k-points grid mesh. All the surface slab structures were optimized using a 5 × 5 × 1 Monkhorst–Pack k-points grid mesh.

Bulk structure and alloy formation. To evaluate the affinity of the metal under study with Ga, the alloy formation enthalpies of the $M_{0.75}Ga_{0.25}$ (M = Ru, Rh, Ir and Ni) were calculated. The alloy formation enthalpy change ($\Delta H_{alloying}$) is defined as:

$$\Delta H_{alloying} = E_{Bulk}(M_{0.75}Ga_{0.25}) - 0.75 * E_{Bulk}(M) - 0.25 * E_{Bulk}(Ga)$$

where $E_{Bulk}(M_{0.75}Ga_{0.25})$ is the normalized (per atom) energy of bulk face-centered cubic (FCC) $M_{0.75}Ga_{0.25}$ alloy structure, $E_{Bulk}(M)$ denotes the normalized (per atom) energy of bulk FCC monometallic structure, and $E_{Bulk}(Ga)$ stands for the normalized (per atom) energy of bulk orthorhombic Ga structure. To be noted, the most stable phase for different elements was chosen respectively for the calculation of bulk models. FCC structure was chosen for modeling the pure metal M and alloy MGa systems while the orthorhombic structure was chosen for the pure Ga system. The bulk $M_{0.75}Ga_{0.25}$ alloy models were constructed through the substitution of Ga in the FCC pure bulk M models, specifically by replacing a portion of the M atoms with Ga. The ratio of M to Ga is 3 to 1, indicating that the fraction of M is 0.75 and the fraction of Ga is 0.25 in the bulk model. Besides, two different atomic arrangements for the bulk alloy models has been considered, as shown in

Supplementary Figure 83 - 86. The details of the models and the calculated results are summarized in Table S10 and plotted in Figure 87.

Surface models of *M* and *M*Ga systems. Based on in situ DRIFTS experiments, the reaction enthalpy change for the key reaction step $CH_3O^* + H^* \leftrightarrow CH_3^* + OH^*$ (methoxy dissociation reaction with the presence of absorbed H*) was calculated to serve as a descriptor for the capacity of C-O bond cleavage over both pure metal *M* and alloy *M*Ga surfaces. The details are describe as the following: The pure $M\{211\}$ surface slab models contain four layers of metal in the [111] direction were constructed based on the 2 × 2× 2 supercell of the optimized pure *M* bulk structures. To avoid interactions between slabs, all slabs were separated by a vacuum gap of 15 Å. The *M*Ga {211} surface slab models were constructed based on the optimized $M\{211\}$ models by replacing a portion (~27 %) of the *M* atoms with Ga to ensure the symmetry of modelling. All the atoms were fully relaxed under the optimization. In addition, the structures of surface species $CH_3O^* + H^*$ on $M\{211\}$ and *M*Ga{211}, as well as $CH_3^* + OH^*$ on $M\{211\}$ and *M*Ga{211}, were optimized, as illustrated in Supplementary Figure 89-96. This reaction enthalpy change is denoted as ΔH_{diss} and calculated by :

$$\Delta H_{diss} = E({CH_3}^* + OH^*) - E(CH_3O^* + H^*)$$

Where the $E({CH_3}^* + OH^*)$ is the calculated energy of the optimized structure for CH_3^*+OH* on metal surfaces and $E(CH_3O^* + H^*)$ is the calculated energy of the optimized structure for CH_3O^*+H* on the corresponding surfaces. The summary of the calculated results is shown in Table S11.

Catalyst evaluation

CO_2 hydrogenation reactions were carried out on a fixed-bed flow reactor (PID Eng&Tech). Typically, 200 mg of catalyst (20 mg for Ru@SiO_2 and 30 mg for Rh@SiO_2) was mixed with 4.0 g of SiC and packed in the reactor in air. Prior to the CO_2 hydrogenation, the catalyst was reduced at 1 bar under 50 mL min^{-1} of H_2 for 1 h at 400 ^oC (10 $^oC/min$ ramp). After reduction, the furnace was cooled down to the reaction temperature (230 ^oC), and the reactor pressurized to the reaction pressure (40 bar) under the reacting gas flow (80 mL min^{-1} of 1:3:1 CO_2:H_2:Ar). The effect of conversion on product formation rates was probed by systematically varying the total gas flow rate from 6 mL min^{-1} to 100 mL min^{-1} (the details about how the flow rate was varied are shown in the following scheme). Finally, activity data was collected again at the initial flow rate to check for potential catalyst deactivation. The effluent gases were analyzed via online gas chromatography (Agilent 7890B equipped with Restek Rt-U-BOND (30 m x 0.53 mm x 20 µm) and Rt-Msieve 5A (30 m x 0.53 mm x

50 μm) columns) and quantified by a flame ionization detector (FID) for CH_3OH, C_2H_5OH and C_{2+} hydrocarbons) and thermal conductivity detector (TCD) for Ar, CO_2, CO and CH_4. GC data was collected in increments of half an hour.

The CO_2 conversion and product selectivity were calculated using the following set of equations:

$$S_x = \frac{F_{X,out}}{\sum_{i=1}^{n} F_{i,out}}$$

$$X_{CO2} = \frac{\sum_{i=1}^{n} F_{i,out}}{F_{CO2,in}}$$

Product selectivity (S_x) is defined as the outflow of the component in equation, $\sum_{i=1}^{n} F_{i,out}$, divided by the sum of of outlet flows for all carbon containing products, $\sum_{i=1}^{n} F_{i,out}$. The CO_2 conversion (X_{CO2}) is defined as the sum of outlet flows for all the carbon containing products, $\sum_{i=1}^{n} F_{i,out}$, divided by the inlet flow of CO_2, $F_{CO2,in}$. Note that all concentrations are normalized per carbon to enable accurate comparison.

The scheme for details of flow rate *vs* time on stream:

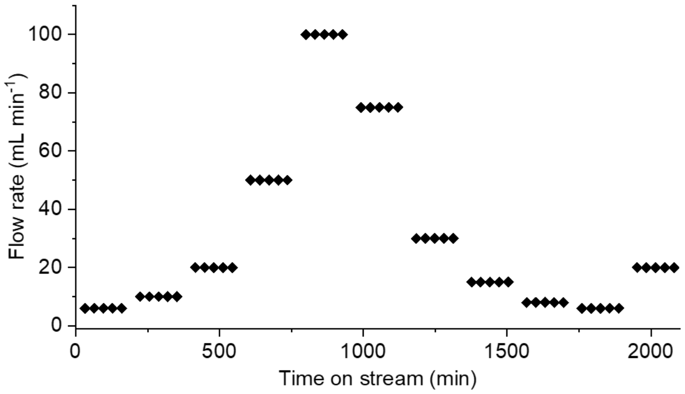

Supporting figures and tables

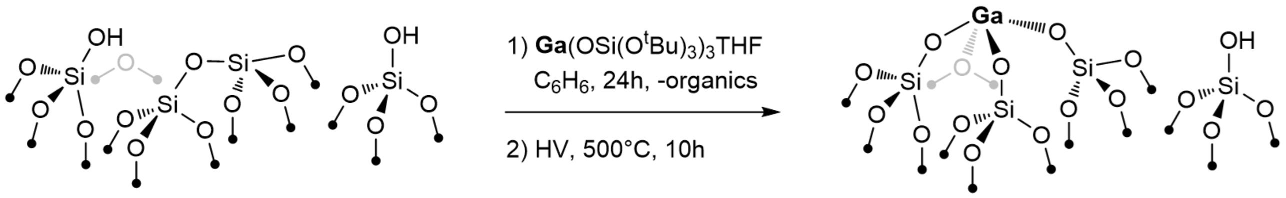

Figure S1. Schematic procedure for grafting of Ga(OSi(O^{t}Bu)$_3$)$_3$THF on SiO_{2-700} followed by thermal treatment under vacuum.

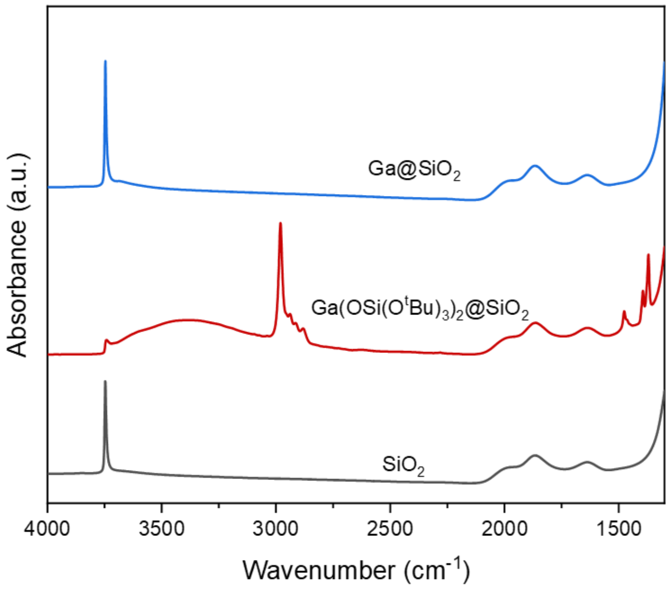

Figure S2. IR spectra throughout the synthesis of Ga@SiO_2.

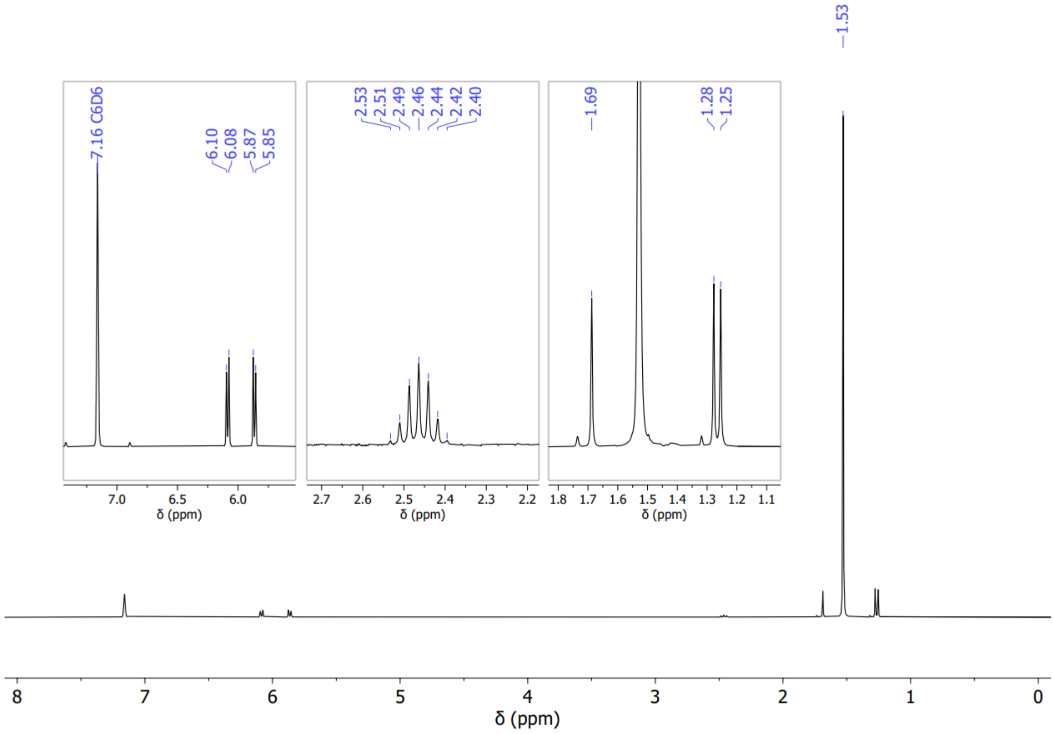

Figure S3. ^{1}H-NMR spectrum of (*p*-Cymene)Ru(OSi(O^{t}Bu)$_3$)$_2$. The spectra were recorded in C_6D_6 (300 MHz, 25°C).

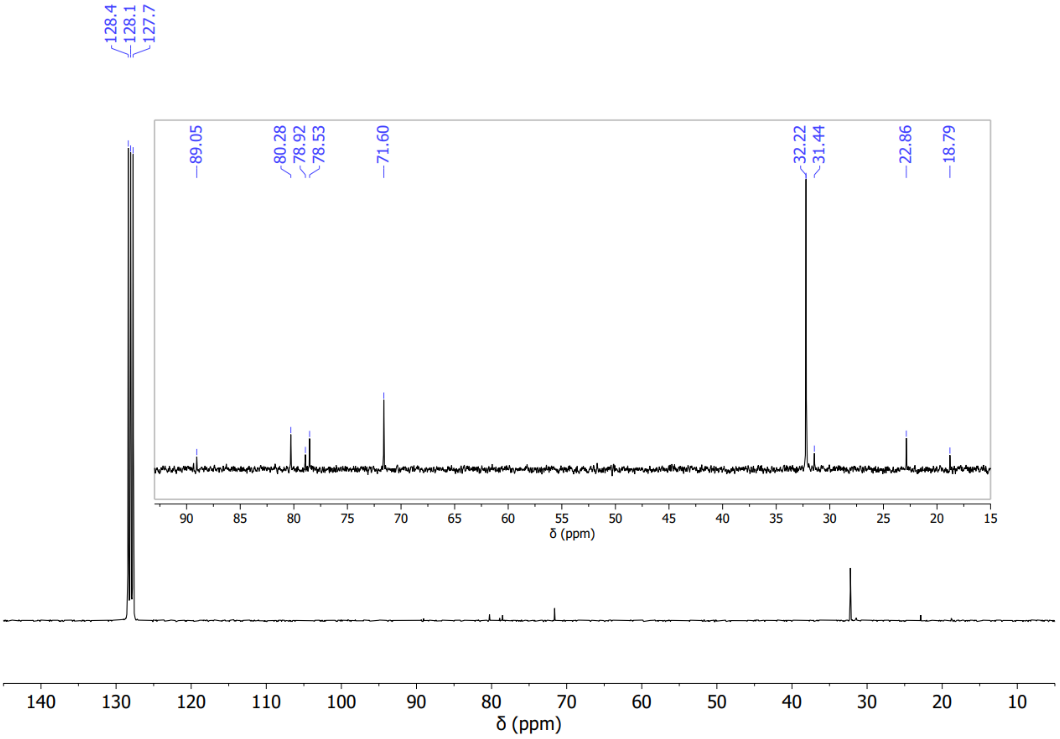

Figure S4. ^{13}C-NMR spectrum of (*p*-Cymene)Ru(OSi(O^{t}Bu)$_3$)$_2$. The spectra were recorded in C_6D_6 (75 MHz, 25°C).

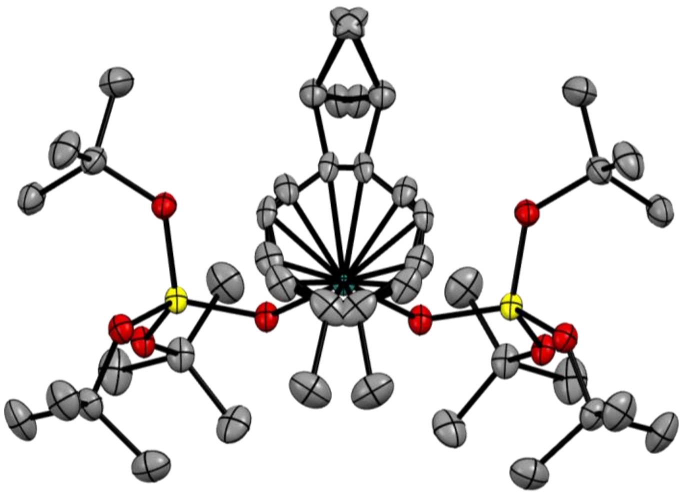

Figure S5. Crystal structure for (*p*-Cymene)Ru(OSi(O^{t}Bu)$_3$)$_2$. Ellipsoids shown at 50% probability, hydrogens omitted for clarity.

Single crystals of $C_{34}H_{64}O_8RuSi_2$ (**(*p*-Cymene)Ru(OSi(O^{t}Bu)$_3$)$_2$**) were crystallized from Pentane at -40 °C overnight. A suitable crystal was selected and tip-mounted on a MiTeGen Pin covered with Paratone Oil on a XtaLAB Synergy, Dualflex, HyPix diffractometer. The crystal was kept at 100 K during data collection. Using Olex2, the structure was solved with the SHELXT structure solution program using Intrinsic Phasing and refined with the SHELXL refinement package using CGLS minimisation.

Crystal Data for $C_{34}H_{64}O_8RuSi_2$ **(*M* = 762.06 g/mol):** monoclinic, space group Cm, a = 10.0313(4) Å, b = 25.8289(11) Å, c = 9.2532(4) Å, α = 90°, β = 122.6986(19)°, γ = 90°, V = 2017.54(15) Å^3, Z = 2, T = 100.01 K, μ(Mo Kα) = 0.491 mm^{-1}, $\rho calc$ = 1.255 g/cm^3, 54084 reflections measured (3.154° ≤ 2Θ ≤ 65.282°), 7174 unique (R_{int} = 0.0426, R_{sigma} = 0.0319) which were used in all calculations. The final R_1 was 0.0241 (I > 2σ(I)) and wR_2 was 0.0529 (all data).

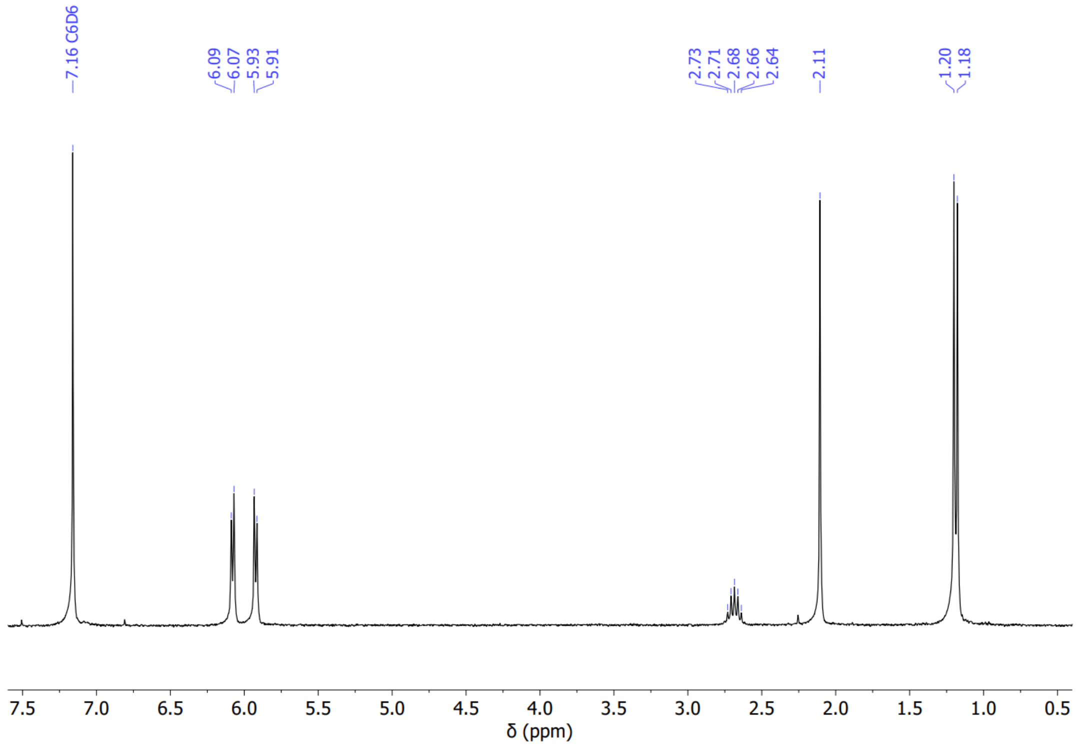

Figure S6. ^{1}H-NMR spectrum of [(*p*-Cymene)OsCl$_2$]$_2$. The spectra were recorded in C_6D_6 (300 MHz, 25°C).

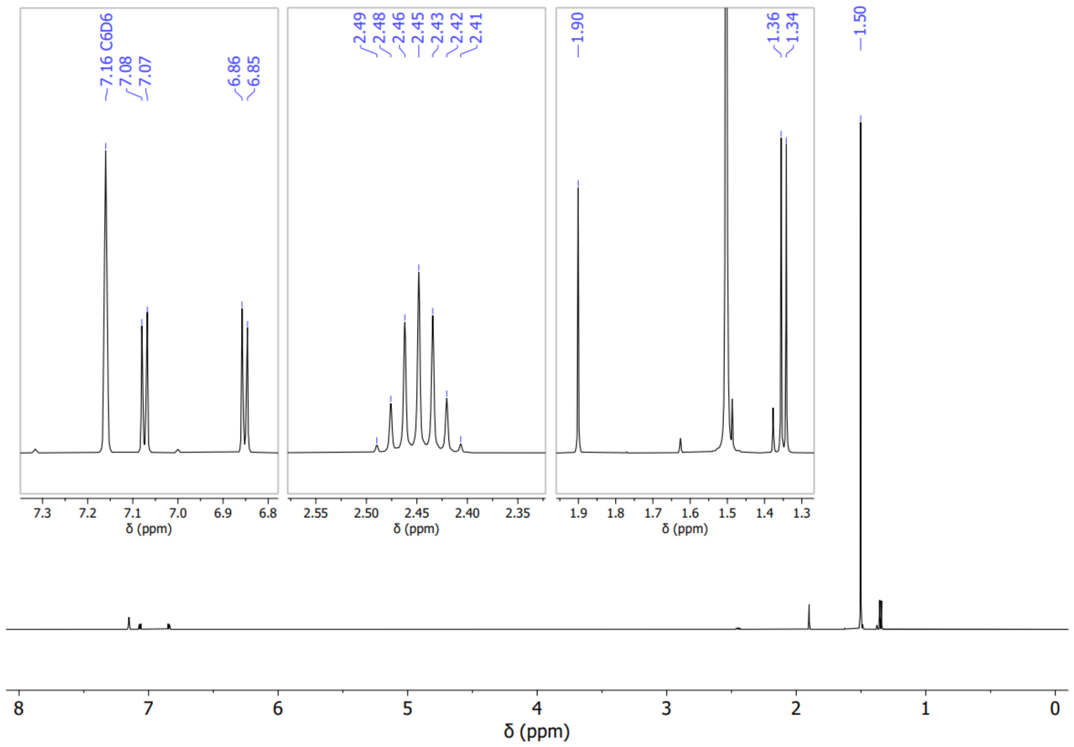

Figure S7. **^{1}H-NMR spectrum of (*p*-Cymene)Os(OSi(O^{t}Bu)$_3$)$_2$.** The spectra were recorded in C_6D_6 (500 MHz, 25°C).

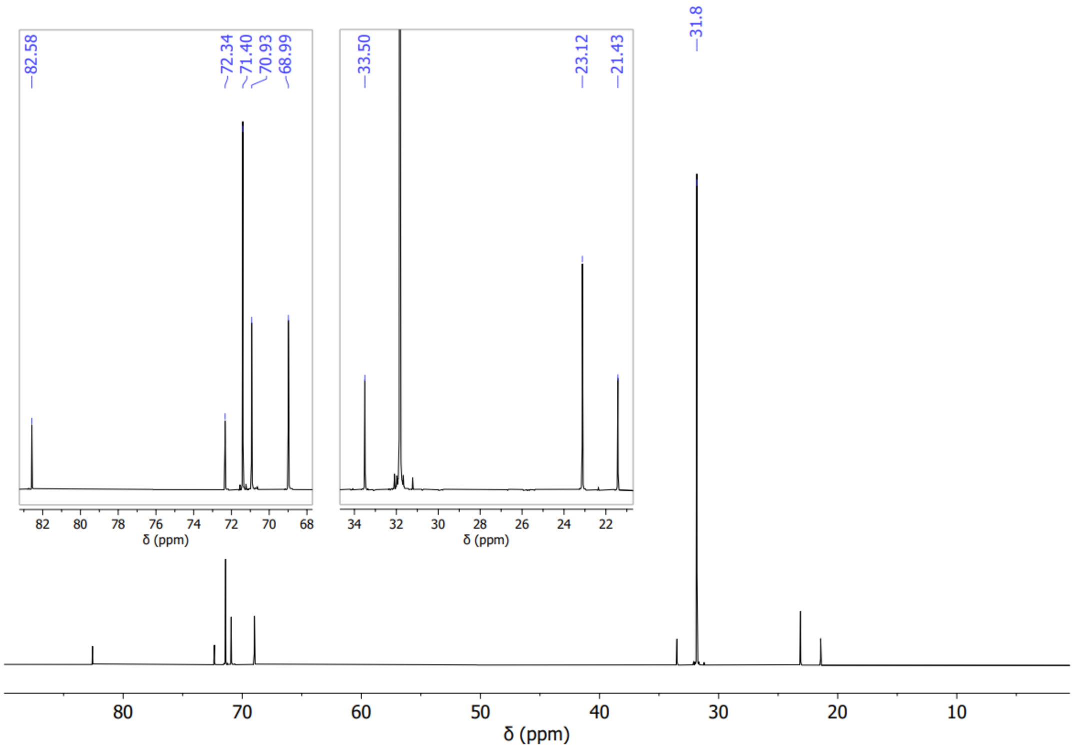

Figure S8. **^{13}C-NMR spectrum of (*p*-Cymene)Os(OSi(O^{t}Bu)$_3$)$_2$.** The spectra were recorded in C_6D_6 (125.7 MHz, 25°C).

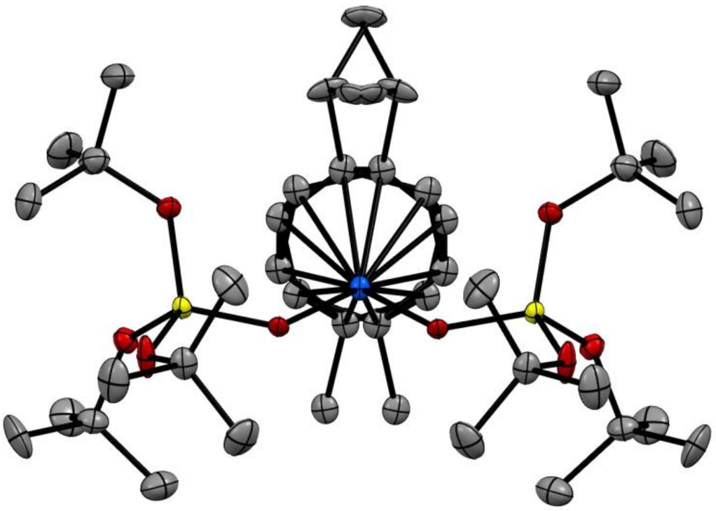

Figure S9. Crystal structure for [(*p*-Cymene)Os(OSi(O^{t}Bu)$_3$)$_2$]. Ellipsoids shown at 50% probability, hydrogens omitted for clarity.

Single crystals of $C_{34}H_{64}O_8OsSi_2$ (**(*p*-Cymene)Os(OSi(O^{t}Bu)$_3$)$_2$**) were crystallized from Pentane at -40 °C overnight. A suitable crystal was selected and tip-mounted on a MiTeGen Pin covered with Paratone Oil on a XtaLAB Synergy, Dualflex, HyPix diffractometer. The crystal was kept at 100 K during data collection. Using Olex2, the structure was solved with the SHELXT structure solution program using Intrinsic Phasing and refined with the SHELXL refinement package using CGLS minimisation.

Crystal Data for $C_{34}H_{64}O_8OsSi_2$ **(*M* = 847.23 g/mol):** monoclinic, space group Cm, a = 10.0656(2) Å, b = 25.6969 (3) Å, c = 9.27774(19) Å, α = 90°, β = 122.862(3)°, γ = 90°, V = 2015.72(8) Å^3, Z = 2, T = 100.01 K, μ(Mo Kα) = 3.265 mm^{-1}, $\rho calc$ = 1.396 g/cm^3, 45153 reflections measured (5.072° ≤ 2Θ ≤71.432°), 8769 unique (R_{int} = 0.0868, R_{sigma} = 0.0599) which were used in all calculations. The final R_1 was 0.0469 (I > 2σ(I)) and wR_2 was 0.1136 (all data).

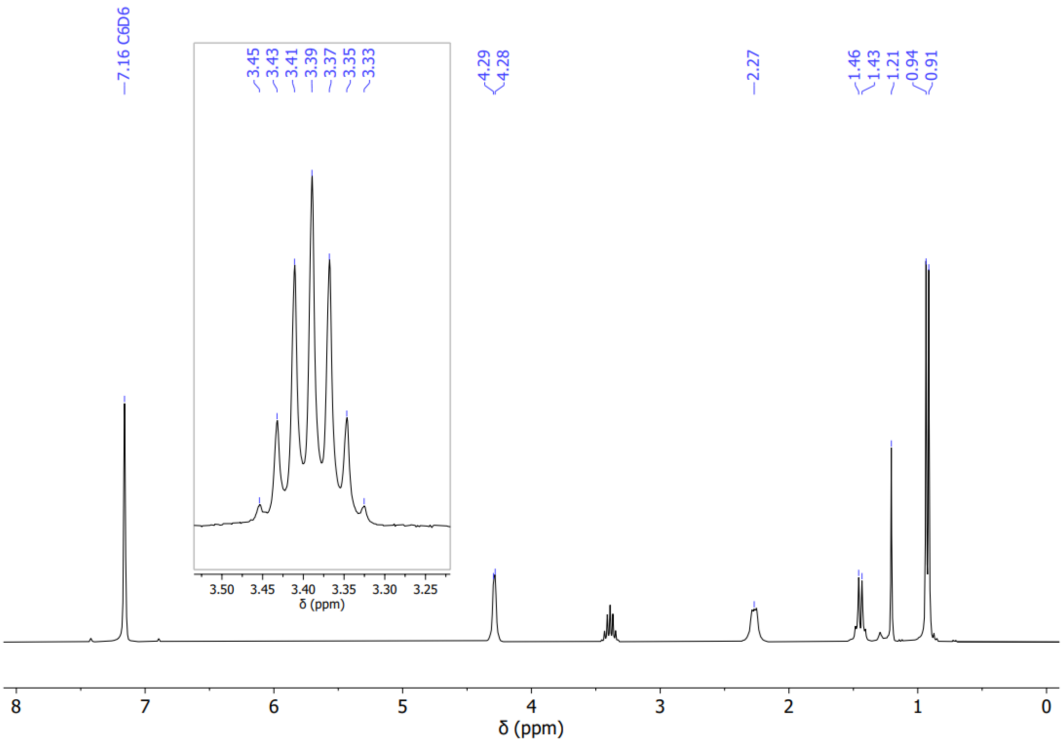

Figure S10. ^{1}H-NMR spectrum of Ir(COD)(DIA). The spectra were recorded in C_6D_6 (500 MHz, 25°C).

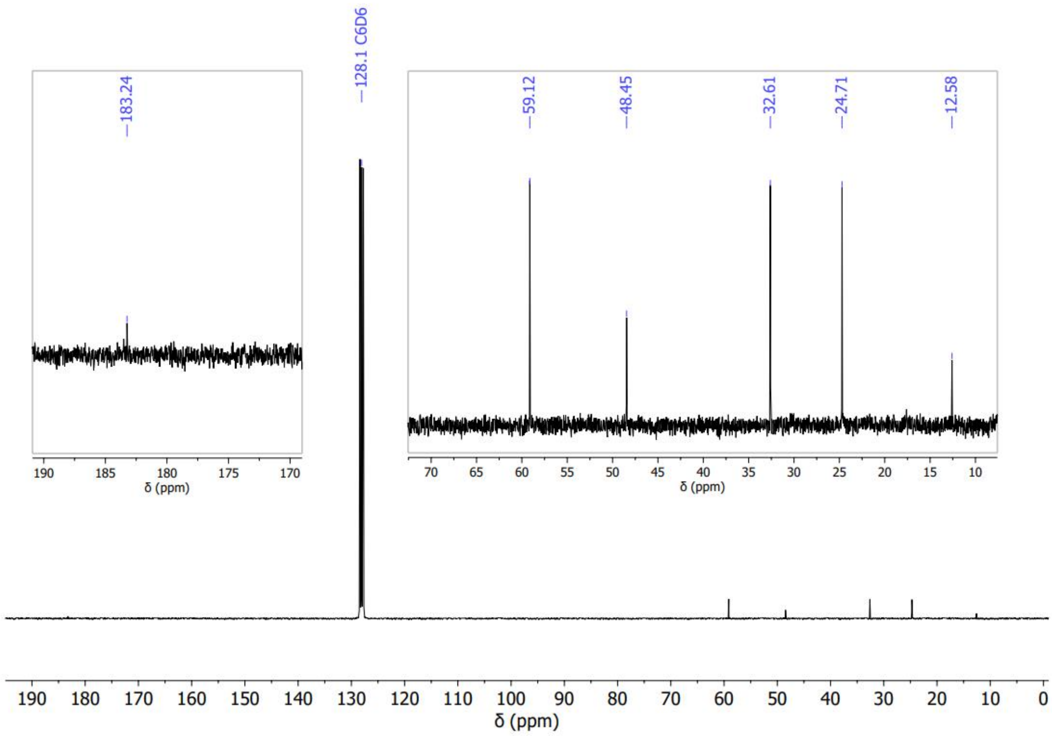

Figure S11. ^{13}C-NMR spectrum of Ir(COD)(DIA). The spectra were recorded in C_6D_6 (75 MHz, 25°C)

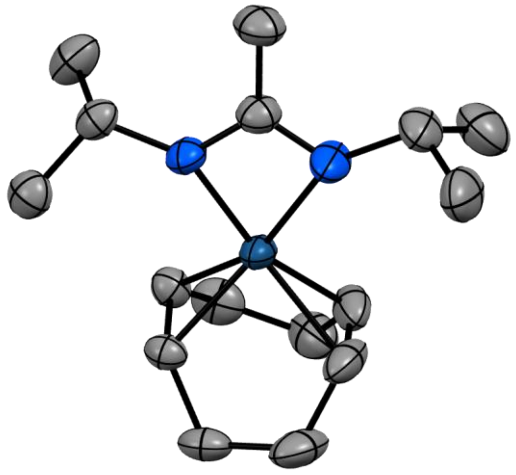

Figure S12. Crystal structure for Ir(COD)(DIA). Ellipsoids shown at 50% probability, hydrogens omitted for clarity.

Single crystals of $C_{16}H_{29}N_2Ir$ (**Ir(COD)(DIA)**) were crystallized from Pentane at -40 °C overnight. A suitable crystal was selected and tip-mounted on a MiTeGen Pin covered with Paratone Oil on a XtaLAB Synergy, Dualflex, HyPix diffractometer. The crystal was kept at 230 K during data collection. Using Olex2, the structure was solved with the SHELXT structure solution program using Intrinsic Phasing and refined with the SHELXL refinement package using CGLS minimisation.

Crystal Data for $C_{16}H_{29}N_2Ir$ **(*M* = 441.59 g/mol):** orthorhombic, space group Pbca, a = 10.6716(3) Å, b = 10.0985(2) Å, c = 30.3331(7) Å, α = 90°, β = 90°, γ = 90°, V = 3268.91(14) Å^3, Z = 8, T = 230 K, μ(Cu Kα) = 18.712 mm^{-1}, $\rho calc$ = 1.795 g/cm^3, 26037 reflections measured (5.828° ≤ 2Θ ≤ 160.184°), 3536 unique (R_{int} = 0.0307, R_{sigma} = 0.0175) which were used in all calculations. The final R_1 was 0.0297 (I > 2σ(I)) and wR_2 was 0.0718 (all data).

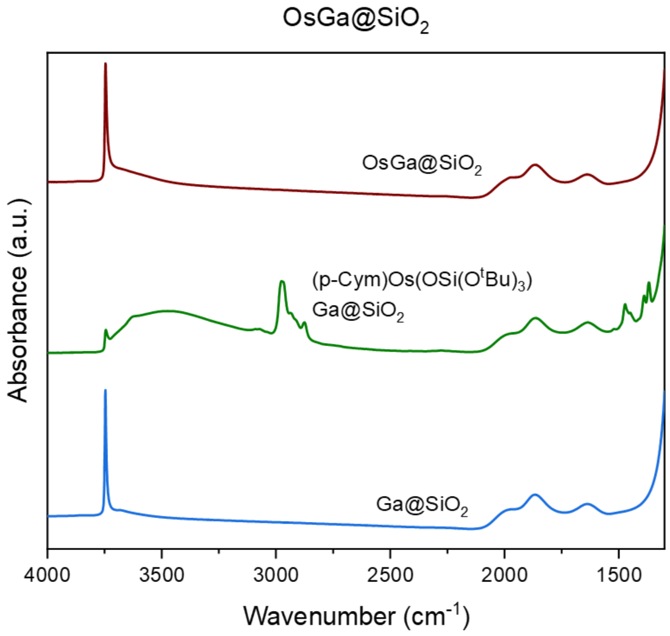

Figure S13. IR spectra throughout the synthesis of OsGa@SiO$_2$ starting from the second grafting.

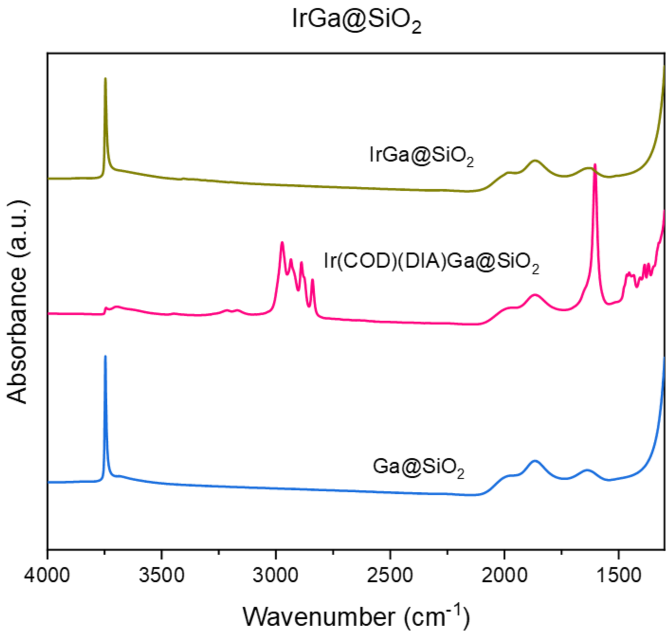

Figure S14. IR spectra throughout the synthesis of IrGa@SiO$_2$ starting from the second grafting.

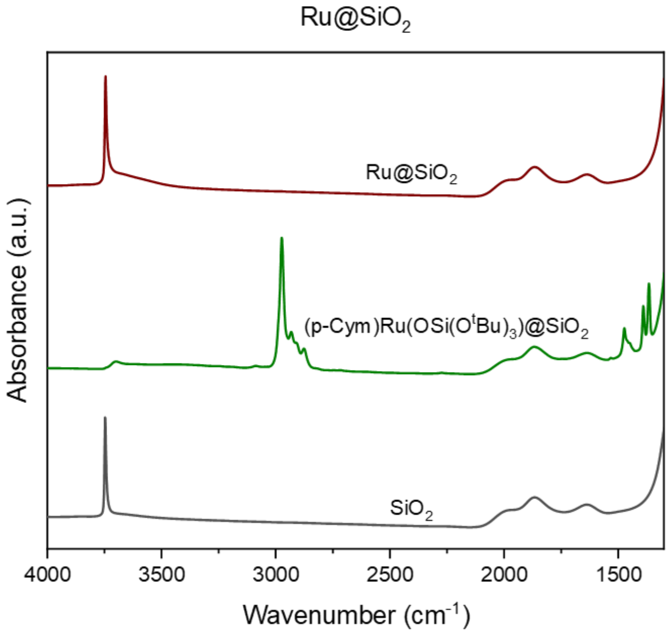

Figure S15. IR spectra throughout the synthesis of Ru@SiO₂.

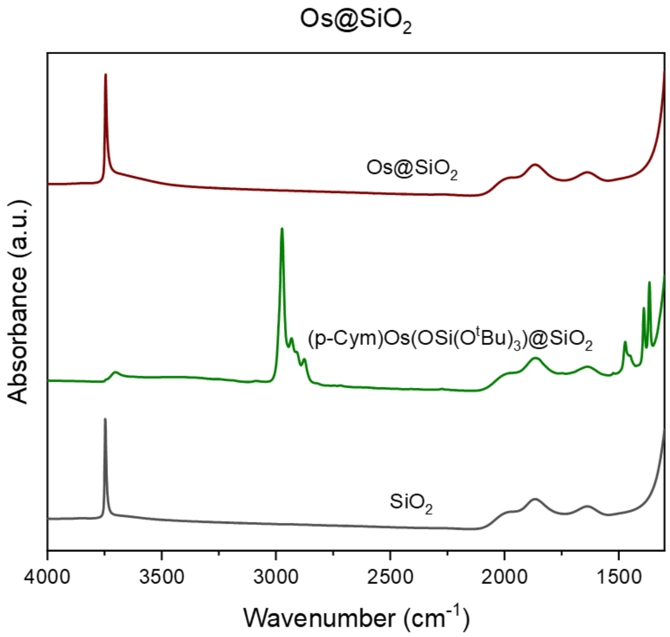

Figure S16. IR spectra throughout the synthesis of Os@SiO₂.

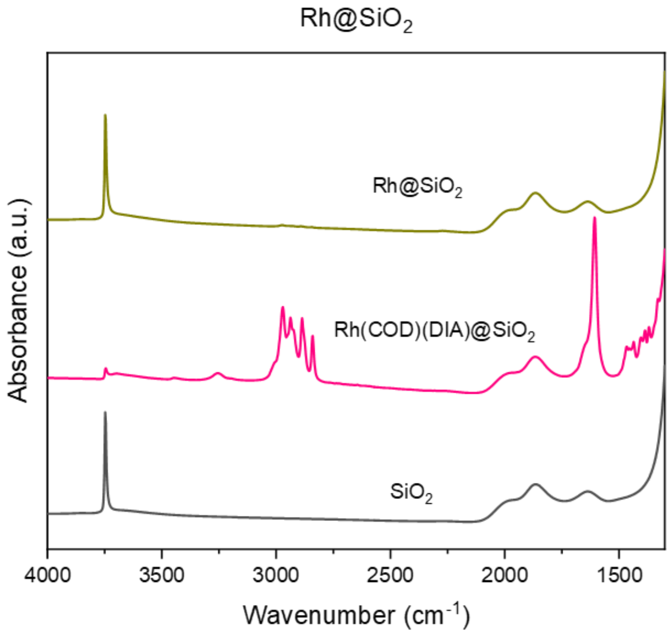

Figure S17. IR spectra throughout the synthesis of Rh@SiO$_2$.

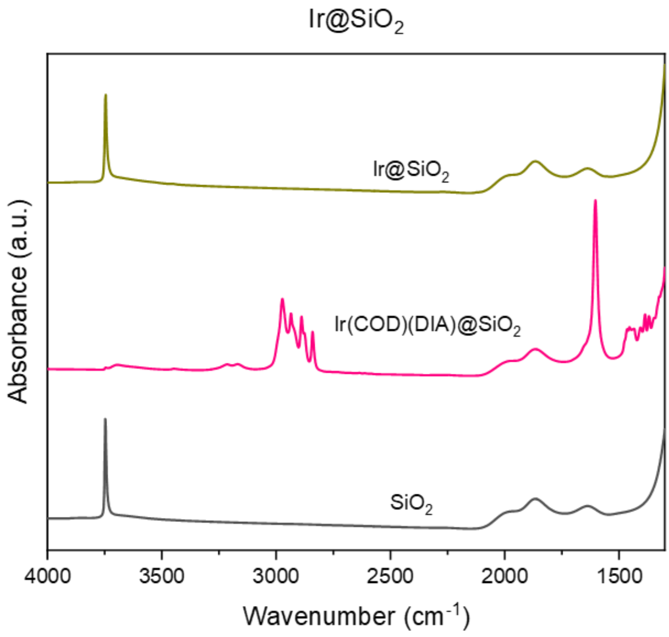

Figure S18. IR spectra throughout the synthesis of Ir@SiO$_2$.

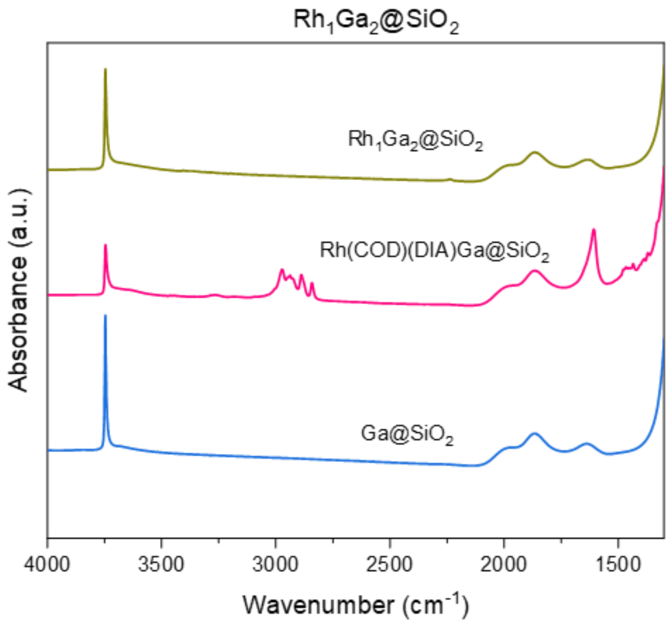

Figure S19. IR spectra throughout the synthesis of $Rh_1Ga_2@SiO_2$ starting from the second grafting.

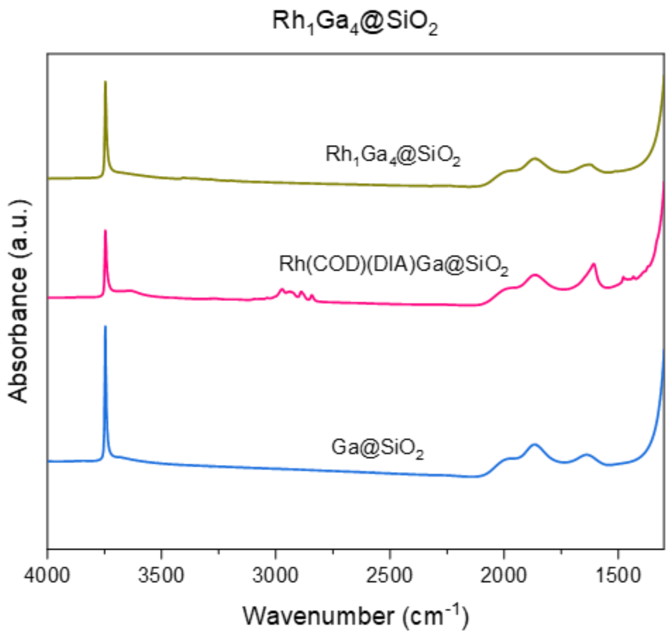

Figure S20. IR spectra throughout the synthesis of $Rh_1Ga_4@SiO_2$ starting from the second grafting.

Table S1. Summary of physicochemical properties of monometallic and Ga-promoted materials.

Catalyst	EA (wt%)	Metal density (M/nm^2)	*M*/Ga ratio[a]	Particle size (nm)[b]
Ru@SiO_2	Ru: 2.26	Ru: 0.67	-	2.0 ± 0.9
RuGa@SiO_2	Ru: 2.84<br>Ga: 1.74	Ru: 0.85<br>Ga: 0.75	1.13:1	1.7 ± 0.7
Os@SiO_2	Os: 3.23	Os: 0.51	-	1.6 ± 0.6
OsGa@SiO_2	Os: 4.56<br>Ga: 1.54	Os: 0.72<br>Ga: 0.67	1.07:1	1.8 ± 0.7
Rh@SiO_2	Rh: 2.26	Rh: 0.66	-	3.2 ± 1.0[c]
RhGa@SiO_2	Rh: 2.50<br>Ga: 1.78	Rh: 0.73<br>Ga: 0.77	0.95:1	1.7 ± 0.7
Ir@SiO_2	Ir: 4.89	Ir: 0.77	-	2.1 ± 0.8
IrGa@SiO_2	Ir: 4.63<br>Ga: 1.56	Ir: 0.73<br>Ga: 0.67	1.09:1	1.6 ± 0.6
Rh_1Ga_2@SiO_2	Rh: 1.16<br>Ga: 1.87	Rh: 0.34<br>Ga: 0.81	0.42:1	1.3 ± 0.6
Rh_1Ga_4@SiO_2	Rh: 0.52<br>Ga: 1.89	Rh: 0.15<br>Ga: 0.82	0.18:1	0.8 ± 0.3

[a] Determined by element analysis (EA); [b] particle size determined by STEM. [c] adapted from our previous work.[3]

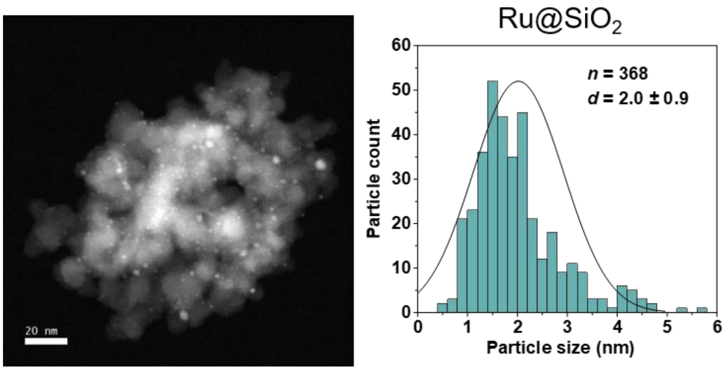

Figure S21. STEM analysis of Ru@SiO$_2$. [Left] High-angle annular dark-field STEM (HAADF-STEM) image of Ru nanoparticles supported on SiO$_2$, [Right] Particle size distribution of Ru@SiO$_2$.

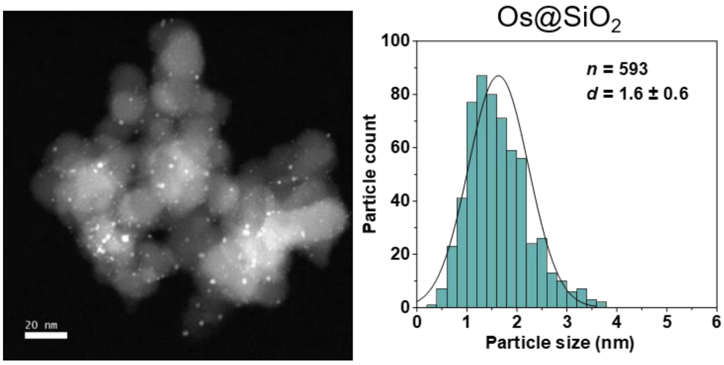

Figure S22. STEM analysis of Os@SiO$_2$. [Left] HAADF-STEM image of Os nanoparticles supported on SiO$_2$, [Right] Particle size distribution of Rh@SiO$_2$.

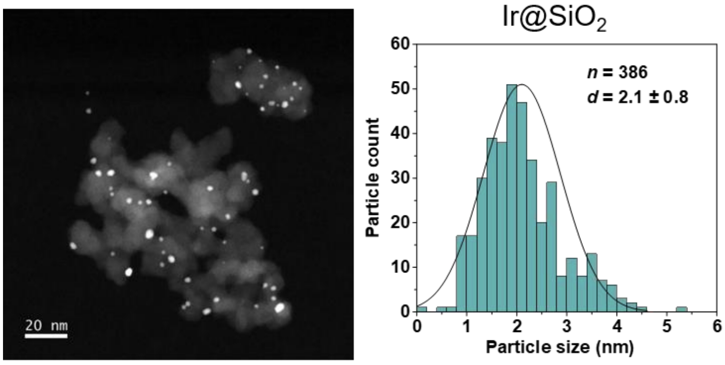

Figure S23. STEM analysis of Ir@SiO$_2$. [Left] HAADF-STEM image of Ir nanoparticles supported on SiO$_2$, [Right] Particle size distribution of Ir@SiO$_2$.

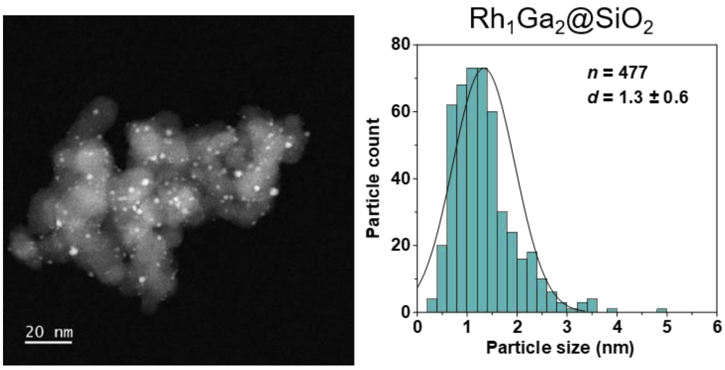

Figure S24. STEM analysis of Rh_1Ga_2@SiO_2. [Left] HAADF-STEM image of Rh_1Ga_2 nanoparticles supported on SiO_2, [Right] Particle size distribution of Rh_1Ga_2@SiO_2.

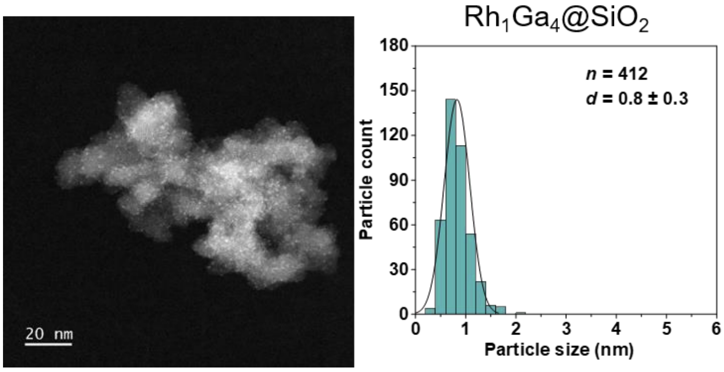

Figure S25. STEM analysis of Rh_1Ga_4@SiO_2. [Left] HAADF-STEM image of Rh_1Ga_4 nanoparticles supported on SiO_2, [Right] Particle size distribution of Rh_1Ga_4@SiO_2.

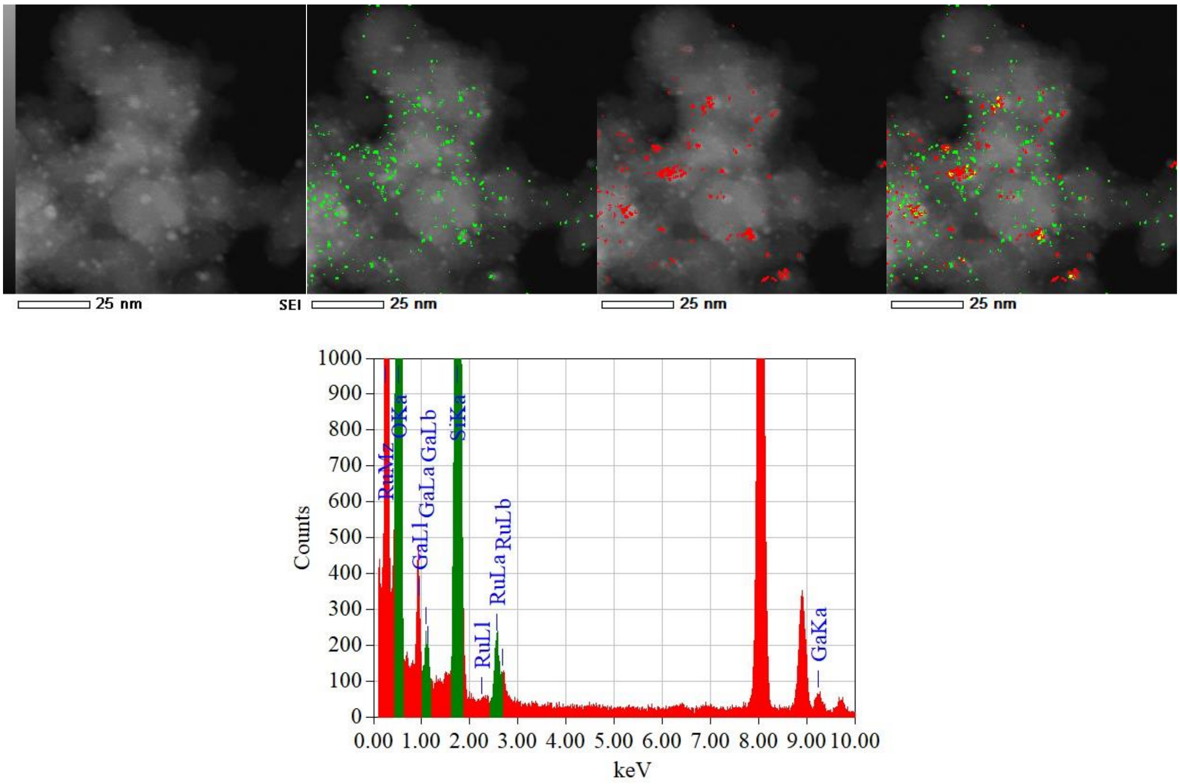

Figure S26. EDX analysis. [Top] EDX Maps of RuGa@SiO_2 (green = Ga, red = Ru), [Bottom] EDX-spectrum over the investigated area.

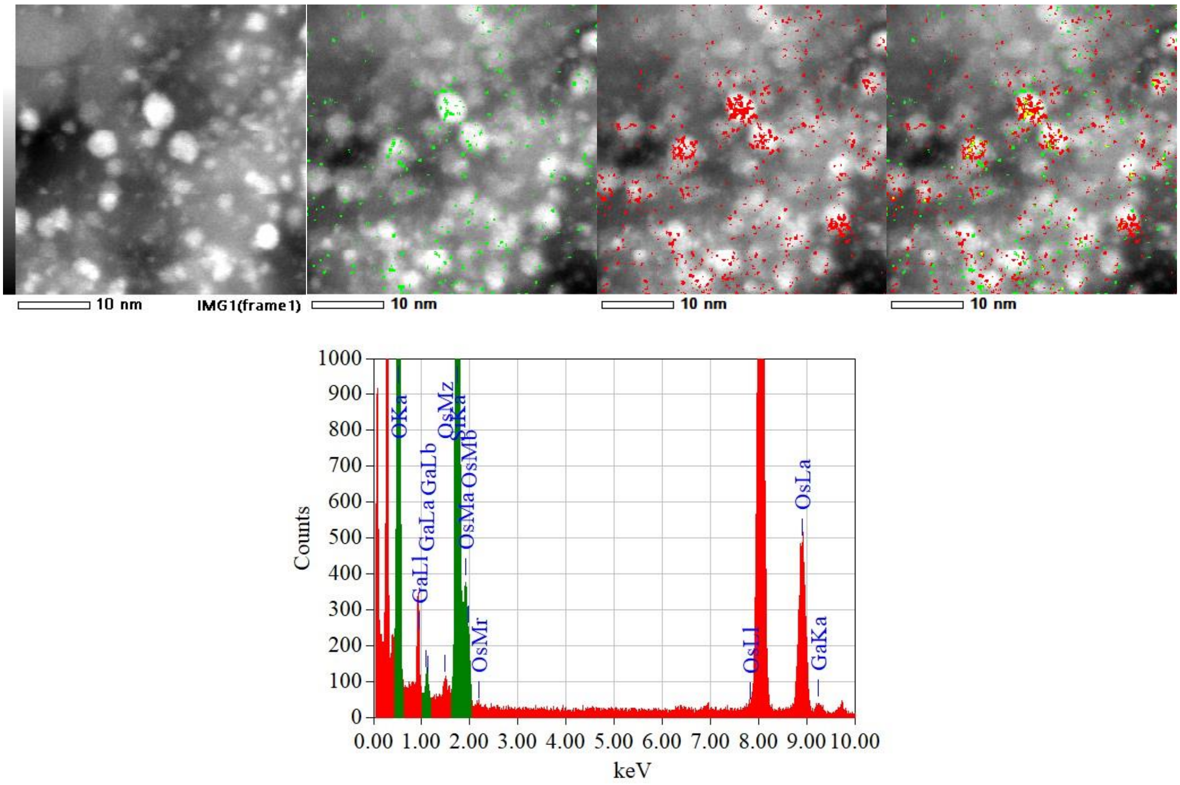

Figure S27. EDX analysis. [Top] EDX Maps of OsGa@SiO_2 (green = Os, red = Ga) [Bottom] EDX-spectrum over the investigated area.

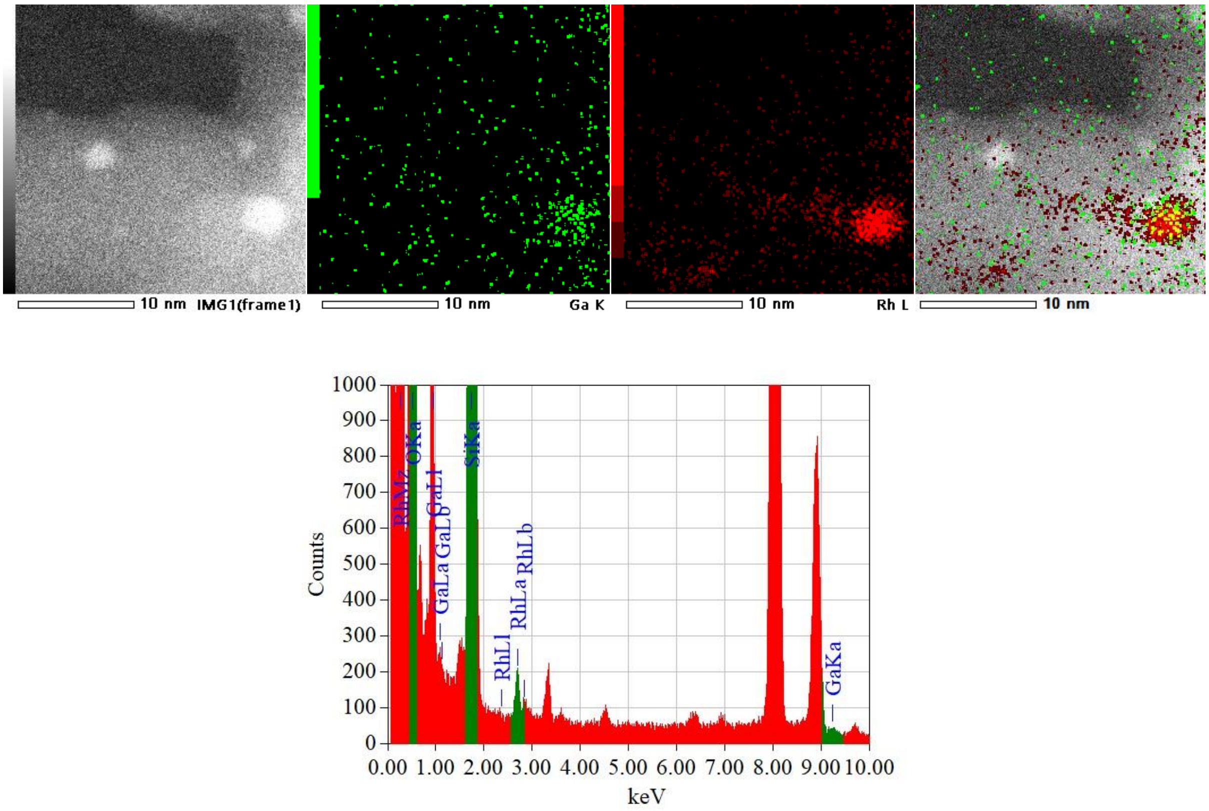

Figure S28. EDX analysis. [Top] EDX Maps of RhGa@SiO_2 (green = Ga, red = Rh), [Bottom] EDX-spectrum over the investigated area.

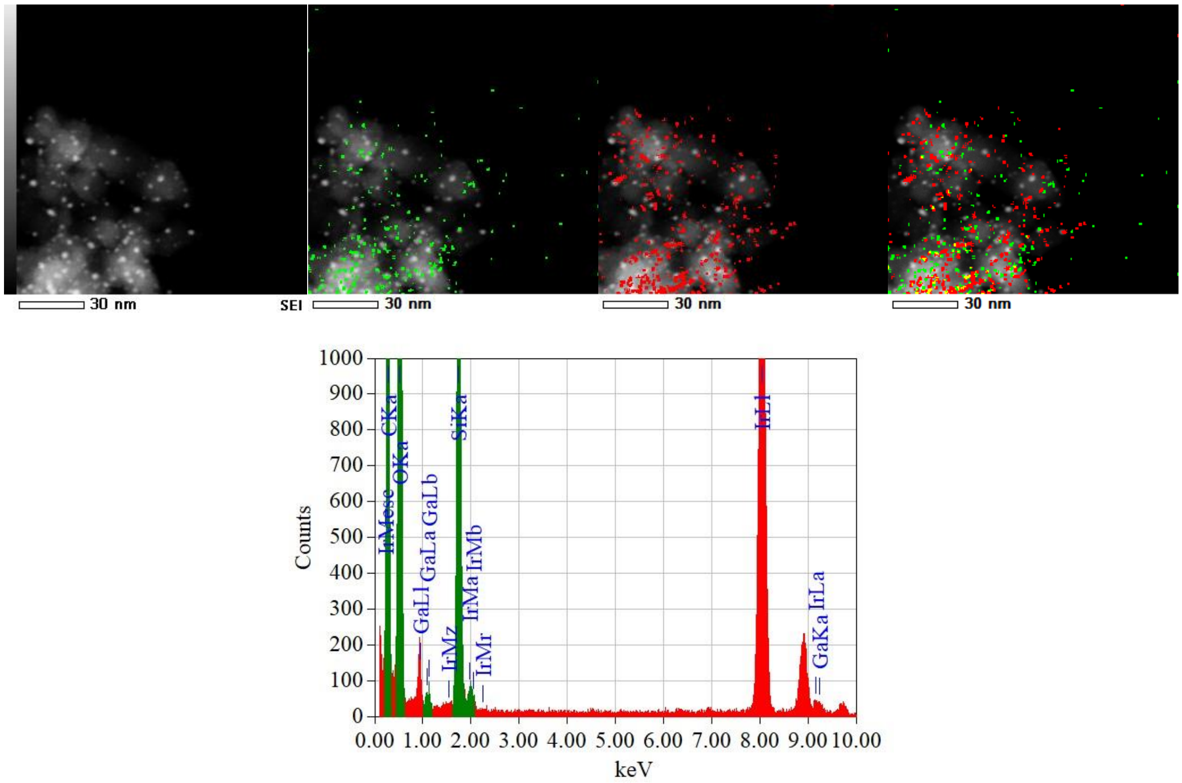

Figure S29. EDX analysis. [Top] EDX Maps of IrGa@SiO_2 (green = Ga, red = Ir), [Bottom] EDX-spectrum over the investigated area.

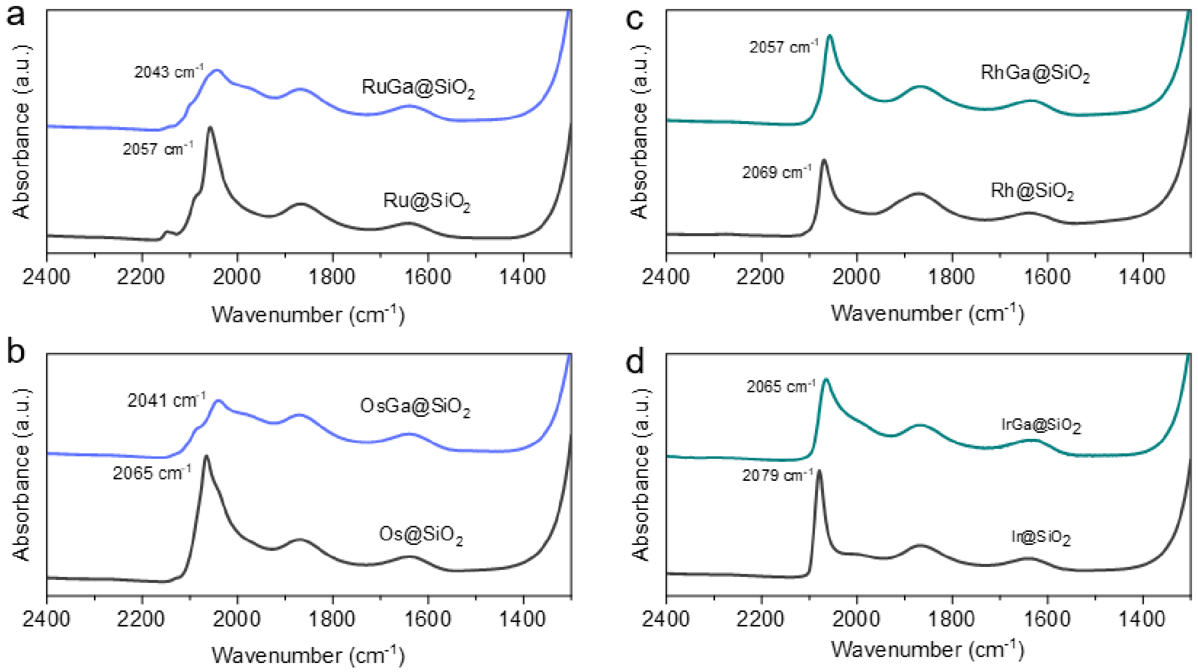

Figure S30. FTIR spectra of CO adsorbed on monometallic *M*@SiO$_2$ and Ga-promoted *M*Ga@SiO$_2$ under 12-13 mbar CO pressure at room temperature.

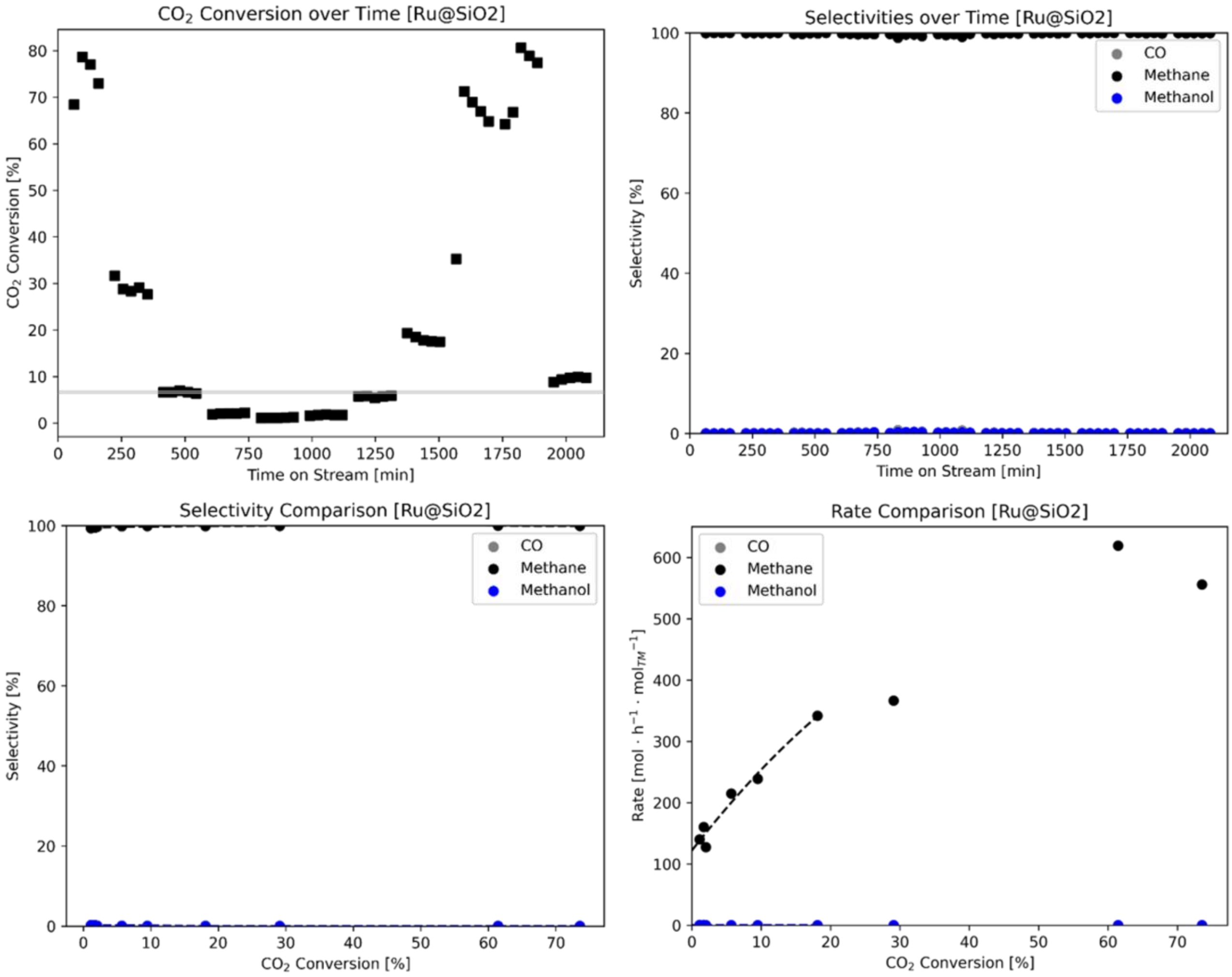

Figure S31. Catalytic data for Ru@SiO$_2$. Initial rate was extrapolated via a second order polynomial fit to the y axis. The selectivity at 1% CO_2 conversion is extracted via interpolation.

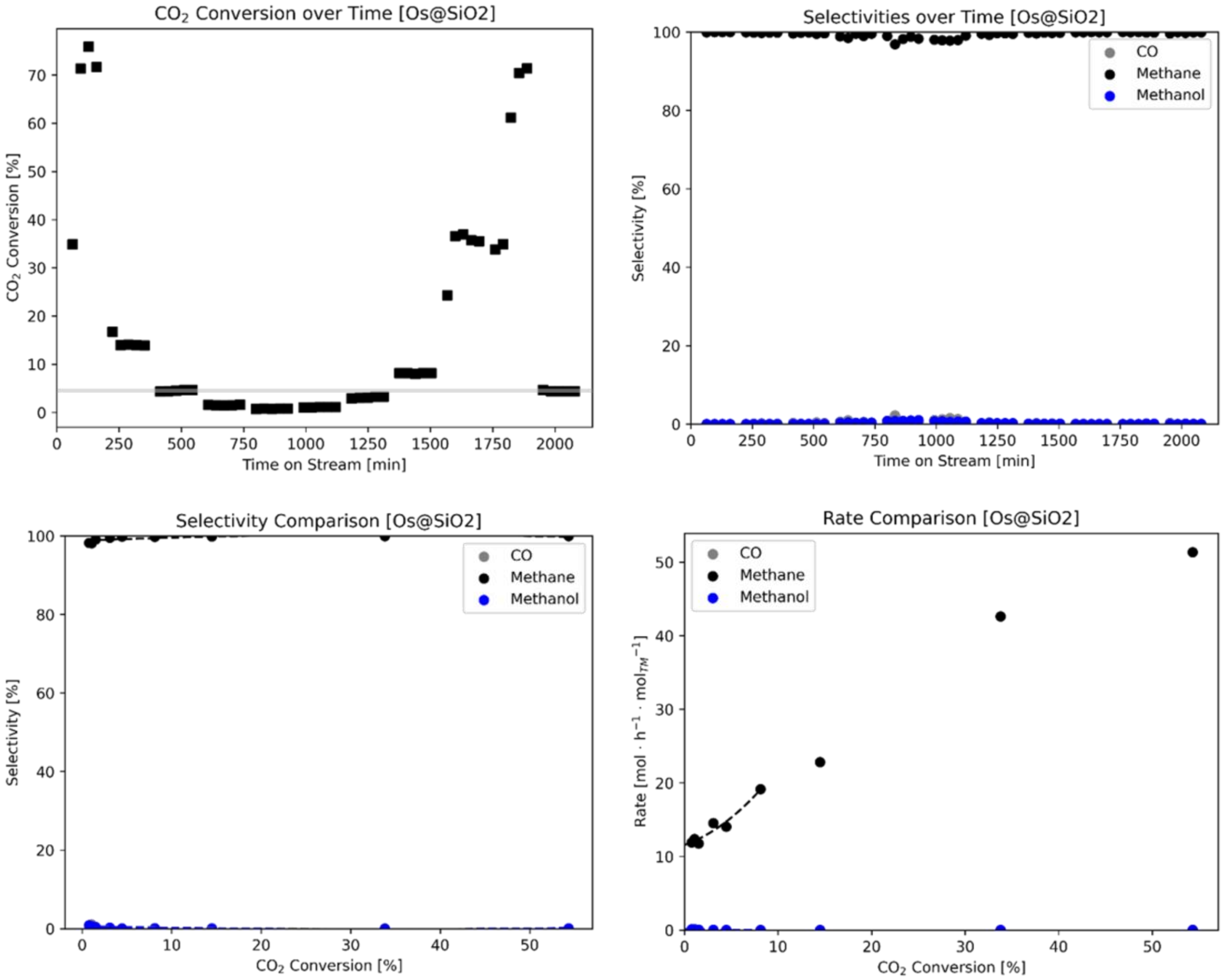

Figure S32. Catalytic data for Os@SiO$_2$. Initial rate was extrapolated via a second order polynomial fit to the y axis.

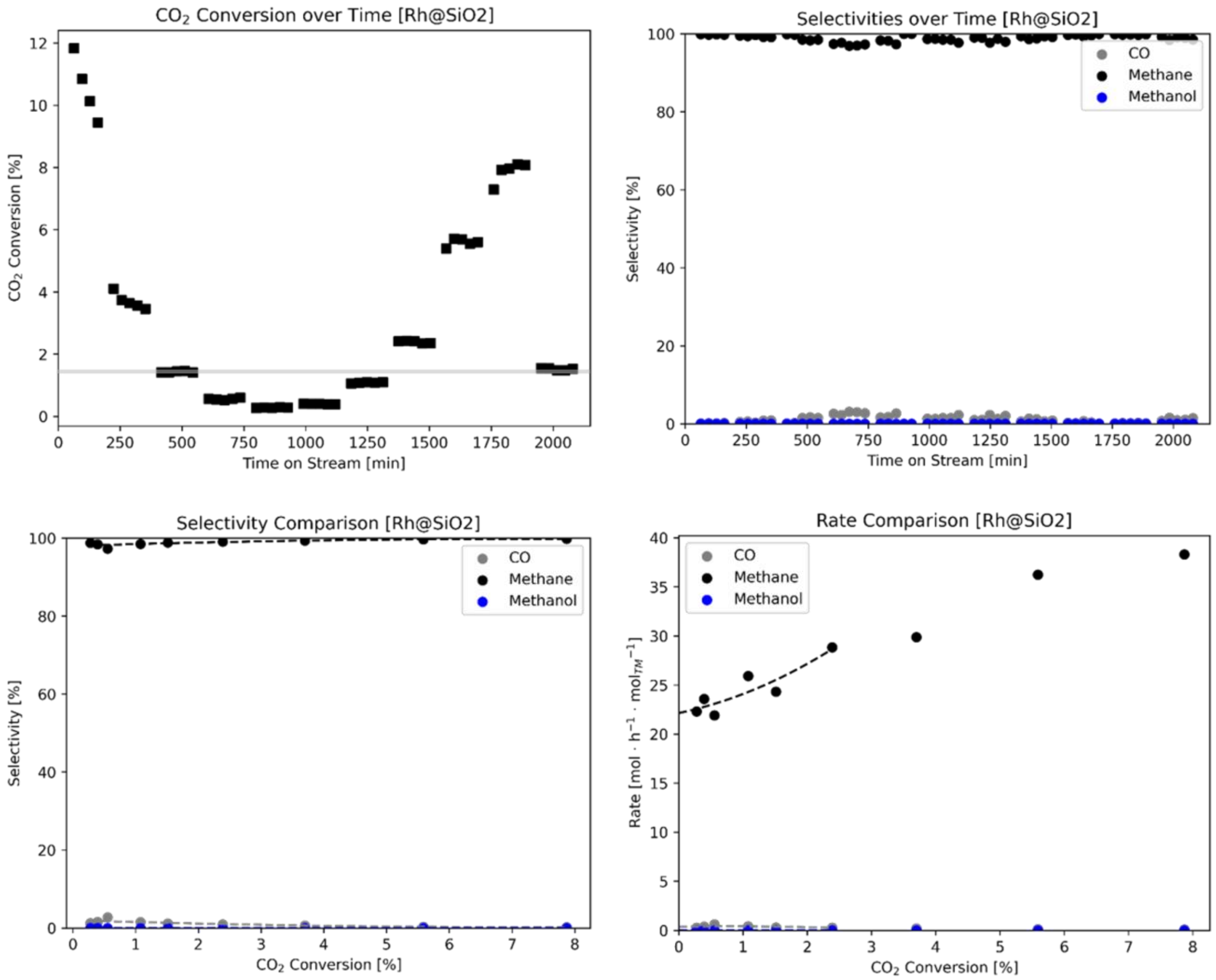

Figure S33. Catalytic data for Rh@SiO$_2$. Initial rate was extrapolated via a second order polynomial fit to the y axis.

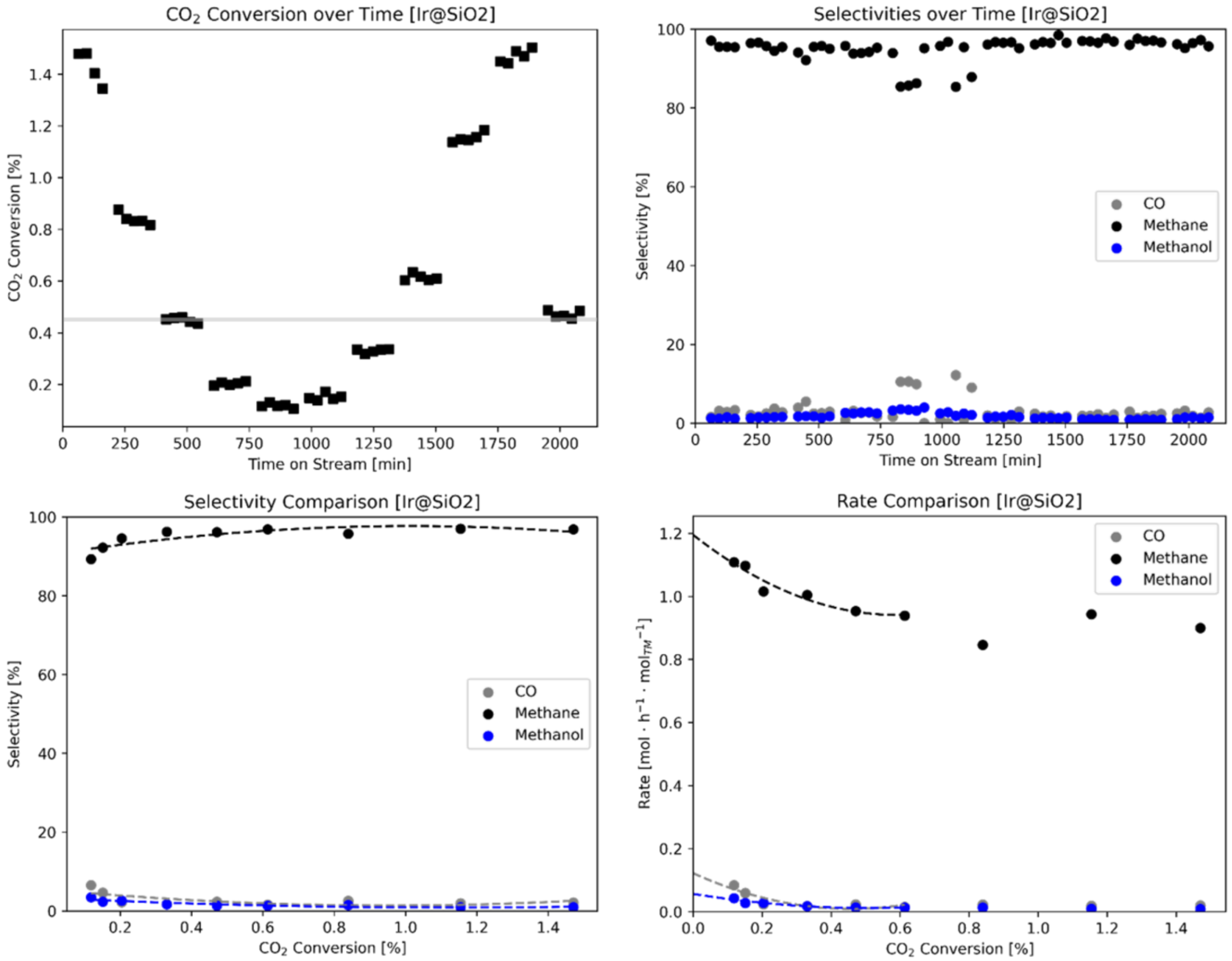

Figure S34. Catalytic data for Ir@SiO$_2$. Initial rate was extrapolated via a second order polynomial fit to the y axis.

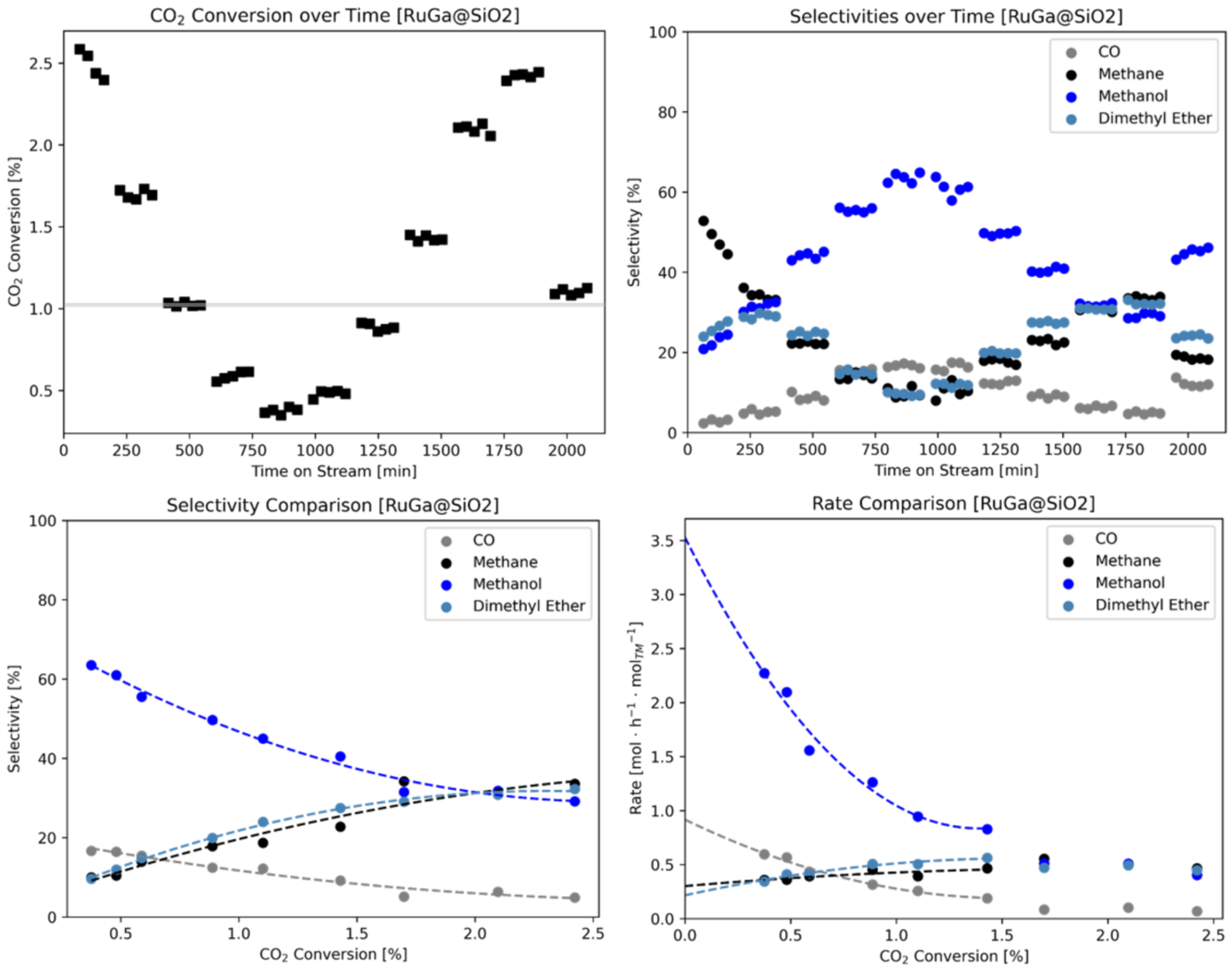

Figure S35. Catalytic data for RuGa@SiO$_2$. Initial rate was extrapolated via a second order polynomial fit to the y axis.

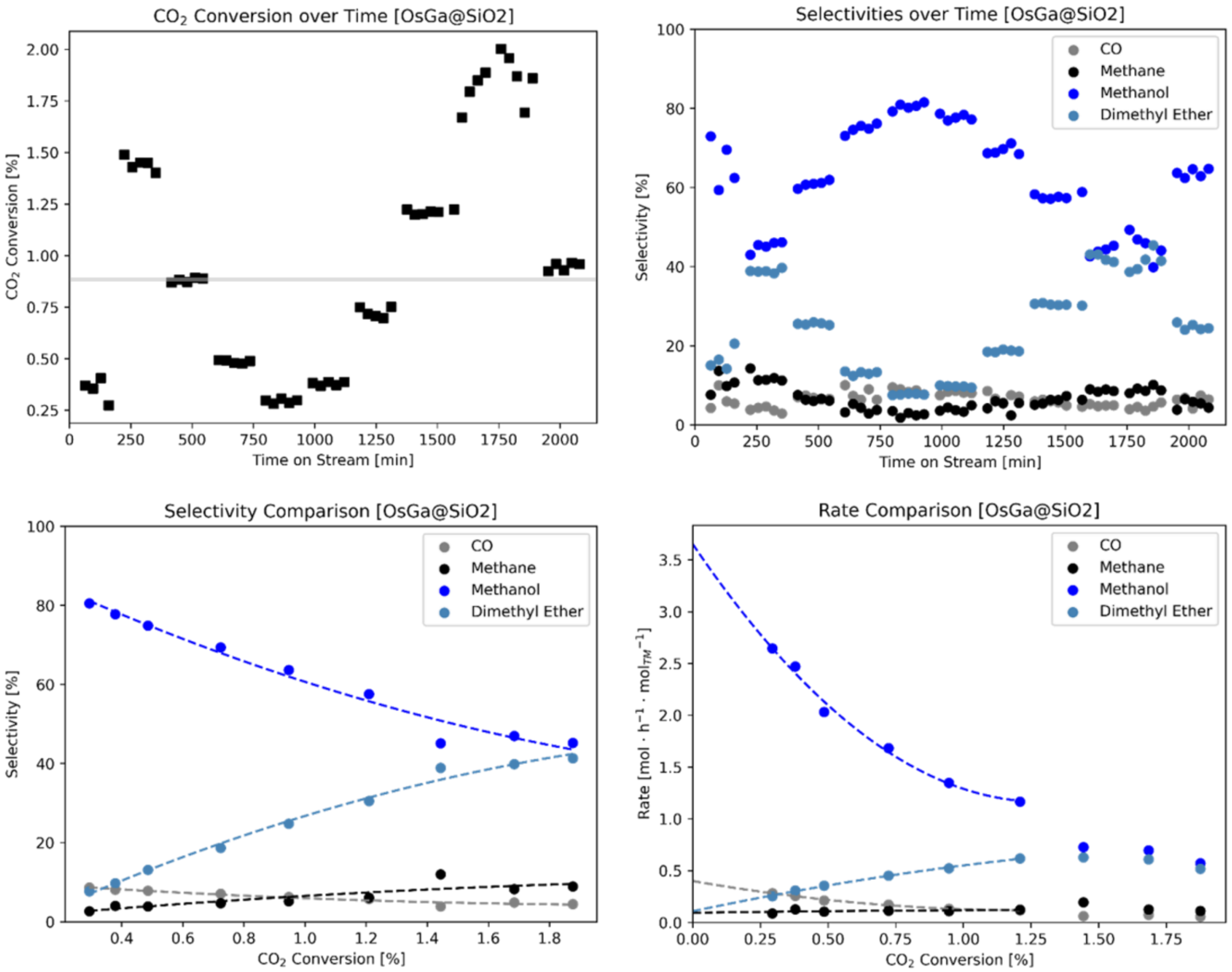

Figure S36. Catalytic data for OsGa@SiO$_2$. Initial rate was extrapolated via a second order polynomial fit to the y axis.

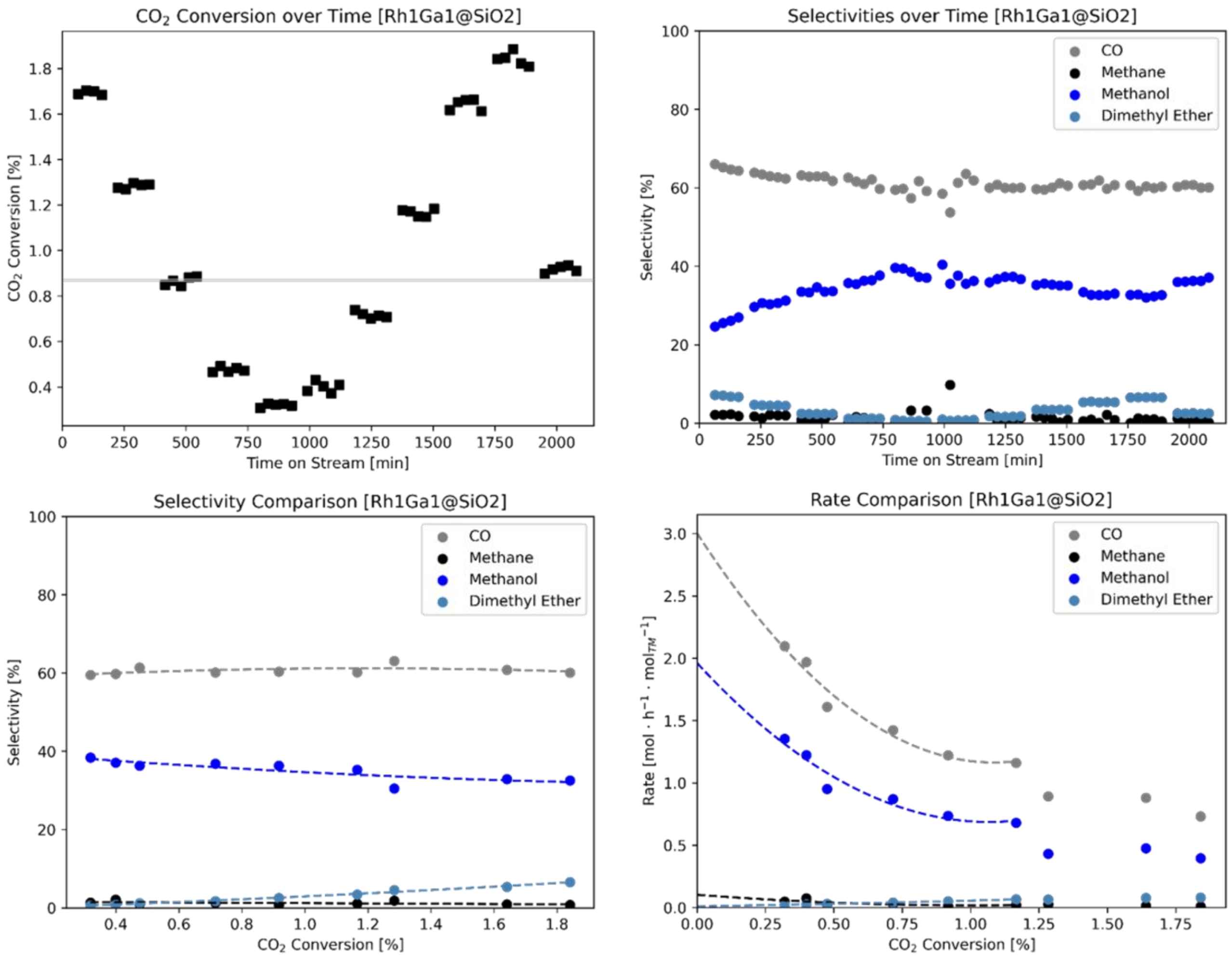

Figure S37. Catalytic data for RhGa@SiO$_2$. Initial rate was extrapolated via a second order polynomial fit to the y axis.

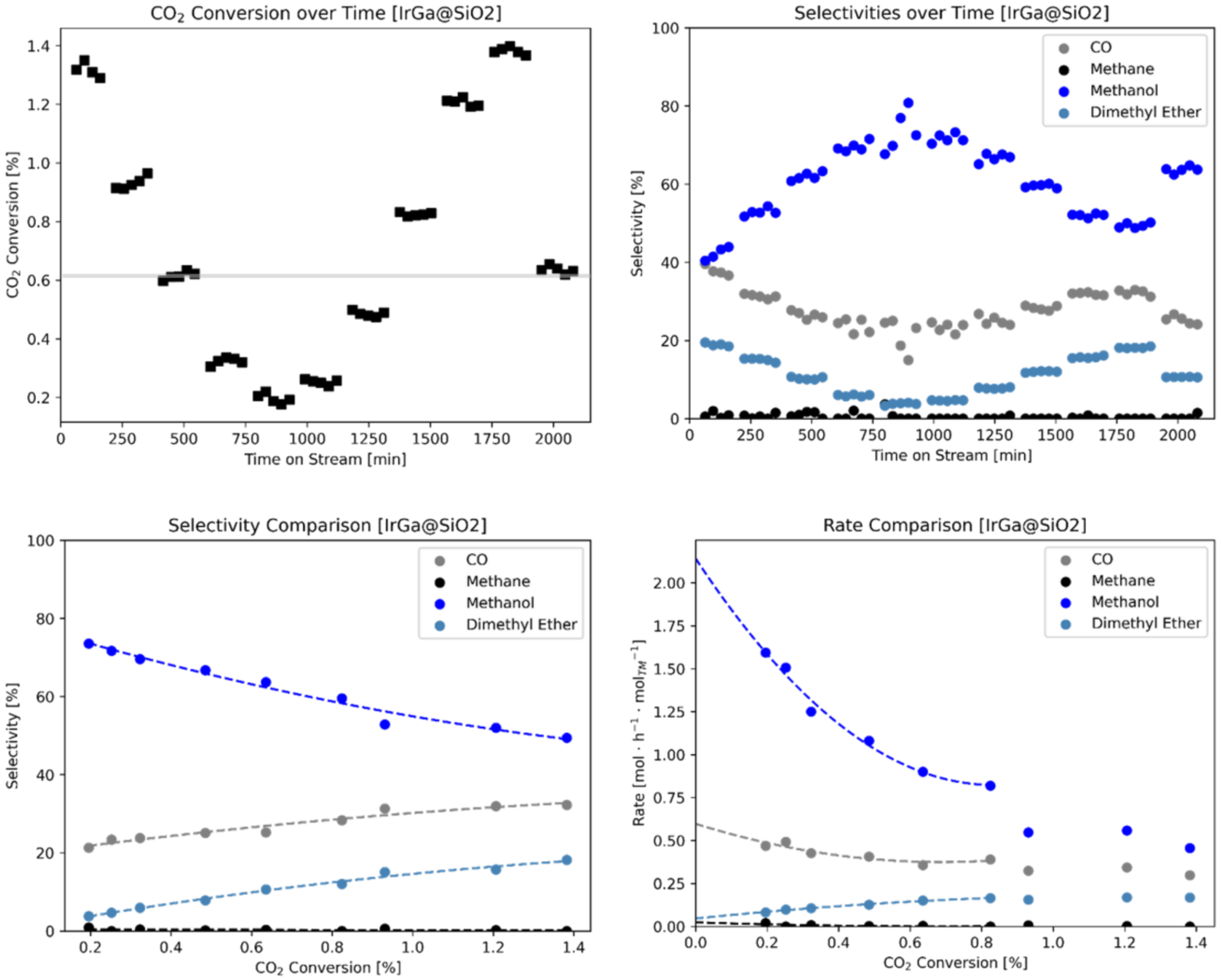

Figure S38. Catalytic data for IrGa@SiO$_2$. Initial rate was extrapolated via a second order polynomial fit to the y axis.

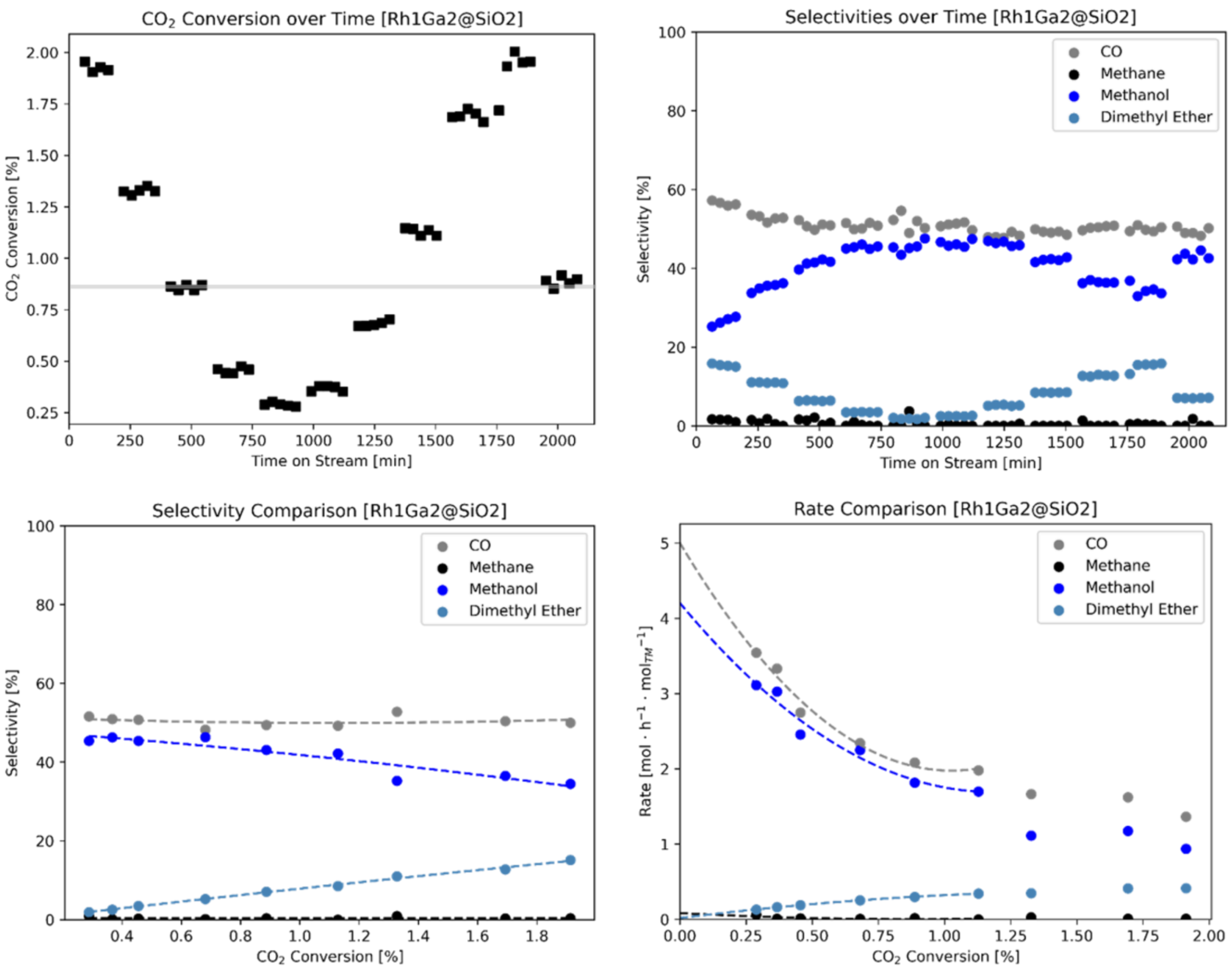

Figure S39. Catalytic data for $Rh_1Ga_2@SiO_2$. Initial rate was extrapolated via a second order polynomial fit to the y axis.

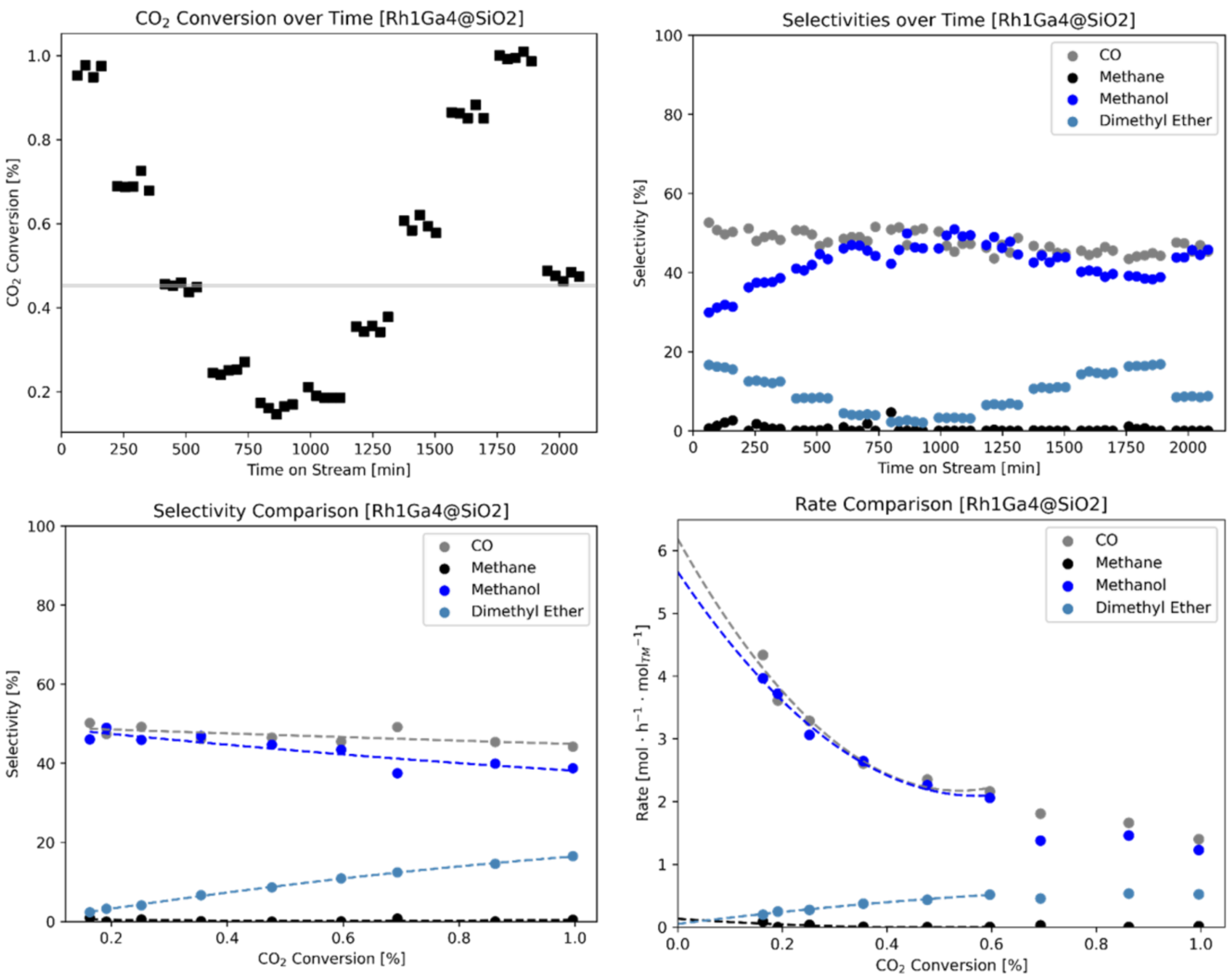

Figure S40. Catalytic data for $Rh_1Ga_4@SiO_2$. Initial rate was extrapolated via a second order polynomial fit to the y axis.

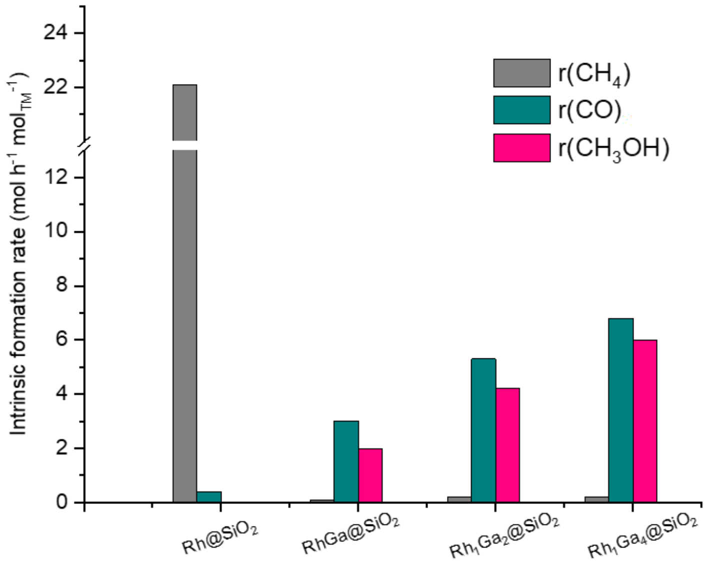

Figure S41. Intrinsic formation rate over Rh@SiO₂ and RhGa@SiO₂ with different Rh/Ga ratio catalysts. Reaction conditions: *F* = 6-100 mL/min, *T* = 230 ºC, *P* = 40 bar.

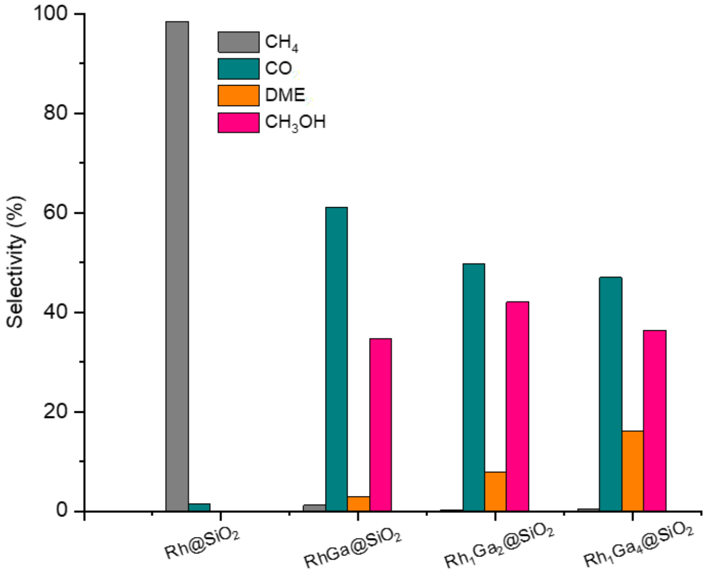

Figure S42. Product selectivity over Rh@SiO₂ and RhGa@SiO₂ with different Rh/Ga ratio catalysts at 1% CO₂ conversion. Reaction conditions: *F* = 6-100 mL/min, *T* = 230 ºC, *P* = 40 bar.

Table S2. Summary of intrinsic formation rate and selectivities over different catalysts for CO_2 hydrogenation. [a]

Catalyst	Intrinsic formation rate (mol h^{-1} mol_{TM}^{-1})			Total intrinsic formation rate (mol mol_{TM}^{-1} h^{-1})	Intrinsic CH_4 Select. (%)	Intrinsic CO Select. (%)	Intrinsic CH_3OH Select. (%)
	CH_4	CO	CH_3OH				
Ru@SiO_2	121.8	0.4	0.4	122.6	99.3	0.3	0.3
RuGa@SiO_2	0.3	0.9	3.5	4.7	6.4	19.1	74.5
Os@SiO_2	11.6	0.1	0.1	11.8	98.3	0.8	0.8
OsGa@SiO_2	0.1	0.4	3.7	4.2	2.4	9.5	88.1
Rh@SiO_2	22.1	0.4	0	22.5	98.2	1.8	0
RhGa@SiO_2	0.1	3.0	2.0	5.1	2.0	58.8	39.2
Rh_1Ga_2@SiO_2	0.2	5.3	4.2	9.7	2.1	54.6	43.3
Rh_1Ga_4@SiO_2	0.2	6.8	6.0	13.0	1.5	52.3	46.2
Ir@SiO_2	1.2	0.1	0.1	1.4	85.7	7.1	7.1
IrGa@SiO_2	0	0.6	2.1	2.7	0	22.2	77.8

[a] Reaction conditions: 20 mg for Ru@SiO_2, 30 mg for Rh@SiO_2, 200 mg for the other catalysts, F = 6-100 mL/min, T = 230 ^{o}C, P = 40 bar.

Table S3. Comparison of conditions, activity and selectivity to methanol over different catalysts for CO_2 hydrogenation.

Catalyst	Gas composition	Pressure (bar)	Temperature (°C)	CH_3OH selectivity (%)	Intrinsic CH_3OH rate (mol mol_{TM}^{-1} h^{-1})	Refs
$CuZr@SiO_2$	1:3:1 CO_2:H_2:Ar	25	230	77	2.2	[16]
$CuGa@SiO_2$	1:3:1 CO_2:H_2:Ar	25	230	90	2.8	[17]
$CuZn@SiO_2$	1:3:1 CO_2:H_2:Ar	25	230	86	3.2	[18]
$PdGa@SiO_2$	1:3:1 CO_2:H_2:Ar	25	230	80	23.0	[2]
$AuZn@SiO_2$	1:3:1 CO_2:H_2:Ar	25	230	65	4.2	[19]
$NiGa@SiO_2$[a]	1:3:1 CO_2:H_2:Ar	25	230	60	4.0	[20]
$PtGa@SiO_2$	1:3:1 CO_2:H_2:Ar	25	230	54	7.2	[21]
$RuGa@SiO_2$	1:3:1 CO_2:H_2:Ar	40	230	75	3.5	This work
$OsGa@SiO_2$	1:3:1 CO_2:H_2:Ar	40	230	88	3.7	This work
$RhGa@SiO_2$	1:3:1 CO_2:H_2:Ar	40	230	39	2.0	This work
$Rh_1Ga_2@SiO_2$	1:3:1 CO_2:H_2:Ar	40	230	43	4.2	This work
$Rh_1Ga_4@SiO_2$	1:3:1 CO_2:H_2:Ar	40	230	46	6.0	This work
$IrGa@SiO_2$	1:3:1 CO_2:H_2:Ar	40	230	78	2.1	This work

[a] The formation rate and selectivity was obtained at 60000 ml $h^{-1}g_{cat}^{-1}$.

Table S4. Summary of selectivities over different catalysts at 1% CO_2 conversion. [a]

Catalyst	CH_4 select. (%)	CO select. (%)	DME select. (%)	CH_3OH select. (%)	CH_3OH/DME select. (%)
Ru@SiO_2	99.6	0.2	0	0.2	0.2
RuGa@SiO_2	19.6	11.7	21.8	46.7	68.5
Os@SiO_2	98.9	0.5	0	0.5	0.5
OsGa@SiO_2	6.5	5.9	26.7	60.7	87.4
Rh@SiO_2	98.5	1.5	0	0	0
RhGa@SiO_2	1.2	61.1	2.9	34.7	37.6
Rh_1Ga_2@SiO_2	0.3	49.7	7.9	42.1	50.0
Rh_1Ga_4@SiO_2	0.5	47.0	16.2	36.4	52.6
Ir@SiO_2	97.7	1.4	0	0.9	0.9
IrGa@SiO_2	0.2	30.2	14.6	54.9	69.5

[a] Reaction conditions: 20 mg for Ru@ SiO_2, 30 mg for Rh@SiO_2, 200 mg for the other catalysts, F = 6-100 mL/min, T = 230 °C, P = 40 bar.

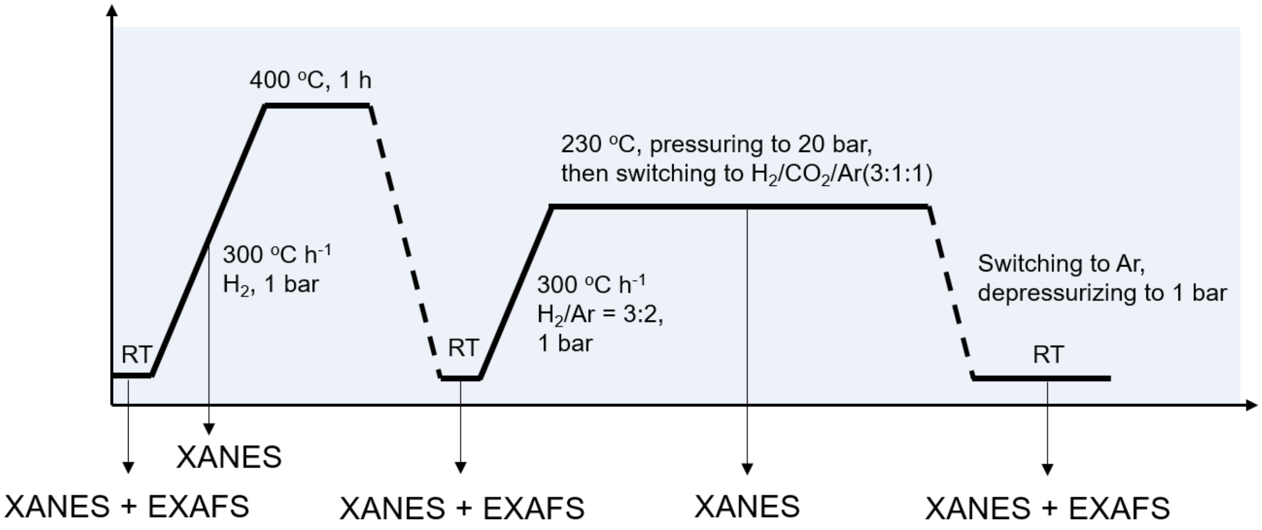

Figure S43. Profile for the details including temperature, pressure, and gas composition throughout the in situ XAS experiments.

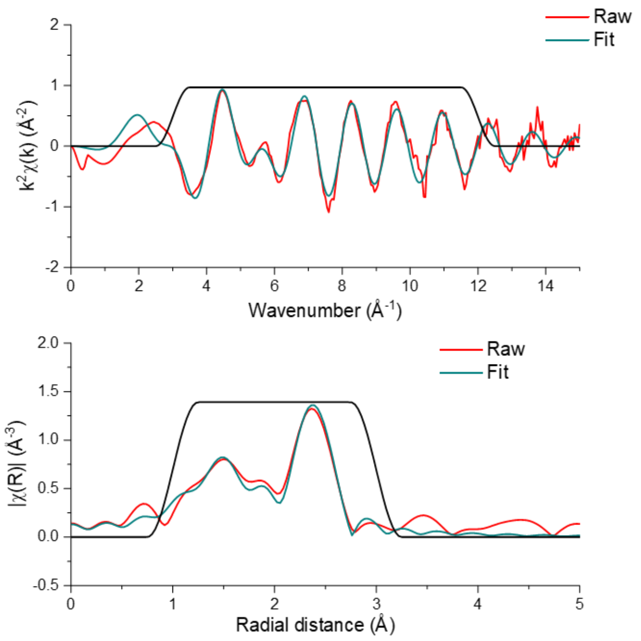

Figure S44. EXAFS fit for air exposed Ru@SiO_2. (top) K-space with raw (red) and fitted (grey) data. Window (blue) 3.0-12 Å^{-1}, k-weight = 2, Hanning window, dk = 1; (bottom) R-space with raw (red) and fitted (grey) data. Window (blue) 1-3. Å, k-weight = 2, Hanning window, dk = 0.5. Fit summarized in Table S5.

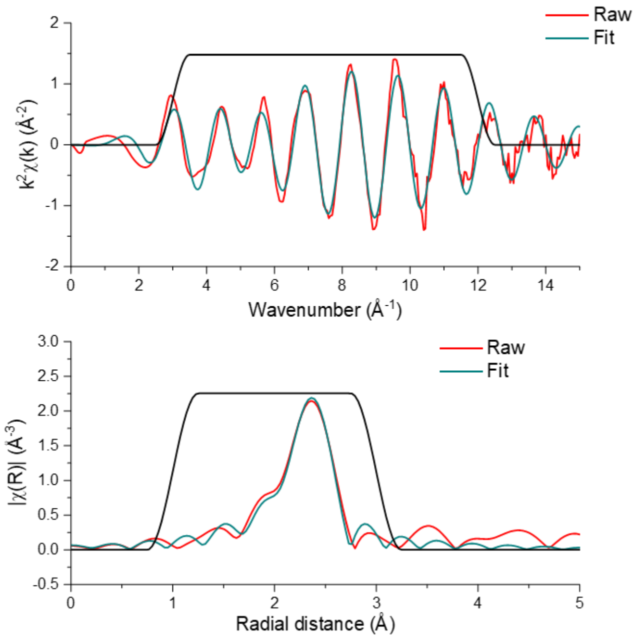

Figure S45. EXAFS fit for H_2 reduced Ru@SiO_2. (top) K-space with raw (red) and fitted (grey) data. Window (blue) 3.0-12 Å^{-1}, k-weight = 2, Hanning window, dk = 1; (bottom) R-space with raw (red) and fitted (grey) data. Window (blue) 1-3. Å, k-weight = 2, Hanning window, dk = 0.5. Fit summarized in Table S5.

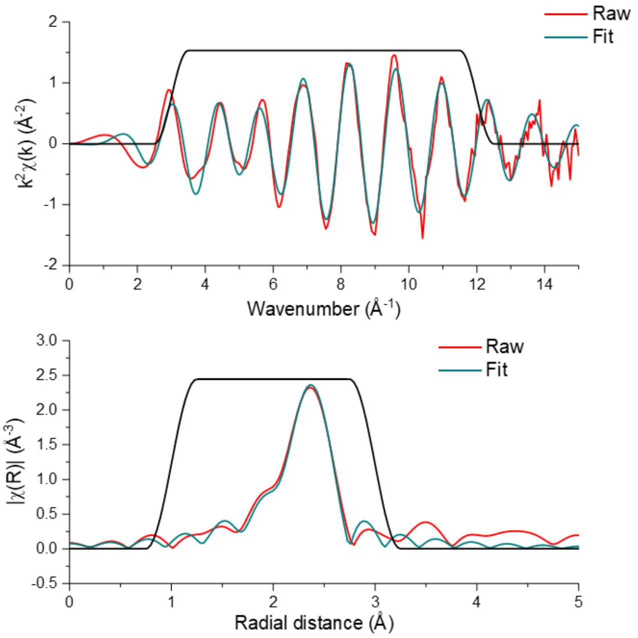

Figure S46. EXAFS fit for post CO_2 hydrogenation Ru@SiO_2. (top) K-space with raw (red) and fitted (grey) data. Window (blue) 3.0-12 Å^{-1}, k-weight = 2, Hanning window, dk = 1; (bottom) R-space with raw (red) and fitted (grey) data. Window (blue) 1-3. Å, k-weight = 2, Hanning window, dk = 0.5. Fit summarized in Table S5.

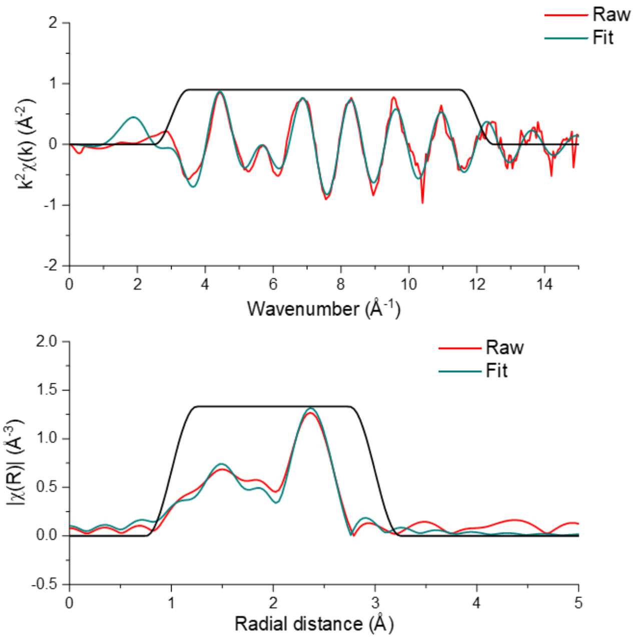

Figure S47. EXAFS fit for air exposed RuGa@SiO_2. (top) K-space with raw (red) and fitted (grey) data. Window (blue) 3.0-12 Å^{-1}, k-weight = 2, Hanning window, dk = 1; (bottom) R-space with raw (red) and fitted (grey) data. Window (blue) 1-3. Å, k-weight = 2, Hanning window, dk = 0.5. Fit summarized in Table S5.

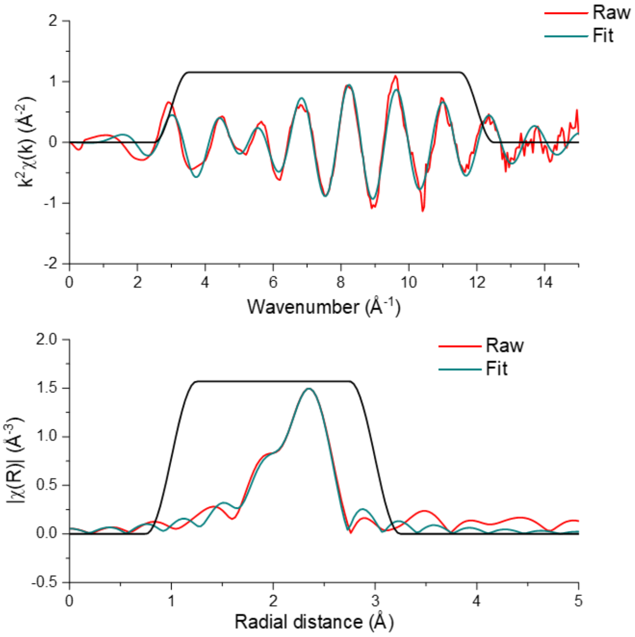

Figure S48. EXAFS fit for H_2 reduced RuGa@SiO_2. (top) K-space with raw (red) and fitted (grey) data. Window (blue) 3.0-12 Å^{-1}, k-weight = 2, Hanning window, dk = 1; (bottom) R-space with raw (red) and fitted (grey) data. Window (blue) 1-3. Å, k-weight = 2, Hanning window, dk = 0.5. Fit summarized in Table S5.

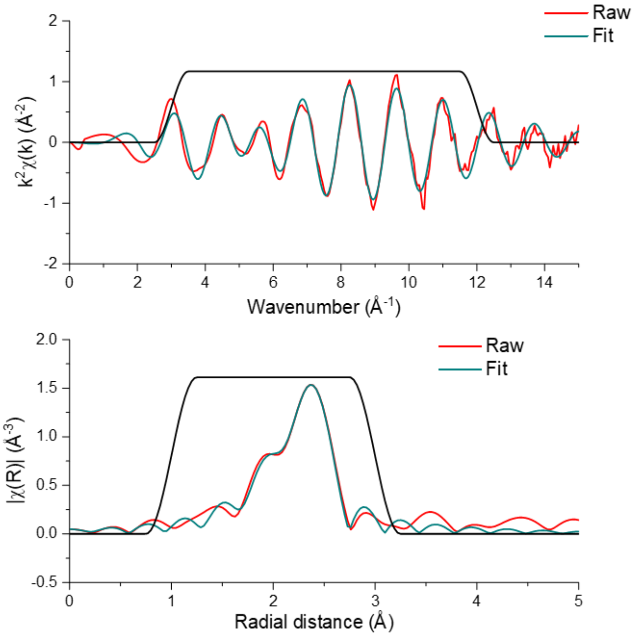

Figure S49. EXAFS fit for post CO_2 hydrogenation RuGa@SiO_2. (top) K-space with raw (red) and fitted (grey) data. Window (blue) 3.0-12 Å^{-1}, k-weight = 2, Hanning window, dk = 1; (bottom) R-space with raw (red) and fitted (grey) data. Window (blue) 1-3. Å, k-weight = 2, Hanning window, dk = 0.5. Fit summarized in Table S5.

Table S5. Summary of Ru K-edge fitting results of $RuSiO_2$ and RuGa@SiO_2 catalysts under different conditions.[a]

Catalyst	Conditions	Path	CN[b]	σ^2 (Å^2)[c]	ΔE (eV)[d]	R (Å)[e]
Ru@SiO_2	Air	Ru-O	4.2(1.4)	0.0107(0.0030)	-2.8(1.4)	1.99(0.01)
		Ru-Ru	5.1(0.9)	0.0060(0.0011)	-2.8(1.4)	2.68(0.02)
	H_2 reduction	Ru-Ru	8.1(1.0)	0.0056(0.0007)	-5.2(0.8)	2.66(0.01)
	Post CO_2 hydro.	Ru-Ru	8.7(1.0)	0.0056(0.0007)	-5.2(0.9)	2.67(0.01)
RuGa@SiO_2	Air	Ru-O	3.7(1.3)	0.0099(0.0049)	-3.4(1.5)	2.01(0.01)
		Ru-Ru	5.0(1.0)	0.0061(0.0012)	-3.4(1.5)	2.67(0.02)
	H_2 reduction	Ru-Ga	1.6(0.7)	0.0066(0.0010)	-6.2(1.6)	2.52(0.02)
		Ru-Ru	6.2(1.1)	0.0066(0.0010)	-6.2(1.6)	2.67(0.01)
	Post CO_2 hydro.	Ru-Ga	1.5(0.6)	0.0063(0.009)	-4.6(1.3)	2.53(0.02)
		Ru-Ru	6.3(1.1)	0.0063(0.009)	-4.6 (1.3)	2.66(0.01)

[a] $3.0 < k < 12$; S_o^2 was fixed as 0.82 (from Ru foil); $1 < R < 3$; k-weight = 2. [b] coordination number. [c] Debye-Waller parameter. [d] energy correction factor. [e] interatomic distance.

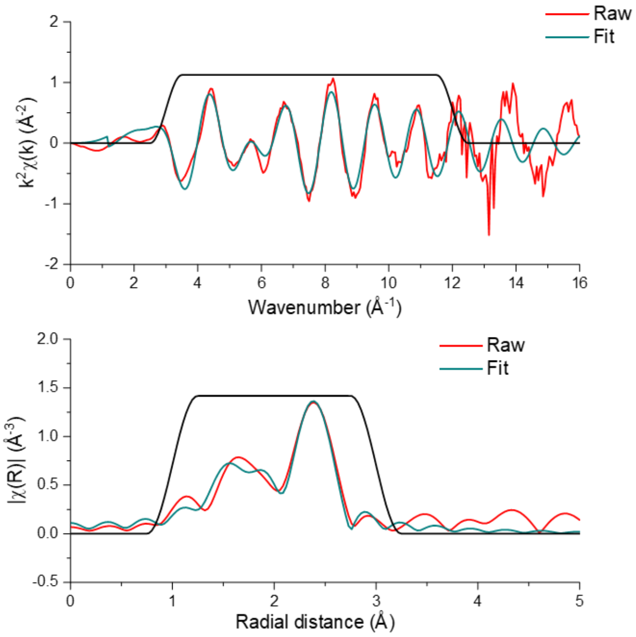

Figure S50. EXAFS fit for air exposed RhGa@SiO_2. (top) K-space with raw (red) and fitted (grey) data. Window (blue) 3.0-12 $Å^{-1}$, k-weight = 2, Hanning window, dk = 1; (bottom) R-space with raw (red) and fitted (grey) data. Window (blue) 1-3. Å, k-weight = 2, Hanning window, dk = 0.5. Fit summarized in Table S6.

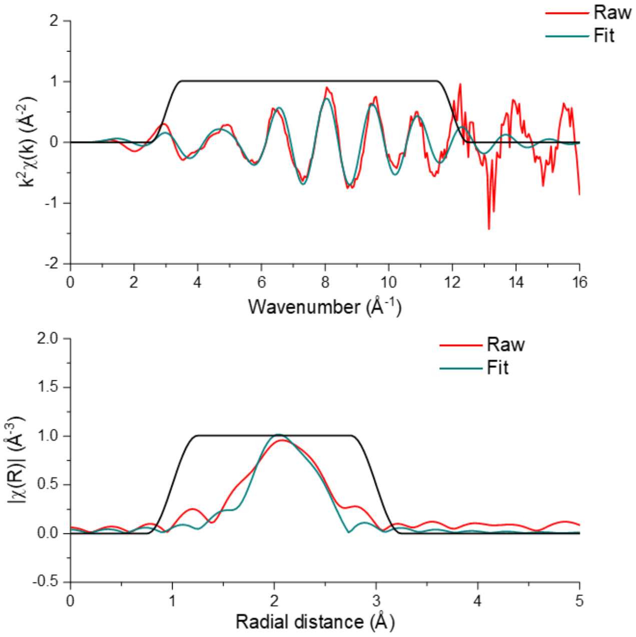

Figure S51. EXAFS fit for H_2 reduced RhGa@SiO_2. (top) K-space with raw (red) and fitted (grey) data. Window (blue) 3.0-12 $Å^{-1}$, k-weight = 2, Hanning window, dk = 1; (bottom) R-space with raw (red) and fitted (grey) data. Window (blue) 1-3. Å, k-weight = 2, Hanning window, dk = 0.5. Fit summarized in Table S6.

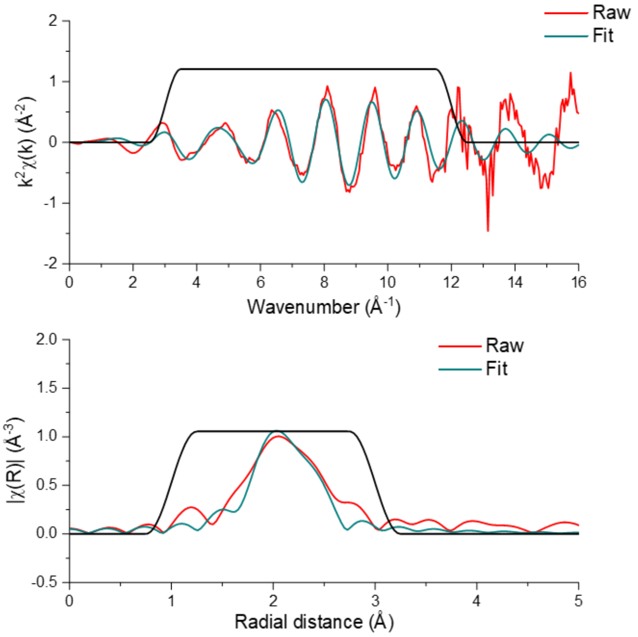

Figure S52. EXAFS fit for post CO_2 hydrogenation RhGa@SiO_2. (top) K-space with raw (red) and fitted (grey) data. Window (blue) 3.0-12 $Å^{-1}$, k-weight = 2, Hanning window, dk = 1; (bottom) R-space with raw (red) and fitted (grey) data. Window (blue) 1-3. Å, k-weight = 2, Hanning window, dk = 0.5. Fit summarized in Table S6.

Table S6. Summary of Rh K-edge fitting results of $RhSiO_2$ and $RhGa@SiO_2$ catalysts under different conditions.[a]

**Catalyst**	**Conditions**	**Path**	**CN[b]**	**σ^2 (Å^2)[c]**	**ΔE (eV)[d]**	**R (Å)[e]**
$Rh@SiO_2$	Air	Rh-Rh	8.7(0.7)	0.0046(0.0005)	-6.1(0.7)	2.68(0.01)
	H_2 reduction	Rh-Rh	10.1(0.8)	0.0030(0.0004)	-5.9(0.7)	2.68(0.01)
	Post CO_2 hydro.	Rh-Rh	8.6(0.7)	0.0051(0.0005)	-6.4(0.7)	2.68(0.01)
$RhGa@SiO_2$	Air	Rh-O	2.2(0.5)	0.0043(0.0020)	-6.2(2.7)	2.09(0.04)
		Rh-Rh	4.0(1.3)	0.0043(0.0020)	5.2(6.5)	2.67(0.01)
	H_2 reduction	Rh-Ga	3.6(1.9)	0.0072(0.0032)	-9.1(6.8)	2.51(0.04)
		Rh-Rh	2.3(2.0)	0.0072 (0.0032)	-9.1(6.8)	2.65(0.04)
	Post CO_2 hydro.	Rh-Ga	2.8(1.2)	0.0058(0.027)	-9.4(5.7)	2.51(0.03)
		Rh-Rh	2.0(1.5)	0.0058(0.027)	-9.4(5.7)	2.64(0.03)

[a] $3.0 < k < 12$, S_o^2 was fixed as 0.85 (from Rh foil); $1 < R < 3$; k-weight = 2, the fitting results of $Rh@SiO_2$ are adopted from our previous work where identical conditions were used to collect the spectra.[3] [b] coordination number. [c] Debye-Waller parameter. [d] energy correction factor. [e] interatomic distance.

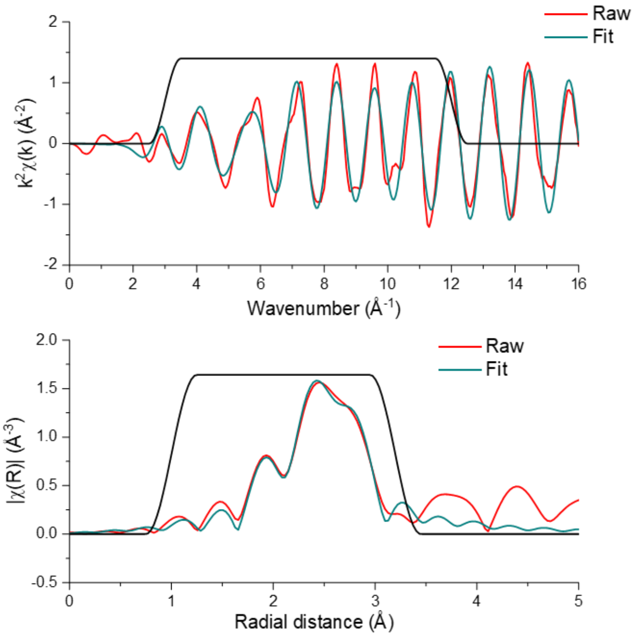

Figure S53. EXAFS fit for air exposed Ir@SiO_2. (top) K-space with raw (red) and fitted (grey) data. Window (blue) 3.0-12 Å^{-1}, k-weight = 2, Hanning window, dk = 1; (bottom) R-space with raw (red) and fitted (grey) data. Window (blue) 1-3. Å, k-weight = 2, Hanning window, dk = 0.5. Fit summarized in Table S7.

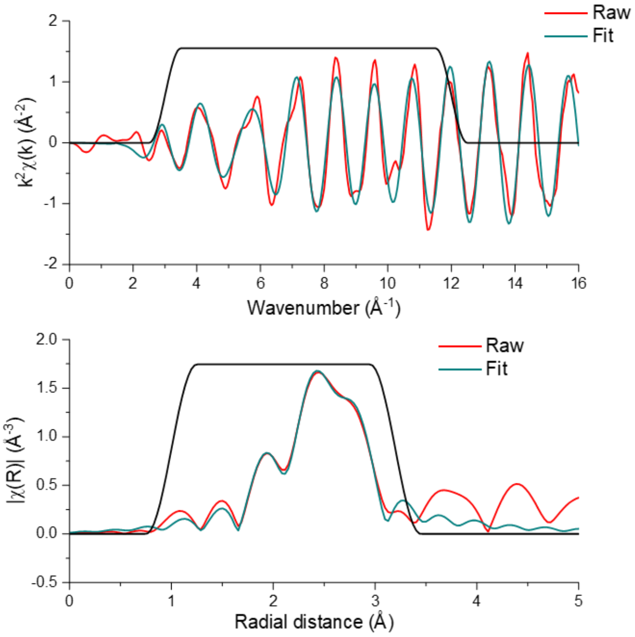

Figure S54. EXAFS fit for H_2 reduced Ir@SiO_2. (top) K-space with raw (red) and fitted (grey) data. Window (blue) 3.0-12 Å^{-1}, k-weight = 2, Hanning window, dk = 1; (bottom) R-space with raw (red) and fitted (grey) data. Window (blue) 1-3. Å, k-weight = 2, Hanning window, dk = 0.5. Fit summarized in Table S7.

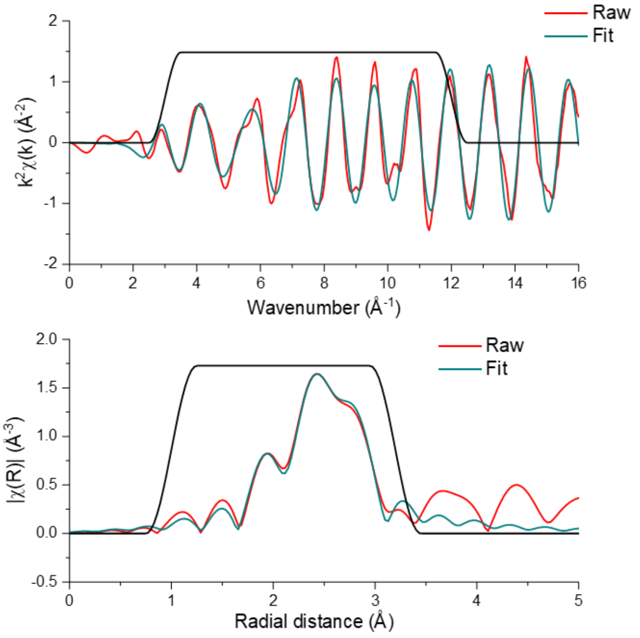

Figure S55. EXAFS fit for post CO_2 hydrogenation Ir@SiO_2. (top) K-space with raw (red) and fitted (grey) data. Window (blue) 3.0-12 $Å^{-1}$, k-weight = 2, Hanning window, dk = 1; (bottom) R-space with raw (red) and fitted (grey) data. Window (blue) 1-3. Å, k-weight = 2, Hanning window, dk = 0.5. Fit summarized in Table S7.

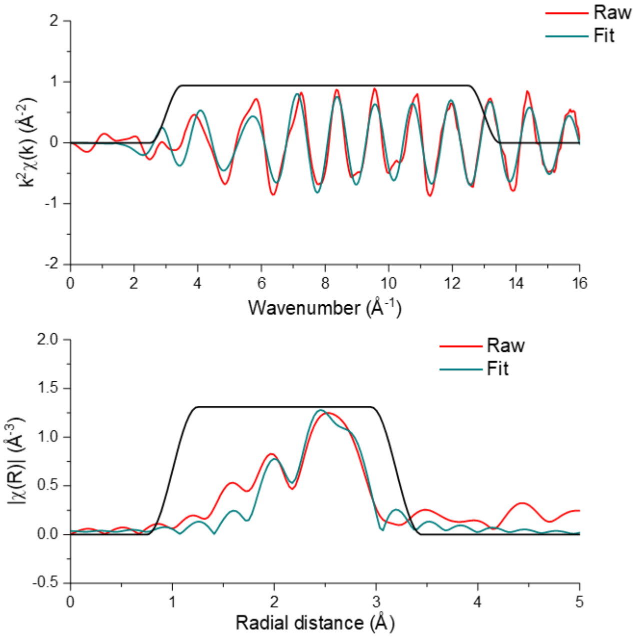

Figure S56. EXAFS fit for air exposed IrGa@SiO_2. (top) K-space with raw (red) and fitted (grey) data. Window (blue) 3.0-12 $Å^{-1}$, k-weight = 2, Hanning window, dk = 1; (bottom) R-space with raw (red) and fitted (grey) data. Window (blue) 1-3. Å, k-weight = 2, Hanning window, dk = 0.5. Fit summarized in Table S7.

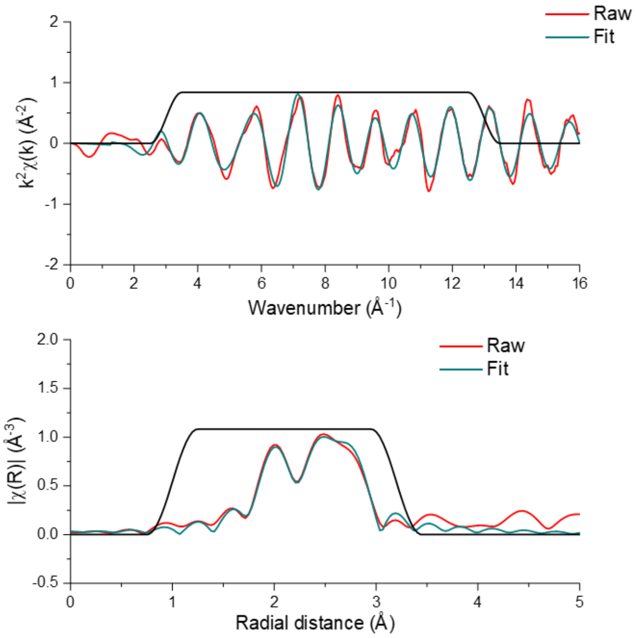

Figure S57. EXAFS fit for H_2 reduced IrGa@SiO_2. (top) K-space with raw (red) and fitted (grey) data. Window (blue) 3.0-12 Å^{-1}, k-weight = 2, Hanning window, dk = 1; (bottom) R-space with raw (red) and fitted (grey) data. Window (blue) 1-3. Å, k-weight = 2, Hanning window, dk = 0.5. Fit summarized in Table S7.

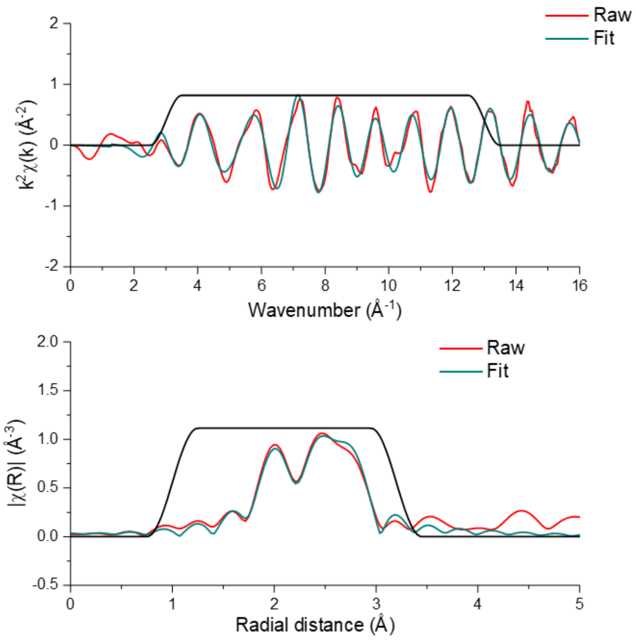

Figure S58. EXAFS fit for post CO_2 hydrogenation IrGa@SiO_2. (top) K-space with raw (red) and fitted (grey) data. Window (blue) 3.0-12 Å^{-1}, k-weight = 2, Hanning window, dk = 1; (bottom) R-space with raw (red) and fitted (grey) data. Window (blue) 1-3. Å, k-weight = 2, Hanning window, dk = 0.5. Fit summarized in Table S7.

Table S7. Summary of Ir L_3-edge fitting results of $IrSiO_2$ and IrGa@SiO_2 catalysts under different conditions.[a]

Catalyst	Conditions	Path	CN[b]	σ^2 (Å^2)[c]	ΔE (eV)[d]	R (Å)[e]
Ir@SiO_2	Air	Ir-Ir	10.4(0.8)	0.0035(0.0004)	7.5(0.8)	2.70(0.01)
	H_2 reduction	Ir-Ir	11.1(0.7)	0.0035(0.0004)	7.8(0.6)	2.70(0.01)
	Post CO_2 hydro.	Ir-Ir	11.1(0.7)	0.0037(0.0004)	7.7(0.6)	2.70(0.01)
IrGa@SiO_2	Air	Ir-Ir	9.8(1.4)	0.0051(0.0006)	7.1(1.7)	2.70(0.01)
	H_2 reduction	Ir-Ga	0.9(0.2)	0.0055(0.0005)	6.2(0.9)	2.49(0.01)
		Ir-Ir	9.2(0.9)	0.0055(0.0005)	6.2(0.9)	2.70(0.01)
	Post CO_2 hydro.	Ir-Ga	0.8(0.2)	0.0055(0.0006)	6.1(0.9)	2.49(0.02)
		Ir-Ir	9.3(0.9)	0.0055(0.0006)	6.1(0.9)	2.70(0.01)

[a] $3.0 < k < 12$ for Ir@SiO_2, $3.0 < k < 13$ for IrGa@SiO_2; S_o^2 was fixed as 0.75 (from $IrCl_3$ reference); $1 < R < 3.2$; k-weight = 2. [b] coordination number. [c] Debye-Waller parameter. [d] energy correction factor. [e] interatomic distance.

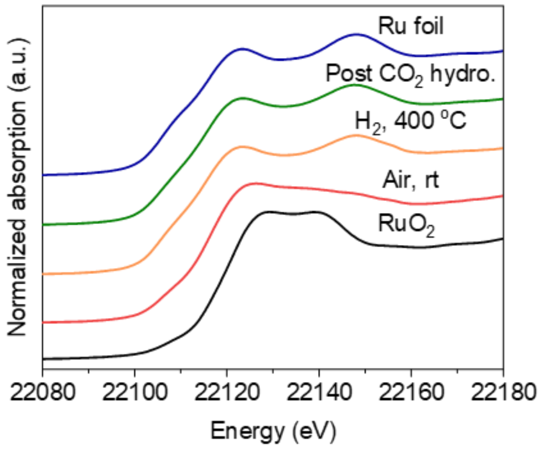

Figure S59. ***In situ* XAS results.** Ru K-edge XANES spectra for Ru@SiO_2 catalyst under different conditions and reference samples

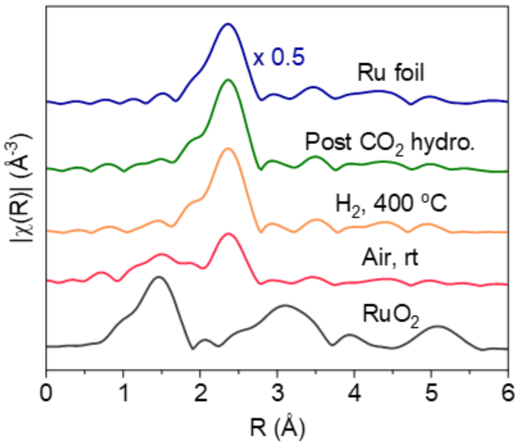

Figure S60. ***In situ* XAS results.** *In situ* Ru K-edge of k^2-weighted Fourier transforms of EXAFS spectra for Ru@SiO_2 catalyst under different conditions and reference samples.

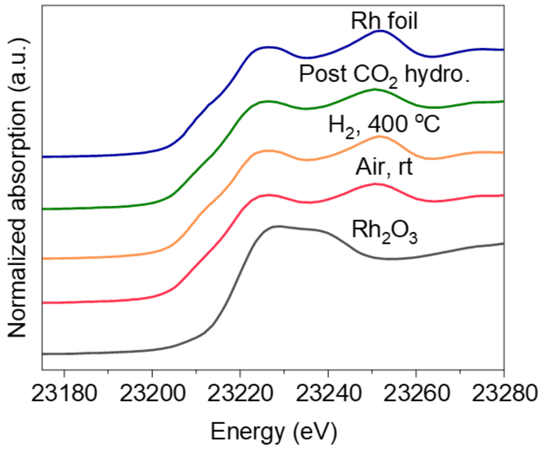

Figure S61. ***In situ* XAS results.** Rh K-edge XANES spectra for Rh@SiO_2 catalyst under different conditions and reference samples. The XAS results adapted from our previous work where identical conditions were used to collect the spectra.[3]

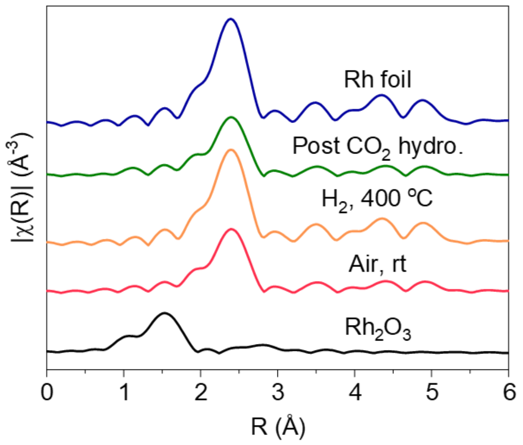

Figure S62. *In situ* XAS results. *In situ* Rh K-edge of k^2-weighted Fourier transforms of EXAFS spectra for Rh@SiO_2 catalyst under different conditions and reference samples. The XAS results adapted from our previous work where identical conditions were used to collect the spectra.[3]

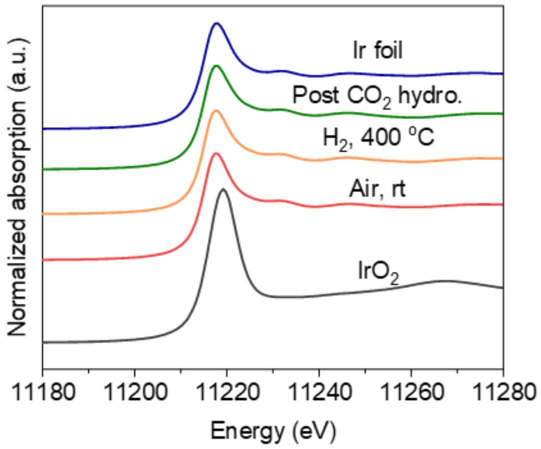

Figure S63. ***In situ* XAS results.** *In situ* Ir L_3-edge XANES spectra for Ir@SiO_2 catalyst under different conditions and reference samples

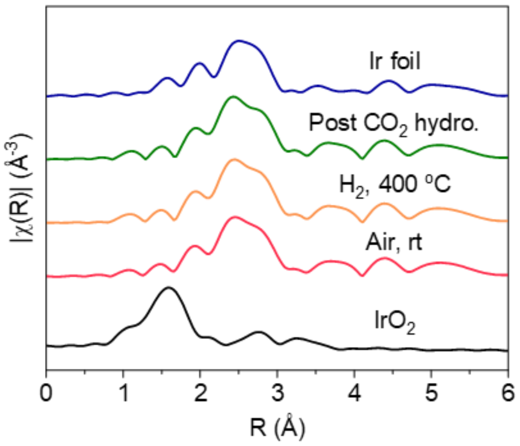

Figure S64. ***In situ* XAS results.** *In situ* Ir L_3-edge of k^2-weighted Fourier transforms of EXAFS spectra for Ir@SiO_2 catalyst under different conditions and reference samples.

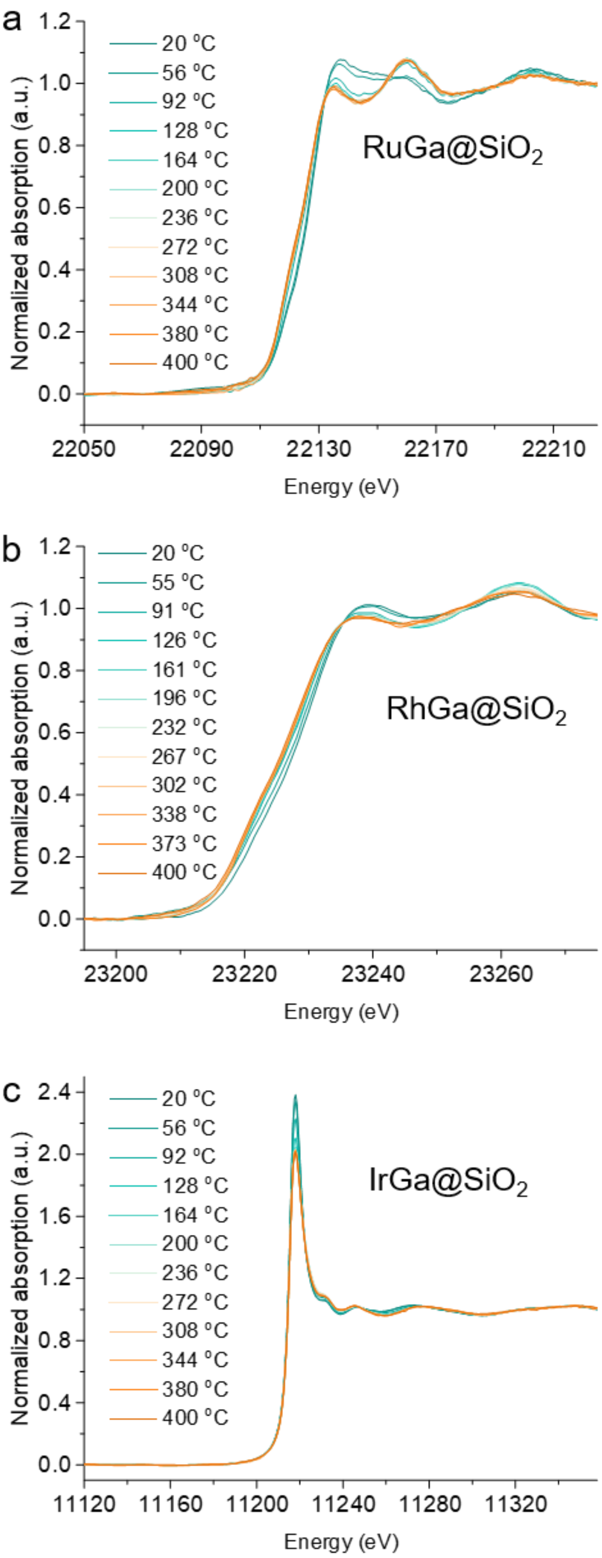

Figure S65. ***In situ* XAS results.** *In situ* Ru K-edge, Rh K-edge and Ir L_3-edge XANES spectra for RuGa@SiO_2 (a), RhGa@SiO2 (b) and IrGa@SiO_2 catalysts respectively collected during H_2 temperature programmed reduction.

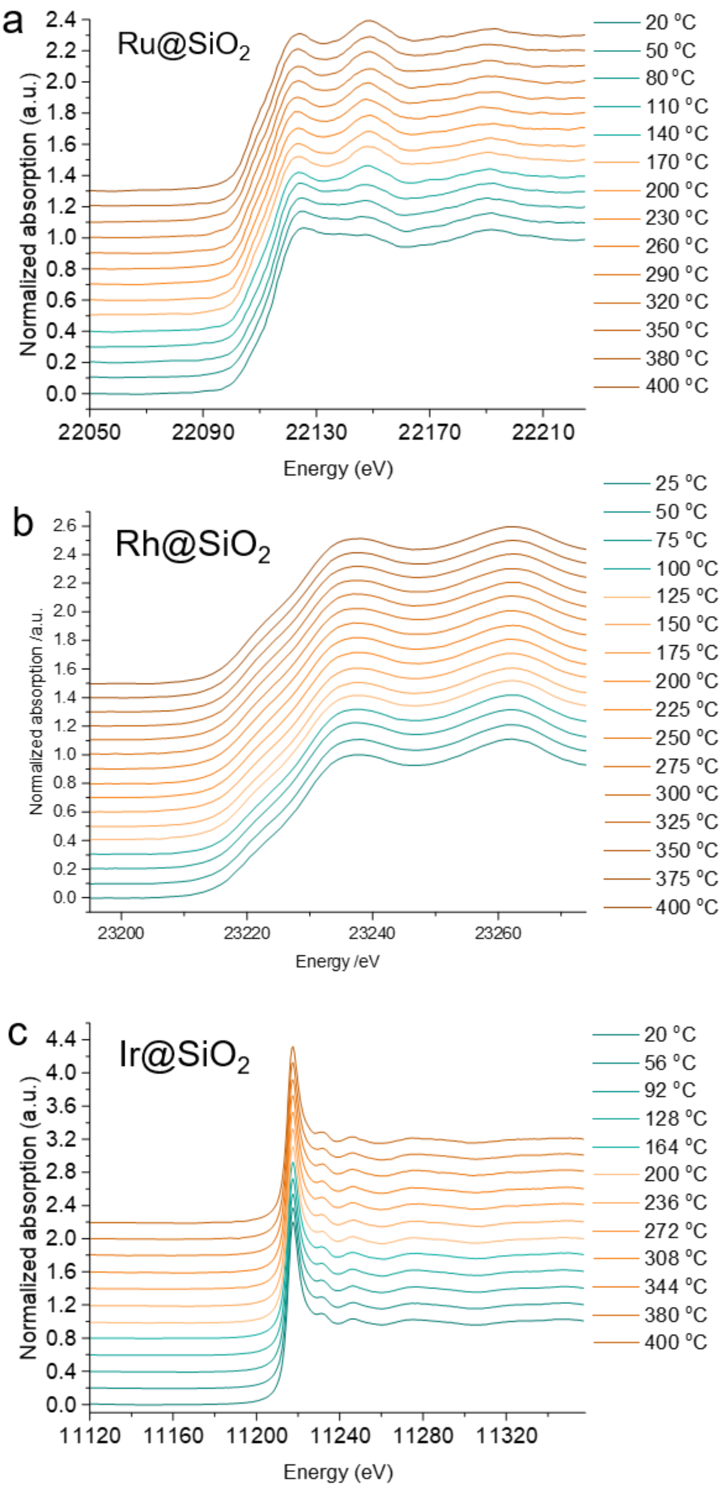

Figure S66. ***In situ* XAS results.** *In situ* Ru K-edge, Rh K-edge (adapted from our previous work where identical conditions were used to collect the spectra [3]) and Ir L_3-edge XANES spectra for (a) Ru@SiO_2, (b) Rh@SiO_2 and (c) Ir@SiO_2 catalysts respectively collected during H_2 temperature programmed reduction.

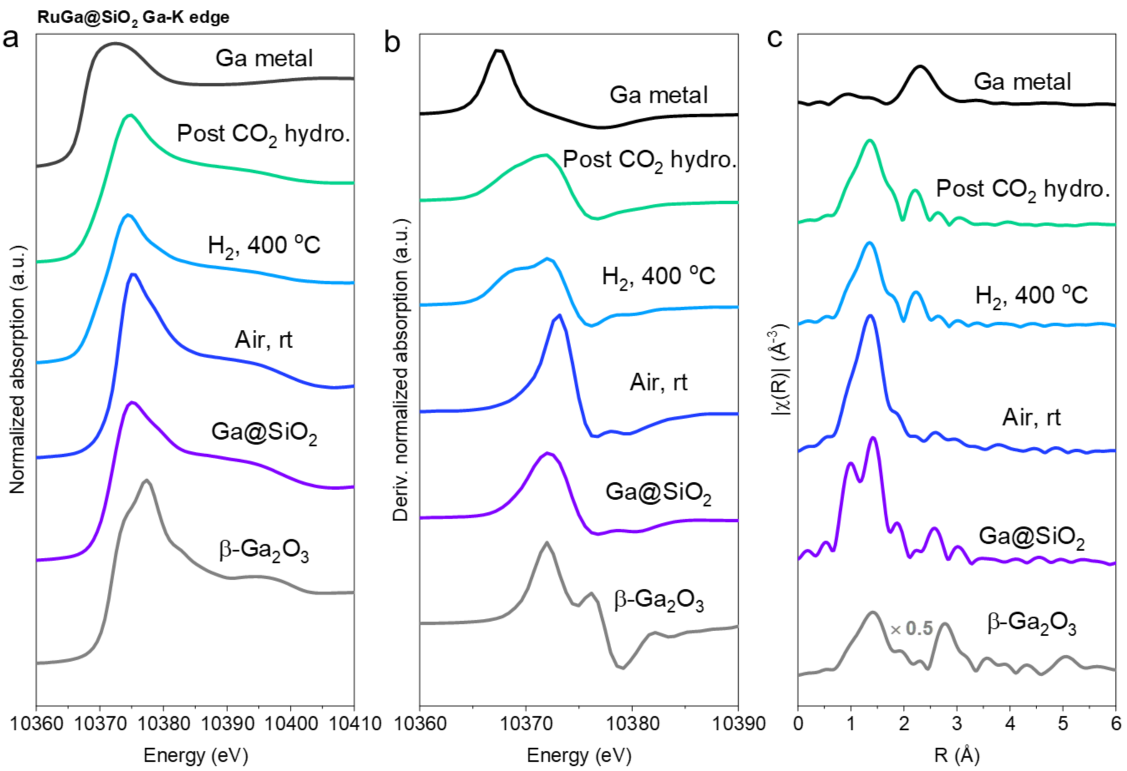

Figure S67. *In situ* XAS results at Ga K-edge of RuGa@SiO₂. (a) XANES spectra collected under different conditions and the related references, (b) corresponding first derivative spectra, (c) corresponding k^2-weighted Fourier transforms of EXAFS spectra.

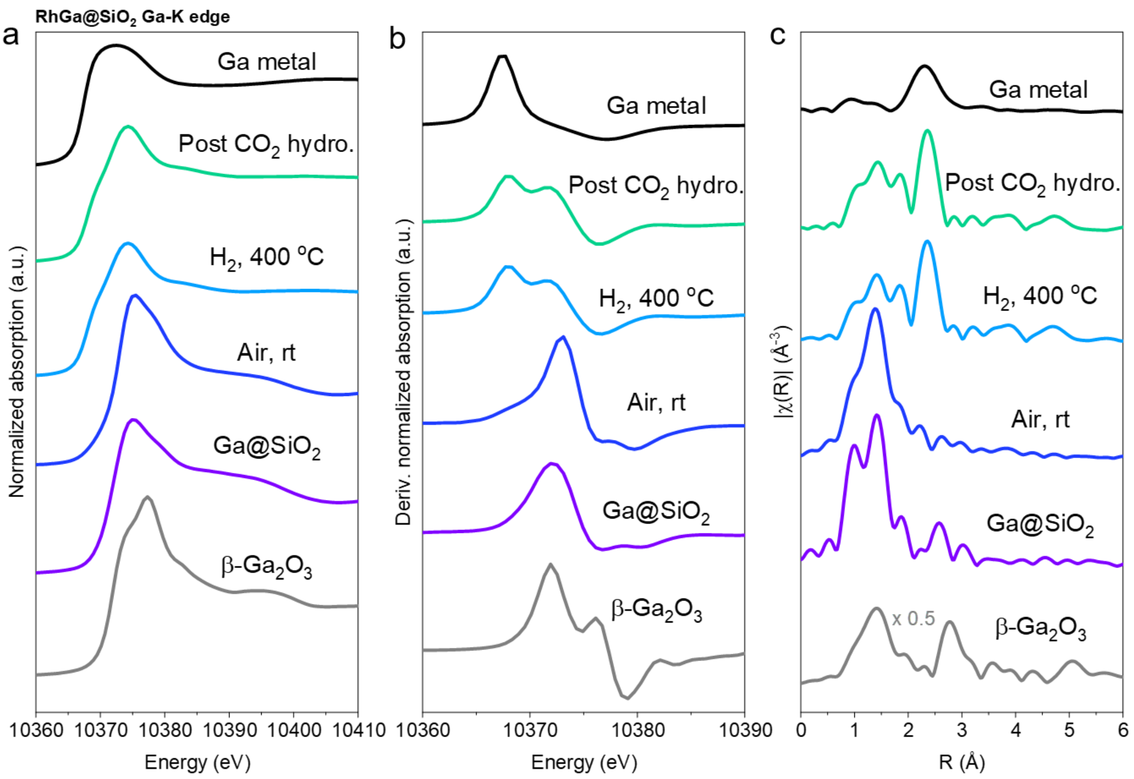

Figure S68. *In situ* XAS results at Ga K-edge of RhGa@SiO₂. (a) XANES spectra collected under different conditions and the related references, (b) corresponding first derivative spectra, (c) corresponding k^2-weighted Fourier transforms of EXAFS spectra.

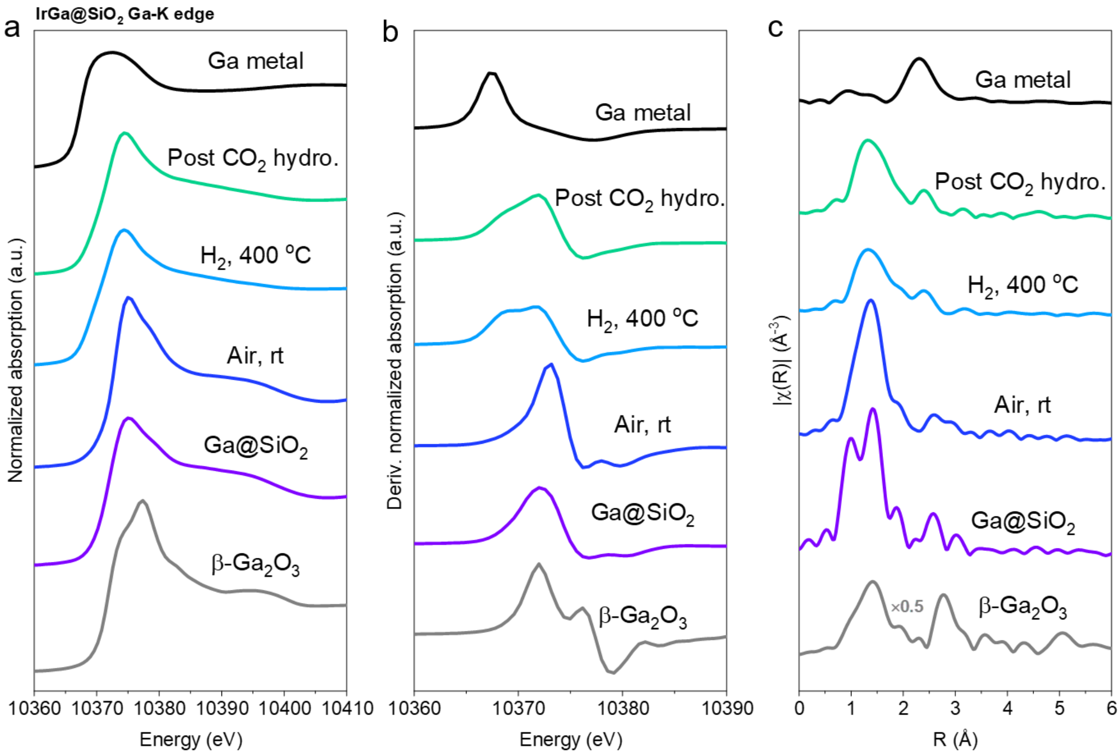

Figure S69. ***In situ*** **XAS results at Ga K-edge of IrGa@SiO2.** (a) XANES spectra collected under different conditions and the related references, (b) corresponding first derivative spectra, (c) corresponding k^2-weighted Fourier transforms of EXAFS spectra.

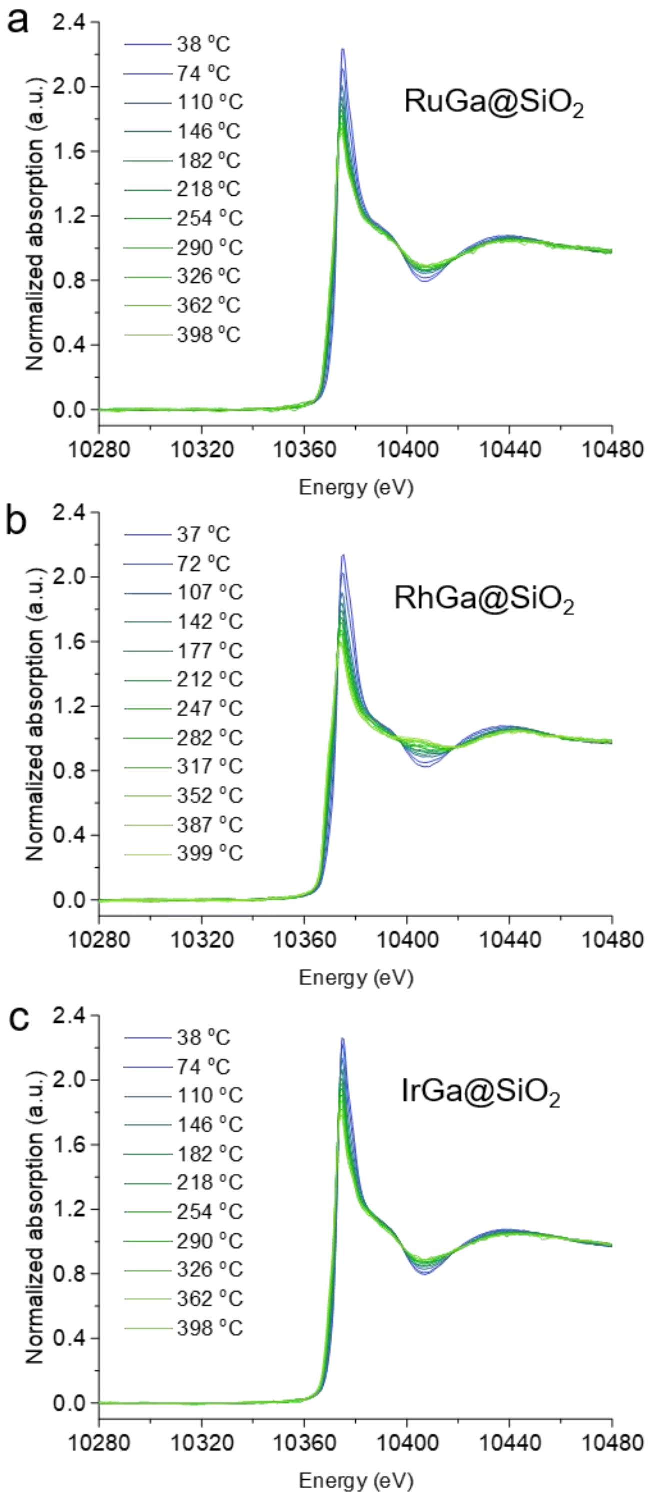

Figure S70. ***In situ* XAS results.** *In situ* Ga K-edge XANES spectra for RuGa@SiO_2 (a), RhGa@SiO_2 (b) and IrGa@SiO_2 catalysts collected during H_2 temperature programmed reduction.

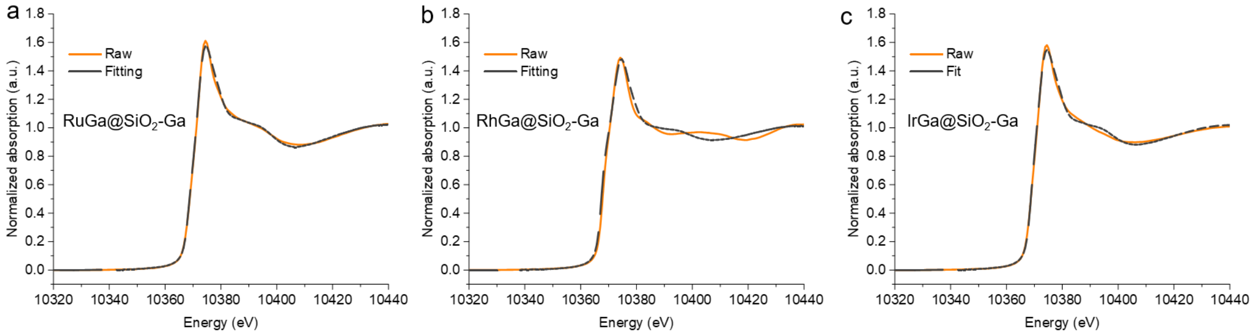

Figure S71. Linear combination fitting of Ga K-edge XANES after H_2 reduction. (a) RuGa@SiO_2, (b) RhGa@SiO_2, (c) IrGa@SiO_2. The Ga^{III}@SiO_2 and Ga foil are used as references for the LCF.

Table S8. Summary of LCF results and composition of *M*Ga alloy nanoparticles for Ga-promoted *M*Ga@SiO_2 catalysts after H_2 reduction.[a]

Catalyst	Component	Proportion (%)	Average *M*/Ga ratio
RuGa@SiO_2	Ga^{III}	63	3.0:1
	Ga	37	
RhGa@SiO_2	Ga^{III}	39	1.6:1
	Ga	61	
IrGa@SiO_2	Ga^{III}	55	2.4:1
	Ga	45	

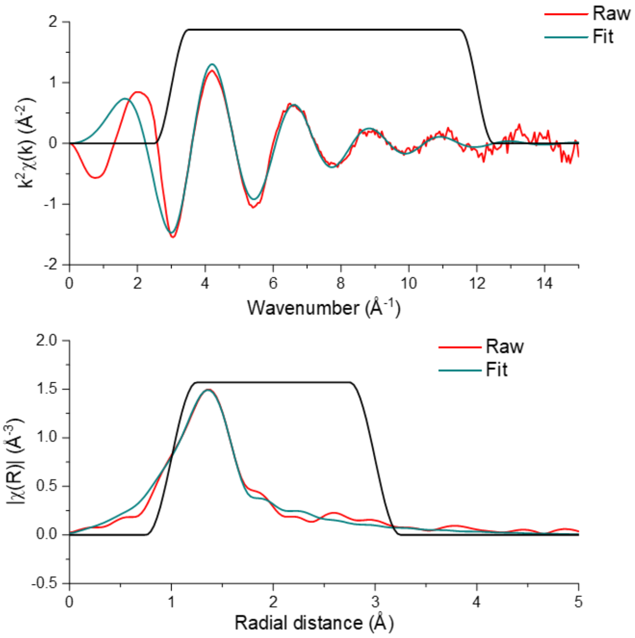

Figure S72. EXAFS fit for air exposed RuGa@SiO$_2$ at Ga K-edge. (top) K-space with raw (red) and fitted (grey) data. Window (blue) 3.0-12 Å^{-1}, k-weight = 2, Hanning window, dk = 1; (bottom) R-space with raw (red) and fitted (grey) data. Window (blue) 1-3. Å, k-weight = 2, Hanning window, dk = 0.5. Fit summarized in Table S9.

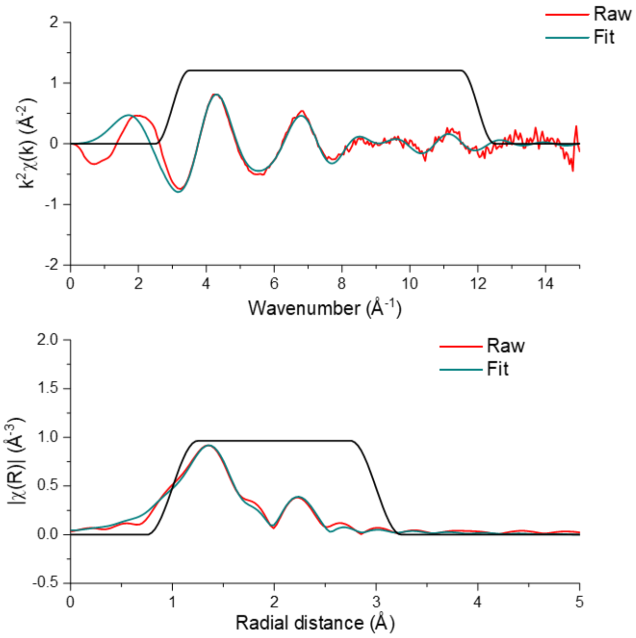

Figure S73. EXAFS fit for H$_2$ reduced RuGa@SiO$_2$ at Ga K-edge. (top) K-space with raw (red) and fitted (grey) data. Window (blue) 3.0-12 Å^{-1}, k-weight = 2, Hanning window, dk = 1; (bottom) R-space with raw (red) and fitted (grey) data. Window (blue) 1-3. Å, k-weight = 2, Hanning window, dk = 0.5. Fit summarized in Table S9.

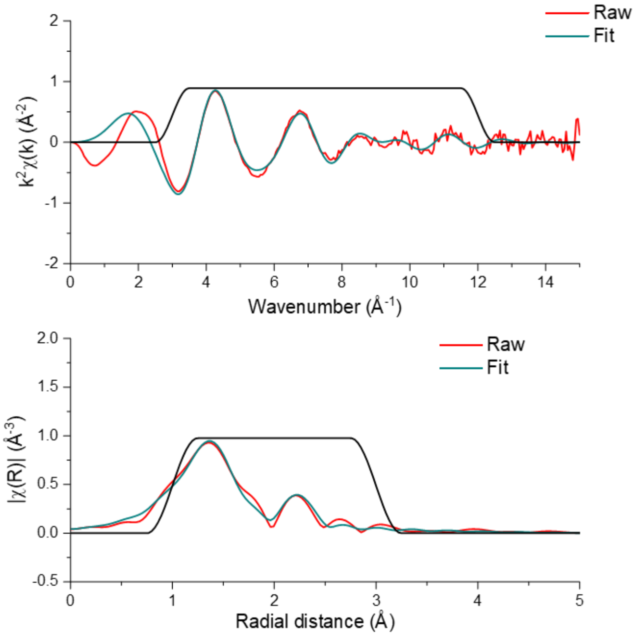

Figure S74. EXAFS fit for post CO_2 hydrogenation RuGa@SiO_2 at Ga K-edge. (top) K-space with raw (red) and fitted (grey) data. Window (blue) 3.0-12 $Å^{-1}$, k-weight = 2, Hanning window, dk = 1; (bottom) R-space with raw (red) and fitted (grey) data. Window (blue) 1-3. Å, k-weight = 2, Hanning window, dk = 0.5. Fit summarized in Table S9.

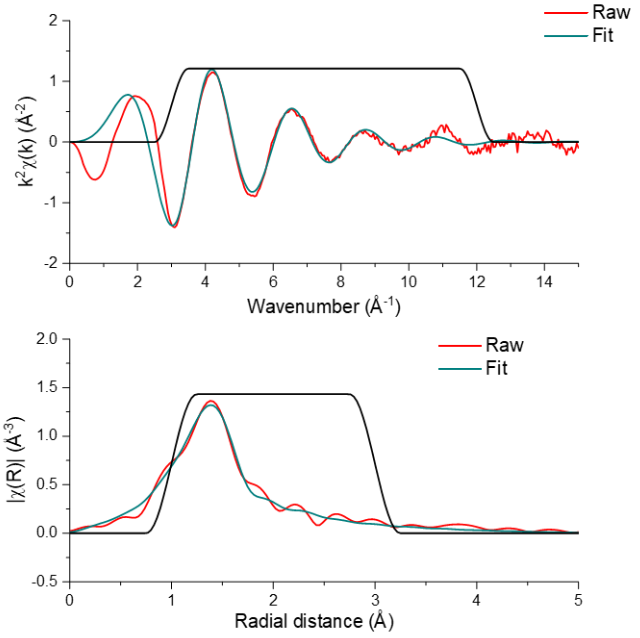

Figure S75. EXAFS fit for air exposed RhGa@SiO_2 at Ga K-edge. (top) K-space with raw (red) and fitted (grey) data. Window (blue) 3.0-12 $Å^{-1}$, k-weight = 2, Hanning window, dk = 1; (bottom) R-space with raw (red) and fitted (grey) data. Window (blue) 1-3. Å, k-weight = 2, Hanning window, dk = 0.5. Fit summarized in Table S9.

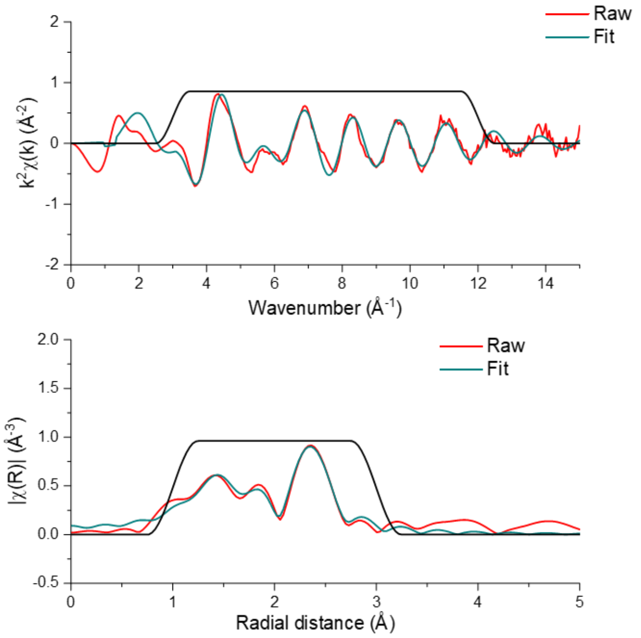

Figure S76. EXAFS fit for H_2 reduced RhGa@SiO_2 at Ga K-edge. (top) K-space with raw (red) and fitted (grey) data. Window (blue) 3.0-12 $Å^{-1}$, k-weight = 2, Hanning window, dk = 1; (bottom) R-space with raw (red) and fitted (grey) data. Window (blue) 1-3. Å, k-weight = 2, Hanning window, dk = 0.5. Fit summarized in Table S9.

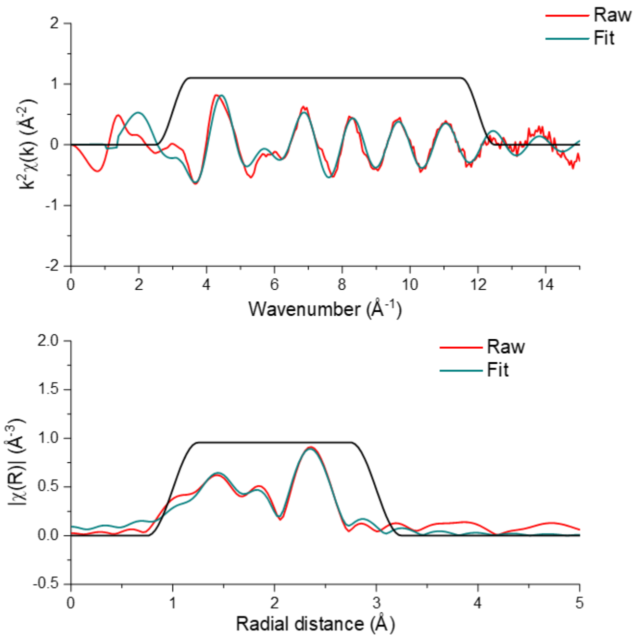

Figure S77. EXAFS fit for post CO_2 hydrogenation RhGa@SiO_2 at Ga K-edge. (top) K-space with raw (red) and fitted (grey) data. Window (blue) 3.0-12 $Å^{-1}$, k-weight = 2, Hanning window, dk = 1; (bottom) R-space with raw (red) and fitted (grey) data. Window (blue) 1-3. Å, k-weight = 2, Hanning window, dk = 0.5. Fit summarized in Table S9.

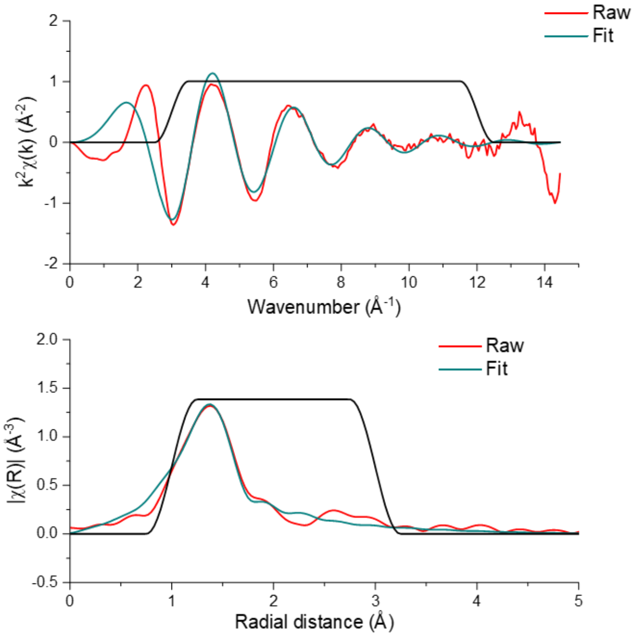

Figure S78. EXAFS fit for air exposed IrGa@SiO_2 at Ga K-edge. (top) K-space with raw (red) and fitted (grey) data. Window (blue) 3.0-12 Å^{-1}, k-weight = 2, Hanning window, dk = 1; (bottom) R-space with raw (red) and fitted (grey) data. Window (blue) 1-3. Å, k-weight = 2, Hanning window, dk = 0.5. Fit summarized in Table S9.

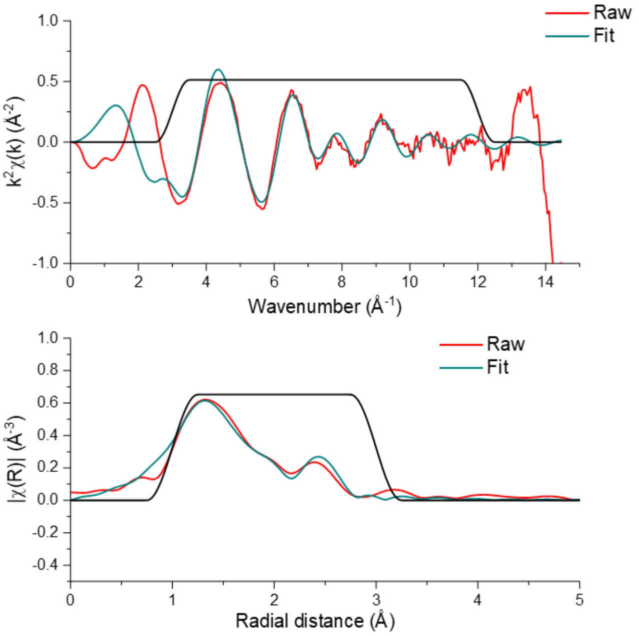

Figure S79. EXAFS fit for H_2 reduced IrGa@SiO_2 at Ga K-edge. (top) K-space with raw (red) and fitted (grey) data. Window (blue) 3.0-12 Å^{-1}, k-weight = 2, Hanning window, dk = 1; (bottom) R-space with raw (red) and fitted (grey) data. Window (blue) 1-3. Å, k-weight = 2, Hanning window, dk = 0.5. Fit summarized in Table S9.

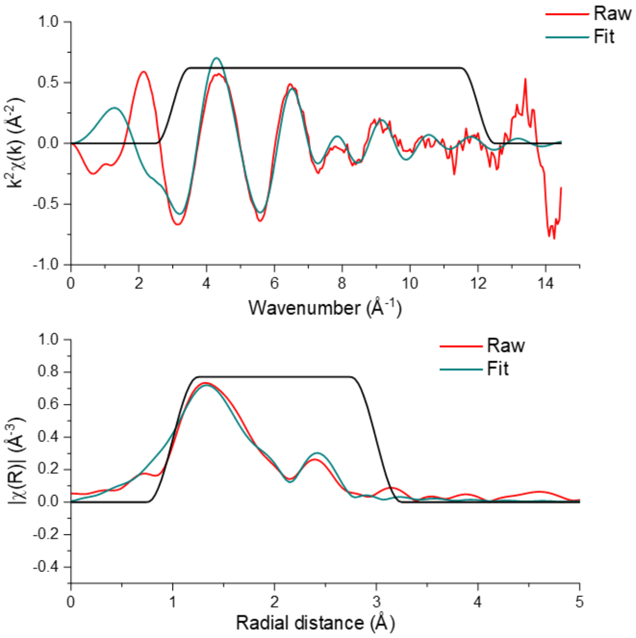

Figure S80. EXAFS fit for post CO_2 hydrogenation IrGa@SiO_2 at Ga K-edge. (top) K-space with raw (red) and fitted (grey) data. Window (blue) 3.0-12 $Å^{-1}$, k-weight = 2, Hanning window, dk = 1; (bottom) R-space with raw (red) and fitted (grey) data. Window (blue) 1-3. Å, k-weight = 2, Hanning window, dk = 0.5. Fit summarized in Table S9.

Table S9. Summary of Ga K-edge fitting results of RuGa@SiO_2, Rh Ga@SiO_2 and IrGa@SiO_2 catalysts under different conditions.[a]

Catalyst	Conditions	Path	CN[b]	σ^2 (Å^2)[c]	ΔE (eV)[d]	R (Å)[e]
RuGa@SiO_2	Air	Ga-O	5.6(0.5)	0.0085(0.0011)	-3.0(1.2)	1.82(0.01)
	H_2 reduction	Ga-O	2.8(0.3)	0.0073(0.0014)	-1.9(1.7)	1.80(0.01)
		Ga-Ru	0.8(0.2)	0.0073(0.0014)	-3.5(2.9)	2.50(0.02)
	Post CO_2 hydro.	Ga-O	3.0 (0.5)	0.0079(0.0019)	-1.3(2.6)	1.82(0.02)
		Ga-Ru	0.8(0.3)	0.0079(0.0019)	-6.0(5.1)	2.49(0.03)
RhGa@SiO_2	Air	Ga-O	5.2 (0.5)	0.0096(0.0014)	-0.6(1.4)	1.85(0.01)
	H_2 reduction	Ga-O	1.8(0.5)	0.0097(0.0051)	6.7(4.4)	1.83(0.04)
		Ga-Rh	2.3(0.5)	0.0063(0.0013)	3.7(1.3)	2.53(0.01)
	Post CO_2 hydro.	Ga-O	1.9(0.6)	0.0094(0.0057)	7.2(5.0)	1.85(0.04)
		Ga-Rh	2.1(0.5)	0.0057(0.0017)	6.1(0.9)	2.53(0.01)
IrGa@SiO_2	Air	Ga-O	4.6(0.6)	0.0078(0.0017)	-2.2(1.8)	1.83(0.01)
	H_2 reduction	Ga-O	2.9(0.8)	0.0114(0.0032)	-8.2(5.4)	1.77(0.03)
		Ga-Ir	3.1(1.3)	0.0114(0.0032)	-6.0(3.2)	2.49(0.02)
	Post CO_2 hydro.	Ga-O	3.5(1.1)	0.0119 (0.0038)	-7.4(5.5)	1.79(0.03)
		Ga-Ir	3.1(1.3)	0.0119 (0.0038)	-8.7(4.0)	2.49(0.03)

[a] $3.0 < k < 12$; S_o^2 was fixed as 0.83; $1 < R < 3$; k-weight = 2. [b] coordination number. [c] Debye-Waller parameter. [d] energy correction factor. [e] interatomic distance.

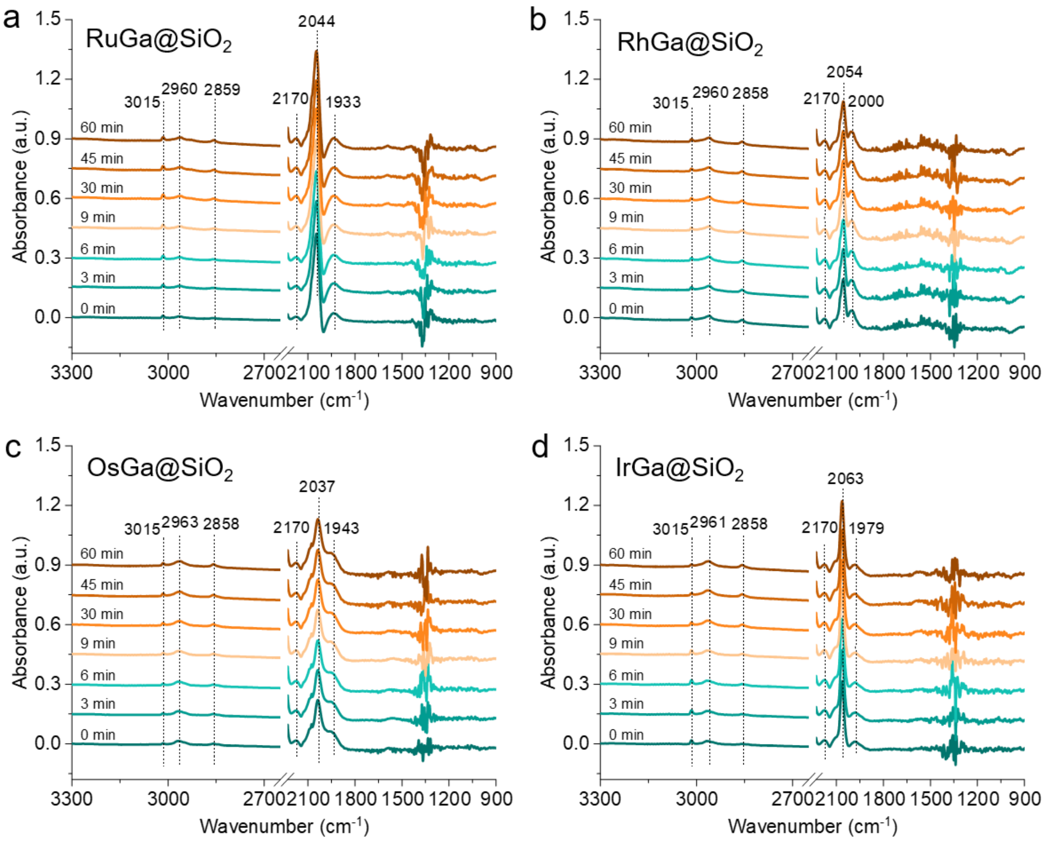

Figure S81. In situ DRIFTS. The spectra were collected on (a) RuGa@SiO_2, (b) RhGa@SiO_2, (c) OsGa@SiO_2 and (d) IrGa@SiO_2 under H_2/CO_2/Ar (3:1:1, 20 bar).

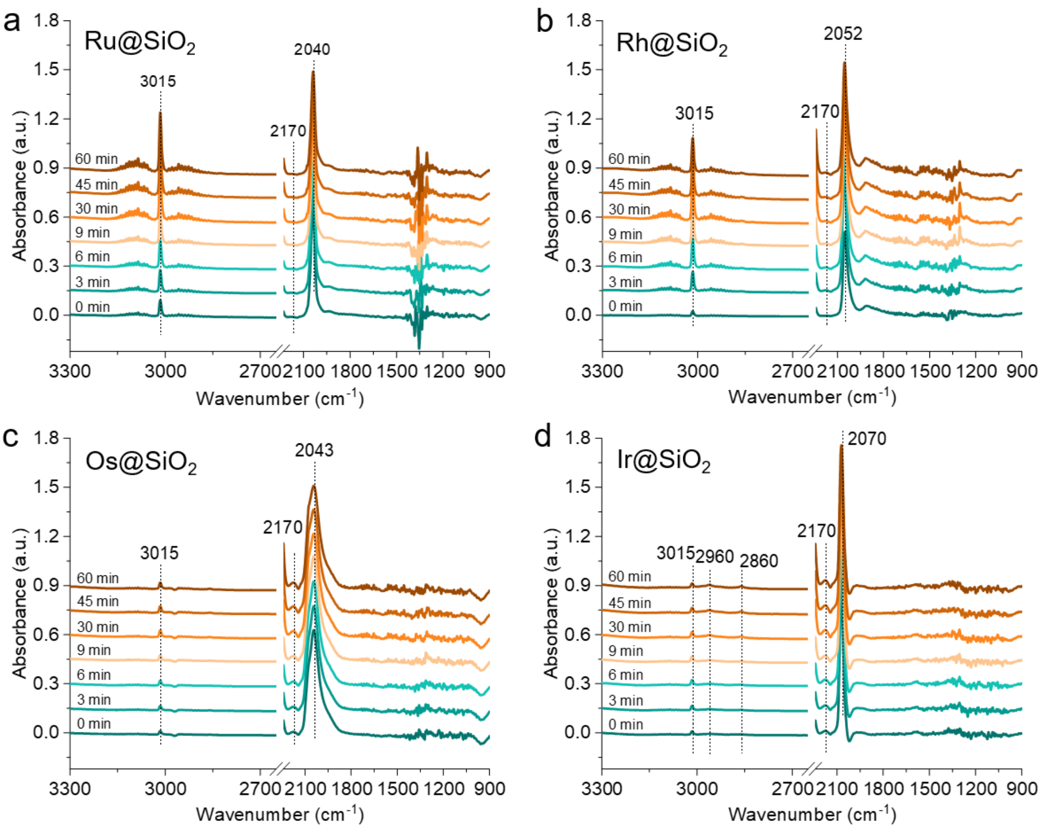

Figure S82. In situ DRIFTS. The spectra were collected on (a) Ru@SiO_2, (b) Rh@SiO_2, (c) Os@SiO_2 and (d) Ir@SiO_2 under H_2/CO_2/Ar (3:1:1, 20 bar).

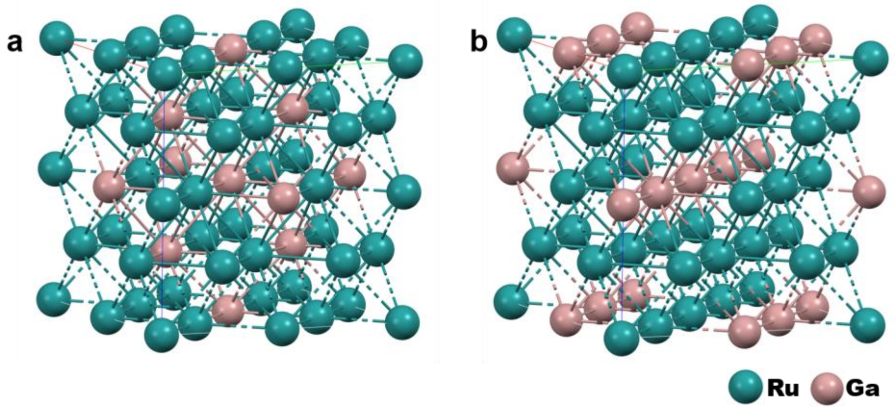

Figure S83. The optimized bulk structure of $Ru_{0.75}Ga_{0.25}$ with two different atomic arrangements. (a) Ga atoms are at the body-center, face-centers and (0.25/0.75, 0.5, 0.25/0.75); (b) Ga atoms are aligned along the [110] direction in the FCC structure.

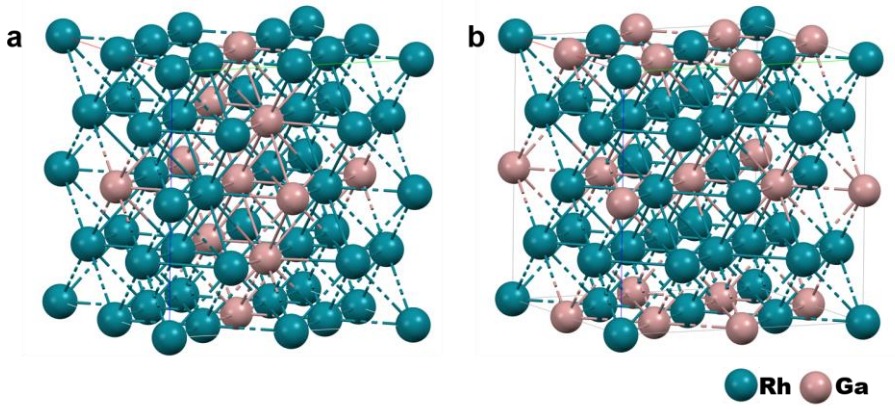

Figure S84. The optimized bulk structure of $Rh_{0.75}Ga_{0.25}$ with two different atomic arrangements. (a) Ga atoms are at the body-center, face-centers and (0.25/0.75, 0.5, 0.25/0.75); (b) Ga atoms are aligned along the [110] direction in the FCC structure.

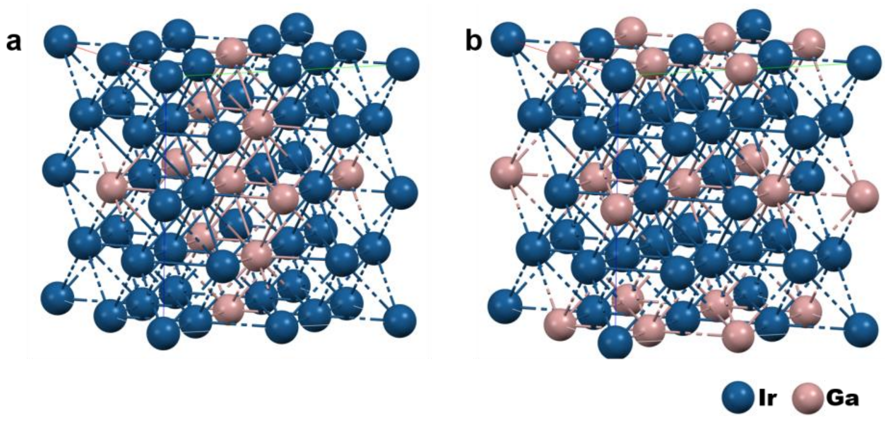

Figure S85. The optimized bulk structure of $Ir_{0.75}Ga_{0.25}$ with two different atomic arrangements. (a) Ga atoms are at the body-center, face-centers and (0.25/0.75, 0.5, 0.25/0.75); (b) Ga atoms are aligned along the [110] direction in the FCC structure.

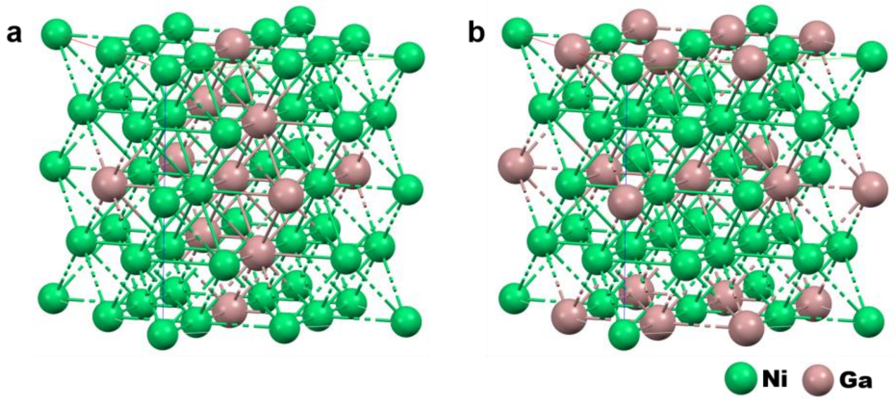

Figure S86. The optimized bulk structure of $Ni_{0.75}Ga_{0.25}$ with two different atomic arrangements. (a) Ga atoms are at the body-center, face-centers and (0.25/0.75, 0.5, 0.25/0.75); (b) Ga atoms are aligned along the [110] direction in the FCC structure.

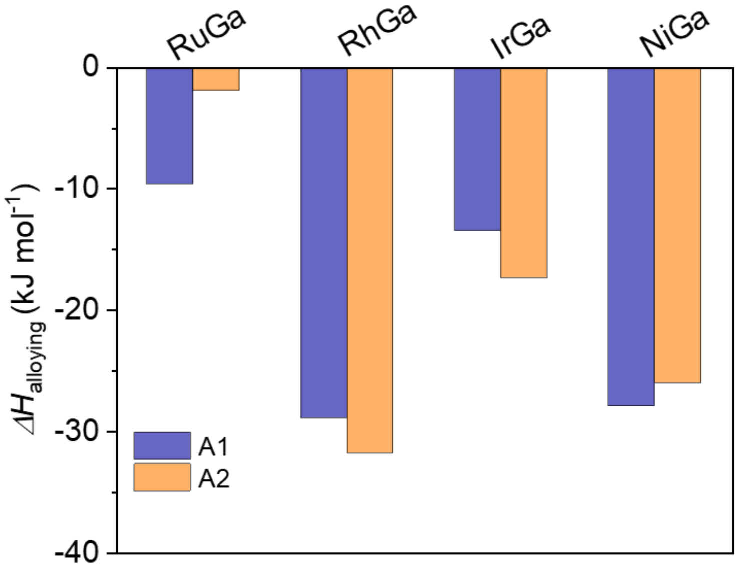

Figure S87. The calculated alloy formation enthalpy (*$\Delta H_{alloying}$*) of different *M*Ga (*M* = Ru, Rh, Ir and Ni) bimetallic systems based on FCC structure. A1 and A2 represent two different Ga arrangements within the alloy systems, as illustrated in Figures 82-85. A1 represents the structure with Ga atoms at the body-center, face-centers and (0.25/0.75, 0.5, 0.25/0.75) position. A2 represents the structure with Ga aligned along the [110] direction in the FCC structure.

Table S10. Summary of of alloy formation enthalpy over different *M*Ga (*M* = Ru, Rh, Ir and Ni) bimetallic system based on FCC structure.[a]

Metal	E_{bulk} (eV)	N_{atom}	$E_{per\ atom}$ (eV)	$\Delta H_{alloying}$ (eV)	$\Delta H_{alloying}$ (kJ/mol)
Ru	-312.52	32 Ru	-9.77	\	\
Rh	-253.56	32 Rh	-7.92	\	\
Ir	-306.31	32 Ir	-9.57	\	\
Ni	-188.04	32 Ni	-5.88	\	\
Ga_Orth	-102.48	32 Ga	-3.20	\	\
$Ru_{0.75}Ga_{0.25}$-P1	-263.35	24 Ru + 8 Ga	-8.23	-0.10	-9.65
$Ru_{0.75}Ga_{0.25}$-P2	-260.53	24 Ru + 8 Ga	-8.14	-0.02	-1.93
$Rh_{0.75}Ga_{0.25}$-P1	-225.31	24 Rh + 8 Ga	-7.04	-0.30	-28.95
$Rh_{0.75}Ga_{0.25}$-P2	-226.27	24 Rh + 8 Ga	-7.07	-0.33	-31.84
$Ir_{0.75}Ga_{0.25}$-P1	-259.87	24 Ir + 8 Ga	-8.12	-0.14	-13.51
$Ir_{0\cdot75}Ga_{0.25}$-P2	-261.03	24 Ir + 8 Ga	-8.16	-0.18	-17.37
$Ni_{0.75}Ga_{0.25}$-P1	-175.84	24 Ni + 8 Ga	-5.50	-0.29	-27.98
$Ni_{0.75}Ga_{0.2}5$-P2	-175.19	24 Ni + 8 Ga	-5.47	-0.27	-26.05

[a] $\Delta H_{alloying} = E_{Bulk}(M_{0.75}Ga_{0.25}) - 0.75 * E_{Bulk}(M) - 0.25 * E_{Bulk}(Ga)$

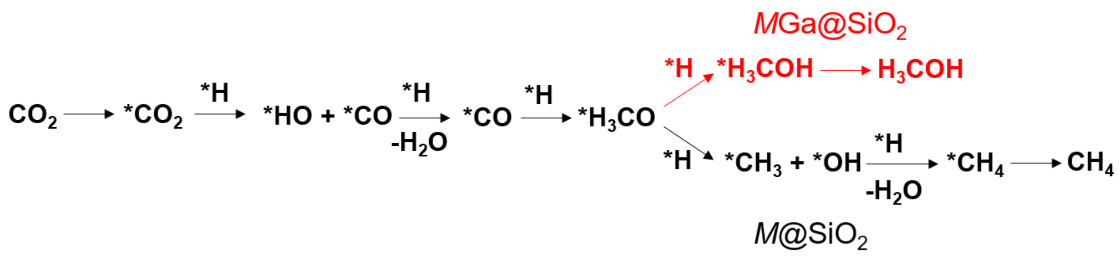

Figure S88. The possible reaction pathway over bimetallic *M*Ga@SiO_2 and monometallic *M*@SiO_2 for CO_2 hydrogenation.

Note: Previous studies propose two possible reaction pathway for CO_2 hydrogenation to methanol: One is through formate (HCOO*) intermediate without CO formation; the other involves reverse water gas shift (RWGS) route to convert CO_2 to CO and then CO is further hydrogenated to methanol.[22] Our *in situ DRIFTS* experiments do not monitor any formate species but adsorbed CO* on all the catalysts, which allows us to deem that the later reaction pathway dominates in our system. Furthermore, the methoxy (CH_3O*) species, a key intermediate for methanol formation, are observed on all the four *M*Ga systems. We therefore propose a possible reaction pathway over bimetallic *M*Ga@SiO_2 and monometallic *M*@SiO_2 catalysts: The CO_2 is first converted to CO intermediate via RWGS route, then CO is further hydrogenated to methanol via methoxy species over *M*Ga@SiO_2. However, the methoxy species will be easily overhydrogenated to CH_4 over the *M*@SiO_2 catalyst in the absence of Ga. Therefore, we evaluate the dissociative adsorption energy of C-O bond of methoxy on the monometallic *M* and bimetallic *M*Ga systems in the presence of adsorbed H* as the simplest possible descriptor to help us understand the role of *M*Ga alloy under CO_2 hydrogenation in promoting the methanol formation while suppressing the methanation reaction.

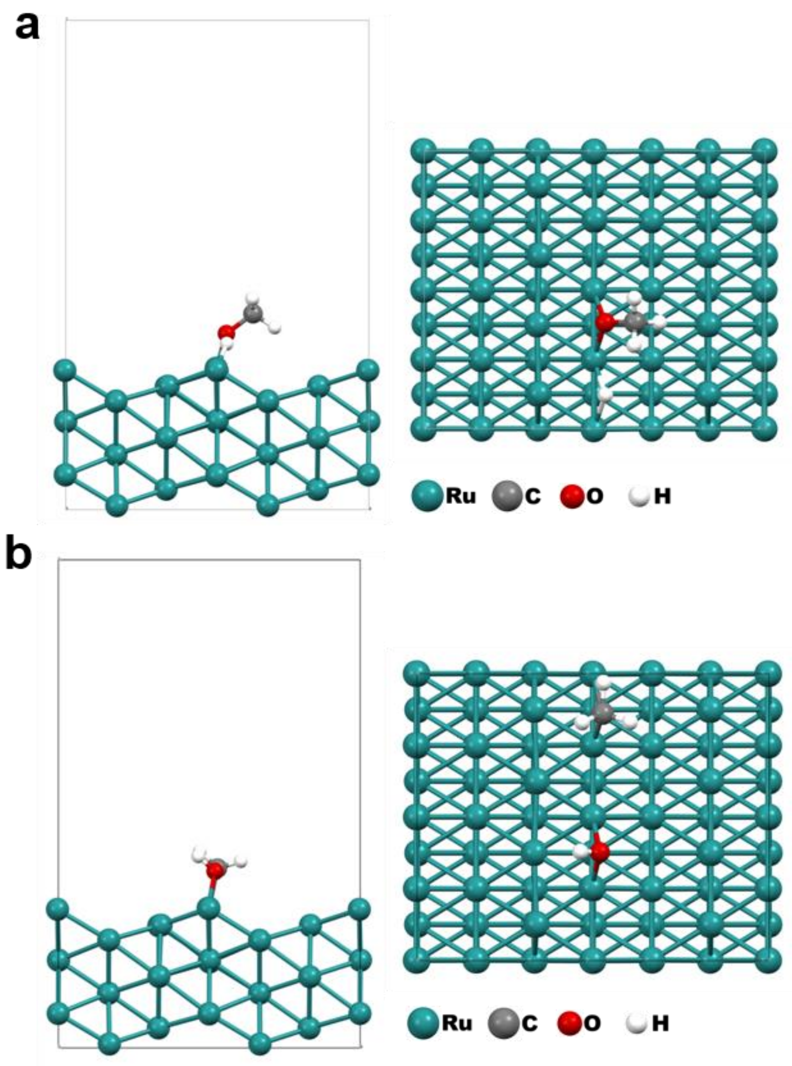

Figure S89. The optimized structure of adsorbed CH_3O^* and H* (a) and adsorbed CH_3^* and OH* (b) on monometallic Ru {211} facet models.

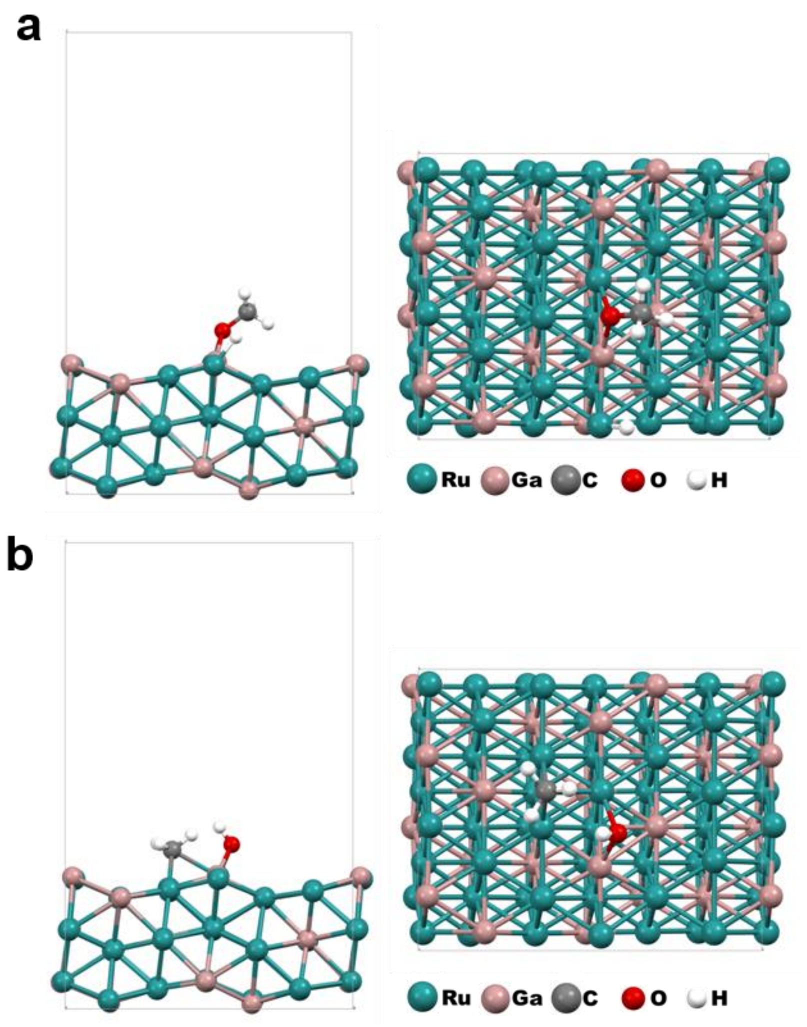

Figure S90. The optimized structure of adsorbed CH_3O* and H* (a) and adsorbed CH_3* and OH* (b) on bimetallic RuGa {211} facet models.

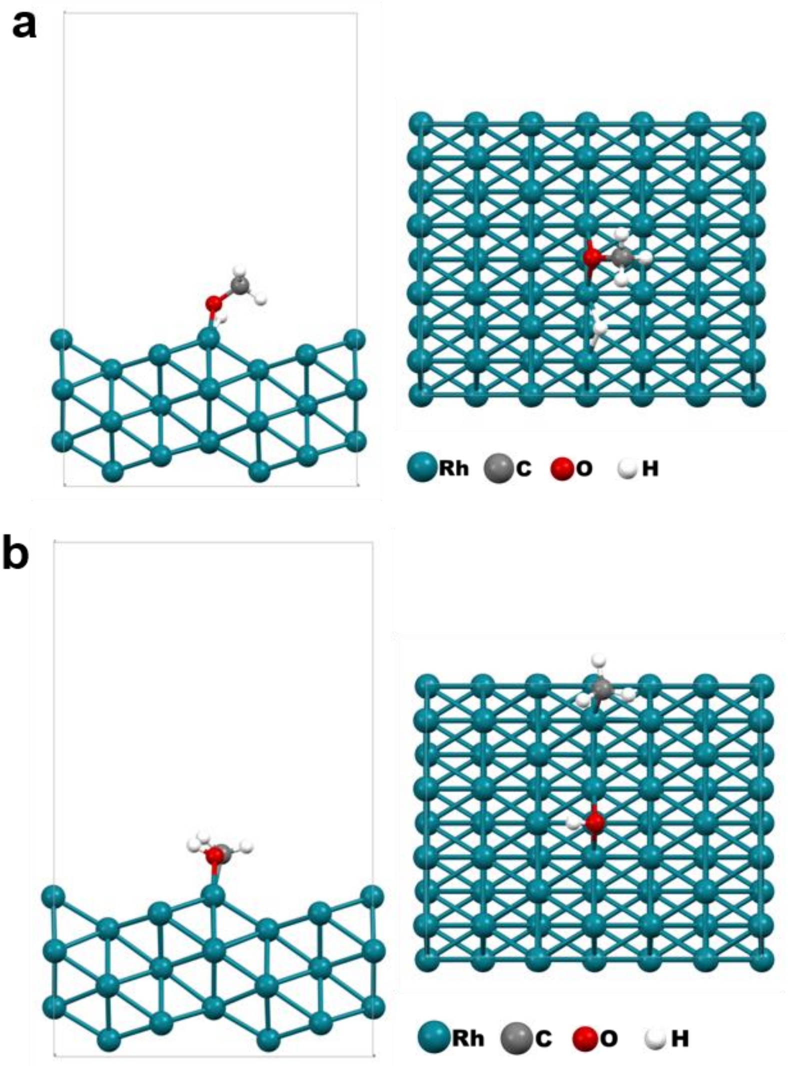

Figure S91. The optimized structure of adsorbed CH_3O* and H* (a) and adsorbed CH_3* and OH* (b) on monometallic Rh {211} facet models.

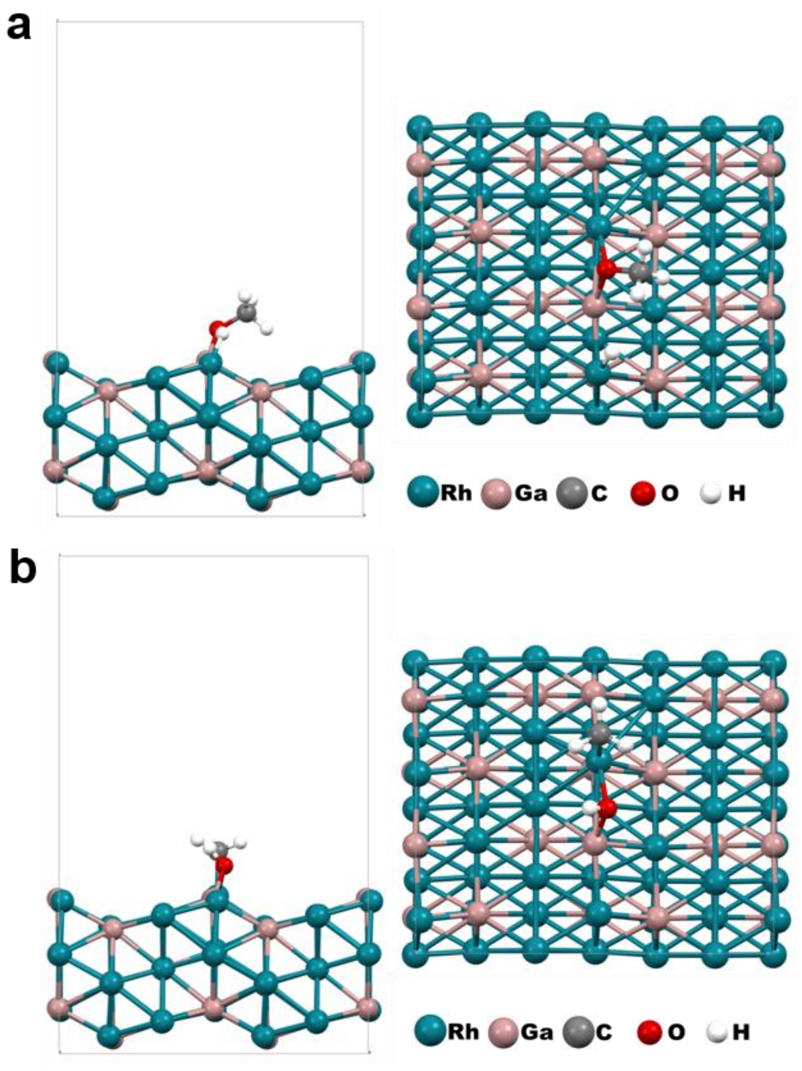

Figure S92. The optimized structure of adsorbed CH_3O^* and H^* (a) and adsorbed CH_3^* and OH^* (b) on bimetallic RhGa {211} facet models.

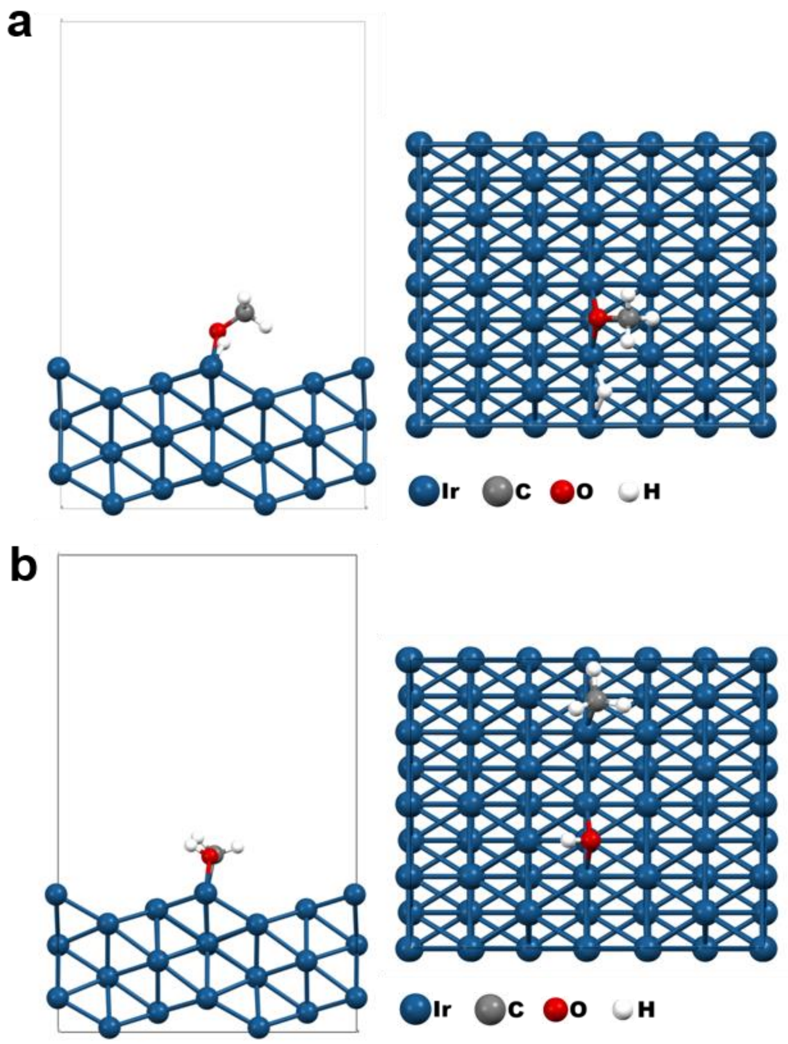

Figure S93. The optimized structure of adsorbed CH_3O* and H* (a) and adsorbed CH_3* and OH* (b) on monometallic Ir {211} facet models.

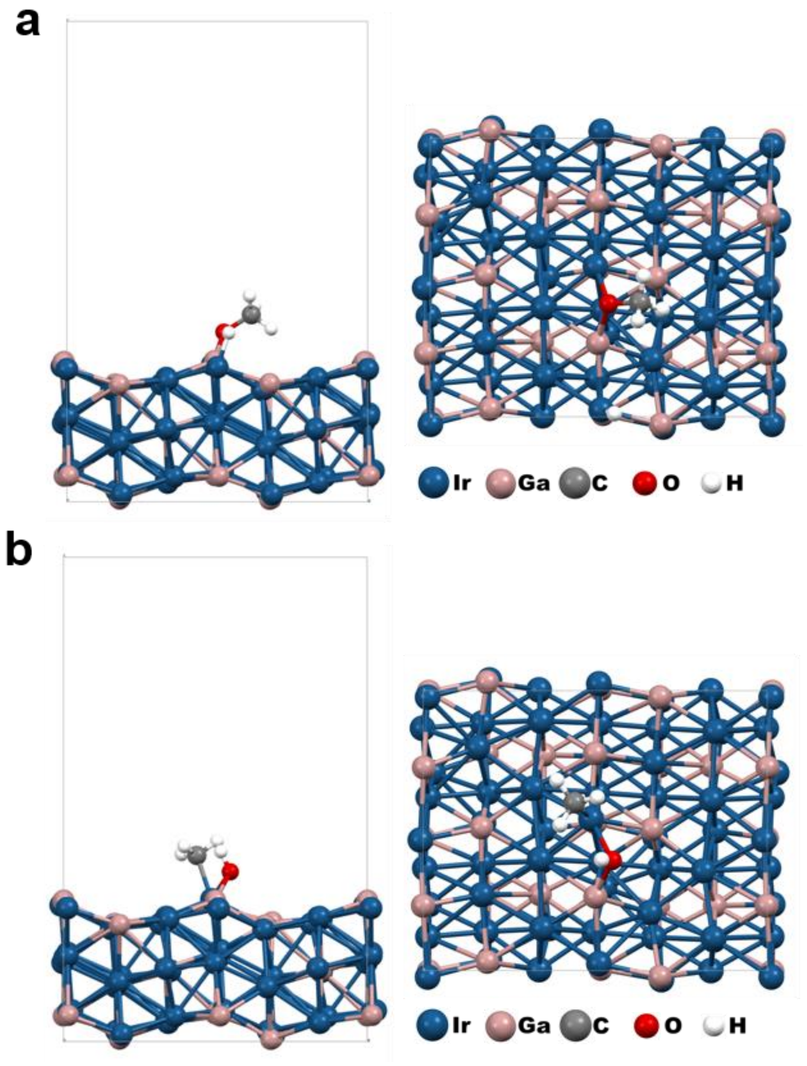

Figure S94. The optimized structure of adsorbed CH_3O* and H* (a) and adsorbed CH_3* and OH* (b) on bimetallic IrGa {211} facet models.

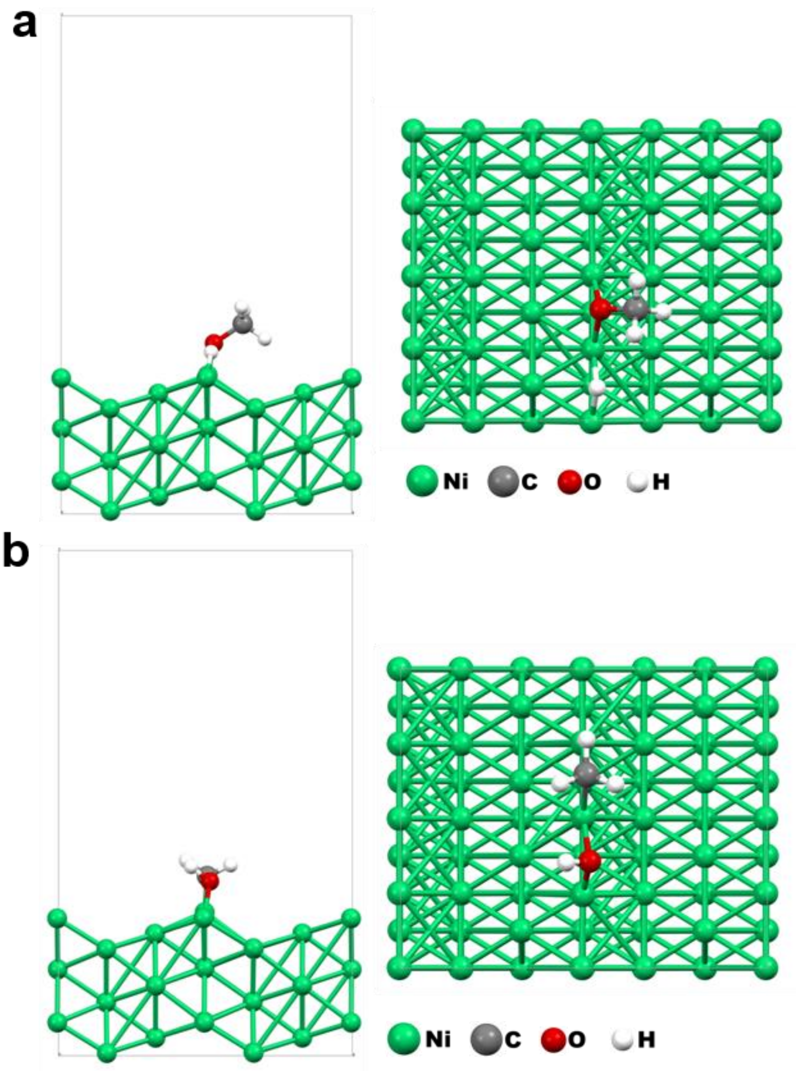

Figure S95. The optimized structure of adsorbed CH_3O* and H* (a) and adsorbed CH_3* and OH* (b) on monometallic Ni {211} facet models.

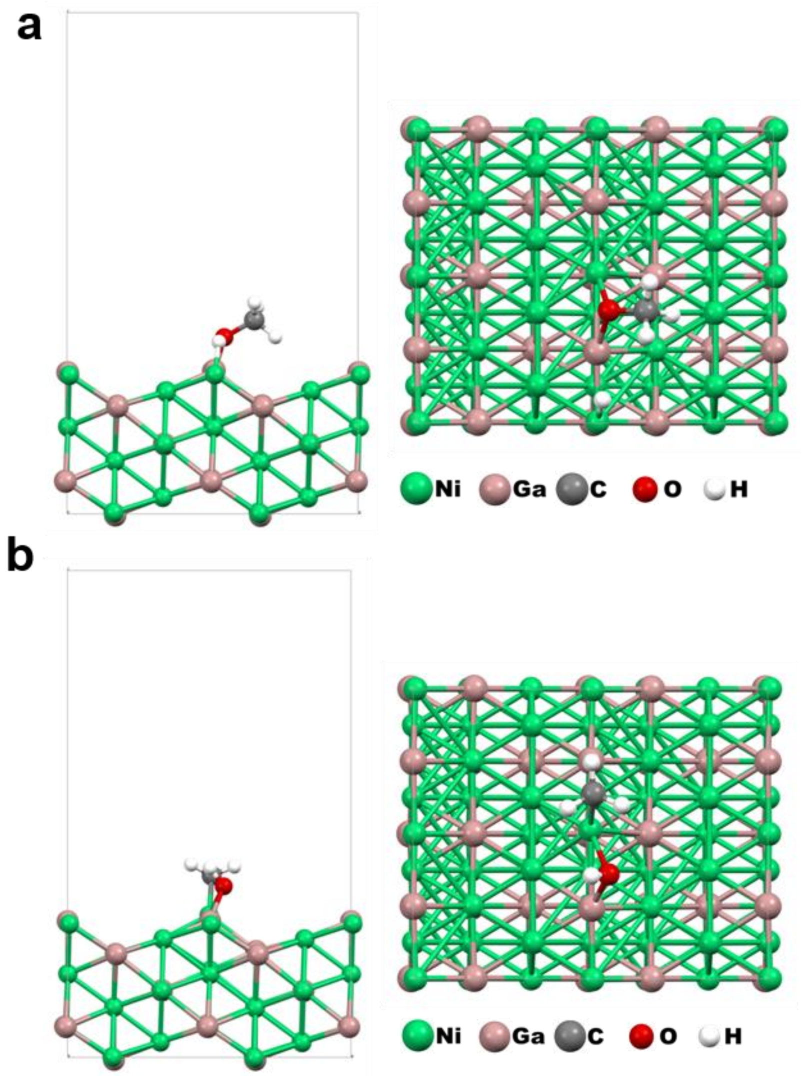

Figure S96. The optimized structure of adsorbed CH_3O* and H* (a) and adsorbed CH_3* and OH* (b) on bimetallic NiGa {211} facet models.

Table S11. Summary of calculated enthalpy change (ΔH_{diss}) of the methoxy dissociation reaction on different metal {211} facet, including monometallic *M* and bimetallic *M*Ga (*M* = Ru, Rh, Ir and Ni) systems based on FCC structure.[a]

	**Ru {211}**	**RuGa {211}**	**Rh {211}**	**RhGa {211}**	**Ir {211}**	**IrGa {211}**	**Ni {211}**	**NiGa {211}**
**E (CH_3O* + H*)**	-672.22	-568.45	-545.10	-490.06	-655.03	-557.39	-410.36	-393.08
**E (CH_3* + OH*)**	-672.79	-568.80	-545.56	-490.28	-655.52	-557.49	-410.69	-393.09
**ΔH_{diss}, eV**	-0.57	-0.35	-0.46	-0.22	-0.49	-0.10	-0.33	-0.01
**ΔH_{diss}, kJ/mol**	-55.00	-33.77	-44.38	-21.23	-47.28	-9.65	-31.84	-0.96

[a] $\Delta H_{diss} = E(CH_3{}^{*} + OH^{*}) - E(CH_3O^{*} + H^{*})$

Reference:

[1] A. K. McMullen, T. D. Tilley, A. L. Rheingold, S. J. Geib, *Inorg. Chem.* **1989**, *28*, 3772-3774.

[2] S. R. Docherty, N. Phongprueksathat, E. Lam, G. Noh, O. V. Safonova, A. Urakawa, C. Coperet, *JACS Au* **2021**, *1*, 450-458.

[3] W. Zhou, S. R. Docherty, C. Ehinger, X. Zhou, C. Coperet, *Chem. Sci.* **2023**, *14*, 5379-5385.

[4] H. Werner, K. Zenkert, *J. Org. Chem.* **1988**, *345*, 151-166.

[5] C. Coperet, A. Comas-Vives, M. P. Conley, D. P. Estes, A. Fedorov, V. Mougel, H. Nagae, F. Nunez-Zarur, P. A. Zhizhko, *Chem. Rev.* **2016**, *116*, 323-421.

[6] K. Searles, G. Siddiqi, O. V. Safonova, C. Copéret, *Chem. Sci.* **2017**, *8*, 2661-2666.

[7] G. R. Fulmer, A. J. M. Miller, N. H. Sherden, H. E. Gottlieb, A. Nudelman, B. M. Stoltz, J. E. Bercaw, K. I. Goldberg, *Organometallics* **2010**, *29*, 2176-2179.

[8] K. A. Lomachenko, A. Y. Molokova, C. Atzori, O. Mathon, *J. Phys. Chem. C* **2022**, *126*, 5175-5179.

[9] B. Ravel, M. Newville, *J. Synchrotron Radiat.* **2005**, *12*, 537-541.

[10] O. V. Dolomanov, L. J. Bourhis, R. J. Gildea, J. A. K. Howard, H. Puschmann, *J. Appl. Crystallogr.* **2009**, *42*, 339-341.

[11] L. J. Bourhis, O. V. Dolomanov, R. J. Gildea, J. A. Howard, H. Puschmann, *Acta Crystallogr. A Found. Adv.* **2015**, *71*, 59-75.

[12] G. Sheldrick, *Acta Crystallogr. C* **2015**, *71*, 3-8.

[13] G. Kresse, J. Furthmüller, *Comput. Mater. Sci.* **1996**, *6*, 15-50.

[14] C. Adamo, V. Barone, *J. Chem. Phys.* **1999**, *110*, 6158-6170.

[15] J. P. Perdew, K. Burke, M. Ernzerhof, *Phys. Rev. Lett.* **1996**, *77*, 3865-3868.

[16] E. Lam, K. Larmier, P. Wolf, S. Tada, O. V. Safonova, C. Coperet, *J. Am. Chem. Soc.* **2018**, *140*, 10530-10535.

[17] E. Lam, G. Noh, K. W. Chan, K. Larmier, D. Lebedev, K. Searles, P. Wolf, O. V. Safonova, C. Coperet, *Chem. Sci.* **2020**, *11*, 7593-7598.

[18] E. Lam, G. Noh, K. Larmier, O. V. Safonova, C. Copéret, *J. Catal.* **2021**, *394*, 266-272.

[19] S. R. Docherty, O. V. Safonova, C. Coperet, *J. Am. Chem. Soc.* **2023**, *145*, 13526-13530.

[20] N. K. Zimmerli, L. Rochlitz, S. Checchiac, C. R. Müller, C. Copéret, P. M. Abdala, *JACS Au* **2024**, *4*, 237-252.

[21] W. Zhou, E. Brack, C. Ehinger, J. Paterson, J. Southouse, C. Coperet, *J. Am. Chem. Soc.* **2024**, *146*, 10806-10811.

[22] S. Kattel, P. Liu, J. G. Chen, *J. Am. Chem. Soc.* **2017**, *139*, 9739-9754.
